# Large language models in bioinformatics: applications and perspectives

**Published:** 2024-01-08

**Authors:** Jiajia Liu, Mengyuan Yang, Yankai Yu, Haixia Xu, Kang Li, Xiaobo Zhou

**Affiliations:** 1 Center for Computational Systems Medicine, McWilliams School of Biomedical Informatics, The University of Texas Health Science Center at Houston, Houston, Texas, 77030, USA; 2 School of Life Sciences, Zhengzhou University, Zhengzhou, Henan 450001, China; 3 School of Computing and Artificial Intelligence, Southwest Jiaotong University, Chengdu, Sichuan 611756, China; 4 The Center of Gerontology and Geriatrics, West China Hospital, Sichuan University, Chengdu, Sichuan 610041, China; 5 West China Biomedical Big Data Center, West China Hospital, Sichuan University, Chengdu, Sichuan 610041, China; 6 McGovern Medical School, The University of Texas Health Science Center at Houston, Houston, TX 77030, USA; 7 School of Dentistry, The University of Texas Health Science Center at Houston, Houston, TX 77030, USA

## Abstract

Large language models (LLMs) are a class of artificial intelligence models based on deep learning, which have great performance in various tasks, especially in natural language processing (NLP). Large language models typically consist of artificial neural networks with numerous parameters, trained on large amounts of unlabeled input using self-supervised or semi-supervised learning. However, their potential for solving bioinformatics problems may even exceed their proficiency in modeling human language. In this review, we will present a summary of the prominent large language models used in natural language processing, such as BERT and GPT, and focus on exploring the applications of large language models at different omics levels in bioinformatics, mainly including applications of large language models in genomics, transcriptomics, proteomics, drug discovery and single cell analysis. Finally, this review summarizes the potential and prospects of large language models in solving bioinformatic problems.

## 1. Introduction

Significant progress has been made in the field of natural language processing with the advent of large language models. Examples of these models include OpenAI’s GPT-X [1] and Google’s BERT [2] models. These models are transformative because they can understand, generate, and manipulate human language at an unprecedented scale. Vast Large language models are typically trained on datasets that encompass a significant portion of the internet’s text, enabling them to learn the complexities of language and context. These models are built upon a neural network architecture called transformers [3]. The transformer architecture revolutionized NLP due to its parallelization, scalability, and ability to capture long-range dependencies in text. Instead of relying on recurrent or convolutional layers, transformers use self-attention mechanisms, as previously described, which allow them to assess the importance of every word in a sentence when understanding context. This innovation is key to their remarkable performance.

**Figure 1.**
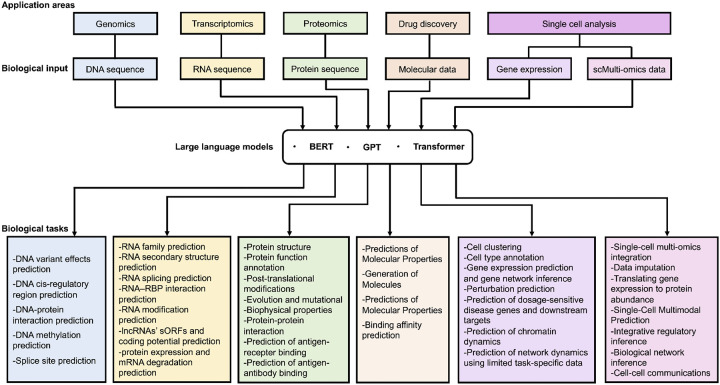
Summary of the application of large language models in bioinformatics in this review. Applications of large language models in bioinformatics include applications in genomics, transcriptomics, proteomics, drug discovery and single cell analysis. Applications of LLMs in genomics focus on LLMs using DNA sequence; applications of LLMs in transcriptomics using RNA sequence; applications of LLMs in proteomics focus on LLMs using protein sequence; applications of LLMs in drug discovery focus on LLMs using molecular data and applications of LLMs in single-cell analysis focus on LLMs using gene expression data from scRNA-seq or scMulti-omics data. Each corresponds to a variety of biological downstream tasks.

The training regimen for large language models comprises two phases: pre-training and fine-tuning. During pre-training, the model is trained on an extensive corpus of text data to acquire proficiency in grammar, factual knowledge, reasoning abilities, and word understanding. Fine-tuning tailors these models for specific tasks like translation, summarization, or question-answering. The adaptability of large language models is a major advantage; they can excel at various NLP tasks without task-specific architectures. However, they have found applications in diverse fields beyond NLP, including biology, healthcare, education, finance, customer service, and more. In particular, there have been many successful applications of large language models in the field of bioinformatics. In this manuscript, we focus on the applications of large language models to several bioinformatic tasks through five areas: DNA level, RNA level, protein level, drug discovery and single cell analysis, respectively corresponding to sections: applications of large language models in genomics, transcriptomics, proteomics, drug discovery and single cell analysis. Applications of LLMs in genomics focus on LLMs using DNA sequence; applications of LLMs in transcriptomics using RNA sequence; applications of LLMs in proteomics focus on LLMs using protein sequence; applications of LLMs in drug discovery focus on LLMs using Molecular SMILES (seq) and applications of LLMs in single-cell analysis focus on LLMs using gene expression data from scRNA-seq or scMulti-omics data (Figure 1).

## 2. Large language models in natural language processing

**Figure 2.**
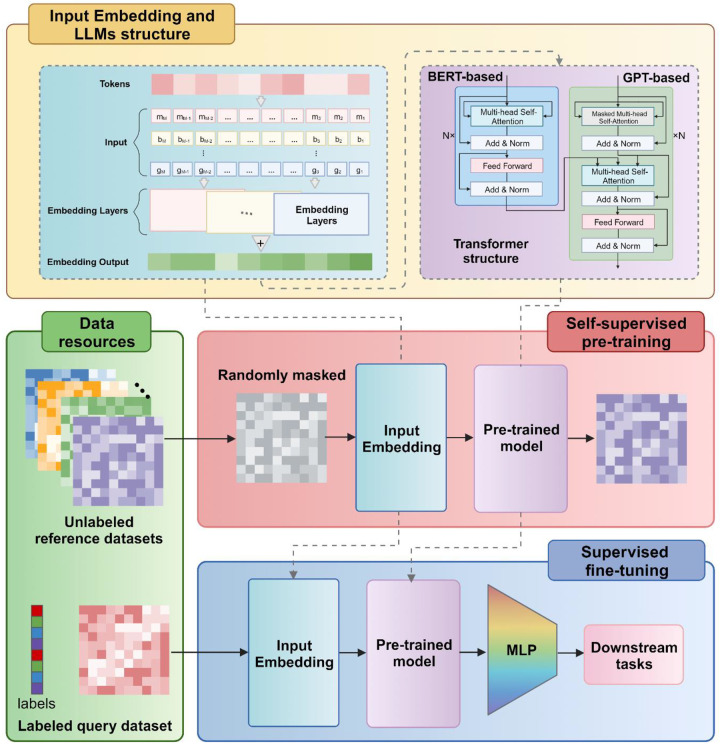
Schematic diagram of large language model. The input of large language models is tokenized and fed into embedding layers. Large language models, particularly exemplified by BERT-based (transformer encoder) and GPT-based (transformer decoder) models, their core is attention mechanism. The training process of large language models usually includes pre-training and fine-tuning. Pre-training is mainly to train a general/foundation model with strong generalization ability on a large-scale unlabeled reference dataset using self-supervised learning. Fine-tuning relies on the pre-trained model, undergoing additional training for a specific task.

The emergence of large language models has brought milestone progress to natural language processing. These large language models, particularly exemplified by BERT [2] and GPT [1], usually use the “pre-training + fine-tuning” approach (Figure 2). Pre-training is mainly to train a general/foundation model with strong generalization ability on a large-scale text corpus. Fine-tuning relies on the pre-trained model, undergoing additional training for a specific task. This process allows the model to adjust to the unique data and demands of the task at hand. The goal of fine-tuning is to improve the performance of the model on specific tasks, such as sentiment analysis, question answering systems, abstract text summarization, machine translation, creative text generation, etc. in natural language processing.

### Self-attention.

At the heart of the transformer is self-attention [3]. In this mechanism, every word/token in the input sequence is linked to three vectors: the Key vector (K), the Query vector (Q), and the Value vector (V). These vectors are learned during the training of the model. For each token, the model calculates attention scores for its relationship with other tokens. The attention score between a Query (Q) and Key (K) pair is calculated through the dot product, which is then adjusted by scaling it down with the square root of the dimension of the key vectors (to prevent exceedingly large values). Subsequently, a softmax function is frequently applied to attain normalized scores. The attention score is then multiplied by the Value (V) vector to derive the representation through the weighted sum of the attention mechanism. The self-attention process is represented as following [3]:

(1) $$\text{Attention}(Q,K,V)=\text{softmax}(\frac{QK^{T}}{\sqrt{d_{k}}})V$$

### BERT (Bidirectional Encoder Representations from Transformers).

BERT stands as a revolutionary model in the realm of natural language processing, reshaping the landscape of deep learning. Introduced by Google in 2018, BERT is engineered to comprehend the context and subtleties of human language in unprecedented ways [2]. At its core, BERT employs a Transformer architecture, which enables it to capture the relationships between words in both directions of a sentence, making it “bidirectional”. This implies that BERT considers not only the words preceding a given word but also those following it, allowing it to grasp the full meaning of a sentence. One of BERT’s most remarkable features is its pretraining process. It learns from an extensive corpus of text from the internet, effectively absorbing vast amounts of knowledge. The pre-trained model can subsequently undergo fine-tuning for diverse natural language understanding tasks, including text classification, question answering, and language translation. BERT has consistently demonstrated state-of-the-art performance across a broad spectrum of NLP tasks and benchmarks, thanks to its ability to handle context, polysemy, and long-range dependencies effectively. Its versatility and accuracy have made it a pivotal tool in various applications, from chatbots and virtual assistants to sentiment analysis and content recommendation systems.

### GPT (Generative Pretrained Transformer).

GPT stands as a remarkable achievement in the realm of natural language processing. Introduced by OpenAI, GPT is a sophisticated language model designed to generate human-like text, making it a pivotal component in a wide array of applications [1]. At its core, GPT is built upon the transformer architecture, renowned for its attention mechanisms that allow it to comprehend the nuances of context and language structure. What sets GPT apart is its “pretraining” phase, where it’s exposed to an extensive corpus of text from the internet, enabling it to absorb an immense amount of linguistic knowledge. The real brilliance of GPT lies in its capacity to produce text that is both coherent and contextually relevant. Given a prompt, it can produce everything from creative stories and natural-sounding dialogue to summaries and translations. This versatility has found applications in chatbots, content generation, language translation, and even code generation. GPT’s knack for understanding context, handling ambiguity, and generating human-like text has earned it a place of honor in various domains, from creative writing and customer support to academic research and data analysis. Its adaptability, powered by fine-tuning, makes it a potent tool for diverse language-related tasks.

### Comparison between BERT and GPT.

BERT and GPT stand as two exceptional language models that have transformed the landscape of Natural Language Processing (NLP). While they share some common traits, they also exhibit significant differences in their design and applications. Both BERT and GPT leverage the transformer architecture, employing attention mechanisms to grasp contextual information and dependencies within text data. This architecture has demonstrated significant effectiveness in comprehending and generating text that resembles human language. Furthermore, both models go through a pretraining phase, during which they are exposed to extensive amounts of text data sourced from the internet. This unsupervised learning process helps them learn language structure, grammar, and a broad range of linguistic patterns. Furthermore, both BERT and GPT excel at transfer learning. These models undergo pretraining on an extensive text corpus and can be fine-tuned for particular downstream tasks, including text classification, sentiment analysis, or language generation. This makes them versatile and applicable across various NLP tasks.

However, there are notable differences between these models. BERT employs a bidirectional approach, considering both left and right contexts when learning language representations. It aims to create deep contextual embeddings for words. In contrast, GPT uses a left-to-right approach, generating text autoregressively by predicting the next word in a sequence based on the preceding words. This design makes GPT more suitable for generation tasks. Additionally, their architectures differ, with BERT using a bidirectional Transformer encoder and a masked language model (MLM) objective during pretraining, while GPT uses a unidirectional transformer decoder and a causal language model objective. BERT undergoes training through a masked language model task and next sentence prediction, whereas GPT (GPT-1) is trained to predict the succeeding word in a sequence. In practice, BERT is often preferred for tasks requiring deep contextual understanding, such as question-answering, sentiment analysis, and text classification. It is widely used for tasks where understanding the context of words is crucial. On the other hand, GPT shines in text generation tasks, including creative writing, language translation, and dialogue generation. It is an excellent choice for tasks that prioritize generating human-like text.

## 3. Applications of large language models in bioinformatics

### 3.1 Applications of large language models in genomics

**Figure 3.**
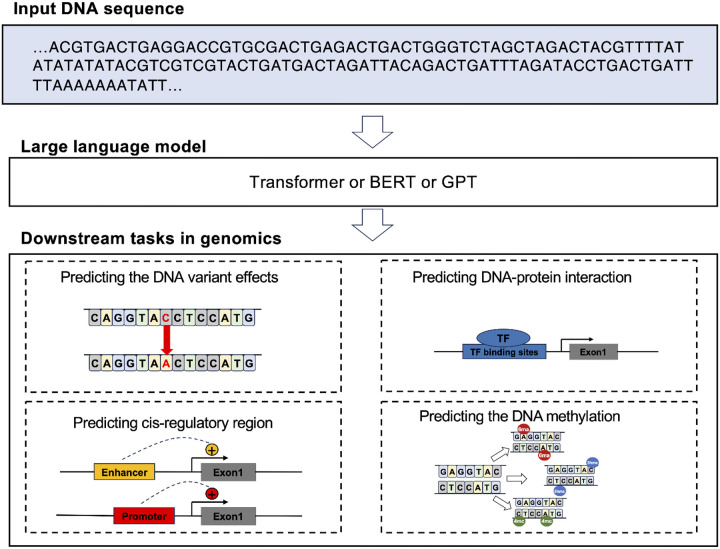
Applications of large language models in genomics. The DNA language models take DNA sequence as input, use transformer, BERT, GPT models to solve multiple biological tasks, including genome-wide variant effects prediction, DNA cis-regulatory regions prediction, DNA-protein interaction prediction, DNA methylation (6mA,4mC 5hmC) prediction, splice sites prediction from DNA sequence.

Decoding the language embedded in DNA to unveil its concealed instructions has long stood as a primary goal in biological research [4]. The universal genetic code, elucidating the translation of DNA into proteins, has recently garnered attention for deciphering biological functions through models based on BERT or GPT architectures. DNABERT [5] for instance, employs a transformer—a robust, attention-based architecture renowned for its prowess in diverse natural language processing tasks. An evolution of this, DNABERT-2[6] introduces enhancements such as an efficient tokenizer and strategies to address input length constraints, thereby optimizing time and memory usage while bolstering model capabilities. Notably, DNABERT-2 introduces the Genome Understanding Evaluation (GUE), a comprehensive dataset for multi-species genome classification. With a threefold increase in efficiency compared to DNABERT, DNABERT-2 outperforms its predecessor on 23 out of 28 datasets. GROVER [7], a DNA language model leveraging byte-pair tokenization to scrutinize the human genome. GROVER’s core objective lies in discerning contextual relationships between tokens, facilitating the identification of genomic region structures associated with functional genomics annotation. This unique approach positions GROVER as an invaluable tool for researchers delving into the intricacies of the human genome. Building on the success of the GPT series in extracting general information from DNA sequences, DNAGPT emerges as a GPT-based model for DNA, pre-trained on a vast dataset exceeding 10 billion base pairs. This model can be finely tuned for various DNA sequence analysis tasks. Additionally, there is the Nucleotide Transformer [8], as another foundational model for DNA sequences, has developed four distinct language models of varying sizes. These models have been pre-trained on three different datasets spanning multiple species. DNABERT, DNABERT-2, GROVER, DNAGPT, and the Nucleotide Transformer, all being pre-trained models, find application in sequence prediction tasks, including predicting promoter regions, enhancer regions, cis-regulatory elements, splice sites, and transcription factor binding sites. Detailed information is available in Figure 3, Table 1, and **Supplementary Table 1**.

#### 3.1.1 DNA sequence language models used to predict the genome-wide variant effects

The significance of DNA sequence mutations in fostering diversity both within and between species cannot be overstated. Genome-wide association studies (GWAS) have played a pivotal role in furnishing essential biological insights across various species. However, a persistent challenge lies in pinpointing the specific causal variants accountable for the associations uncovered in these studies [4, 9]. The Genomic Pre-trained Network (GPN) [10] is focused on acquiring knowledge about genome-wide variant effects by undergoing unsupervised pre-training on genomic DNA sequences. In this process, the model is presented with a 512-bp DNA sequence wherein certain positions are intentionally masked. Its primary objective is to predict the nucleotides at these masked positions. Notably, GPN excels in predicting the effects of rare variants that conventional GWAS methods may overlook. Leveraging the DNA sequence of any species, GPN demonstrates its capacity to predict variant effects across the entire genome. Furthermore, foundational models within the DNA sequence language model realm, such as DNABERT, DNABERT-2, and the Nucleotide Transformer, also exhibit the capability to predict variants from DNA sequences. This collective advancement underscores the ongoing efforts to enhance our understanding of the intricate relationship between DNA sequence mutations and the resultant diversity in biological landscapes.

#### 3.1.2 DNA sequence language models used to predict the cis-regulatory regions

Cis-regulatory sequences, including enhancers and promoters, represent pivotal elements in the regulation of gene expression, exerting a significant impact on the development and physiology [11]. However, identifying these sequences in DNA represents a major challenge, which is essential to comprehend their functions and their direct or indirect association with various diseases [12]. To address this issue, pre-trained models such as DNABERT, DNABERT-2, GROVER, and DNAGPT have been developed to accurately predict the promoter regions and their activities. These models have demonstrated remarkable accuracy and have emerged as valuable tools in the field of molecular biology for identifying cis-regulatory regions in DNA, and thus providing useful information on their functions and related diseases. BERT-Promoter [13] was proposed to identify promoters and their activity. It uses a pre-trained BERT model for efficient feature representation and SHAP analysis for filtering. Different machine-learning algorithms are subsequently applied to construct the final prediction model. Notably, BERT features in BERT-Promoter demonstrate significant performance improvement and enhanced model generalization compared to traditional feature representations.

Enhancers, small DNA segments binding to transcription factor proteins, play a crucial role in fortifying gene transcription and influencing gene expression [14, 15]. iEnhancer-BERT [16] presents an innovative transfer learning approach leveraging DNABERT to facilitate enhancer prediction. Departing from conventional fine-tuning methods, iEnhancer-BERT utilizes the output of all transformer encoder layers to generate feature vectors. These vectors undergo further classification through a Convolutional Neural Network (CNN) layer. Recognizing biological sequences as the natural language for computational modeling signals an emerging trend in the field. In conclusion, iEnhancer-BERT presents a promising avenue for identifying new DNA enhancers.

#### 3.1.3 DNA sequence language models used to predict the DNA-protein interaction

The accurate identification of DNA-protein interactions is vital for regulating gene expression and understanding evolutionary processes [17]. Several DNA language models have been developed to predict these interactions, with downstream tasks for DNABERT, DNABERT-2, and GROVER including the prediction of protein-DNA binding based on ChIP-seq data. Besides, TFBert [18] is a pre-training DNA-protein binding model that has the ability to provide satisfactory results with minimal fine-tuning on a single dataset. This model treats DNA sequences as natural sentences and k-mer nucleotides as words, allowing for the effective extraction of context information from upstream and downstream nucleotides. Through pre-training on the 690 ChIP-seq datasets, TFBert can efficiently accomplish this task. MoDNA [19] framework is an innovative approach that incorporates common DNA functional motifs as domain knowledge. In the initial stage of self-supervised pre-training, the MoDNA framework establishes two prediction tasks including k-mer tokens prediction and motif prediction. Through pre-training on vast amounts of unlabeled genome data, the MoDNA framework successfully acquires semantic-level genome representations, which prove useful for promoter prediction and transcription factor binding site prediction. In essence, the MoDNA framework can be regarded as a biological language model that predicts DNA-protein binding.

#### 3.1.4 DNA sequence language models used to predict the DNA methylation.

DNA methylation stands as a fundamental biological process, playing a pivotal role in the epigenetic regulation of gene expression [20]. This process is linked to various medical conditions and finds diverse applications, such as serving as a marker for metagenomic binning. The types of DNA methylation vary based on the specific nucleotide to which the methyl group attaches in the sequence [21]. Several models have been developed to predict DNA methylation, with varying degrees of accuracy and specificity. The advancement of these models has led to a better understanding of the mechanisms underlying DNA methylation and its implications in various biological processes. Among them, the BERT6mA [22] is specifically designed for predicting 6-methyadenine (6mA) sites. iDNA-ABT [23], iDNA-ABF [24], and MuLan-Methyl [25] are versatile predictors that can be used for various methylation predictions, including 6mA, 5-hydroxymethylcytosine (5hmC), and 4-methylcytosine (4mC). iDNA-ABT, an advanced deep learning model, incorporates adaptive embedding based on BERT along with transductive information maximization (TIM). While demonstrating potential in identifying species, iDNA-ABT has yet to fully explore feature representation learning’s potential, especially in uncovering key sequential patterns critical for understanding DNA methylation mechanisms. The iDNA-ABF approach adopts a multi-scale architecture, using multiple tokenizers instead of a single one. This enables BERT encoders to extract diverse embeddings based on tokenization, which are then combined to generate the final evolutionary output feature.

On the other hand, MuLan-Methyl introduces a novel deep-learning framework employing five transformer-based language models—BERT[2], DistilBERT[26], ALBERT[27], XLNet[28], and ELECTRA[29] —to predict three types of methylation sites from DNA sequences and taxonomic information. The model generates its output by averaging the prediction probabilities from these language models. It’s noteworthy that language models find successful application in biological sequence analysis, and the joint utilization of different models significantly enhances performance.

#### 3.1.5 DNA sequence language models used to identify the splice site.

The precise splicing of pre-mRNA is crucial for ensuring accurate protein translation. This intricate process is governed by the selection of splice sites during splicing reactions, leading to the creation of diverse isoforms and splicing events. However, identifying splice sites poses a challenge, especially considering the prevalent GT-AG sequences in the DNA [30]. In response to this challenge, DNABERT and DNABERT-2 were developed and trained using 10,000 donor, acceptor, and non-splice site sequences from the human reference genome. The objective was to predict splice sites from DNA sequences. Notably, DNABERT consistently exhibited high attention to intronic regions. This observation suggests the presence and functional significance of various intronic splicing enhancers and silencers, acting as cis-regulatory elements for splicing. The focus on intronic regions underscores the importance of these elements in modulating splicing outcomes and highlights the potential of DNABERT models in unraveling the complexities of splicing regulation.

### 3.2 Applications of large language models in transcriptomics

**Figure 4.**
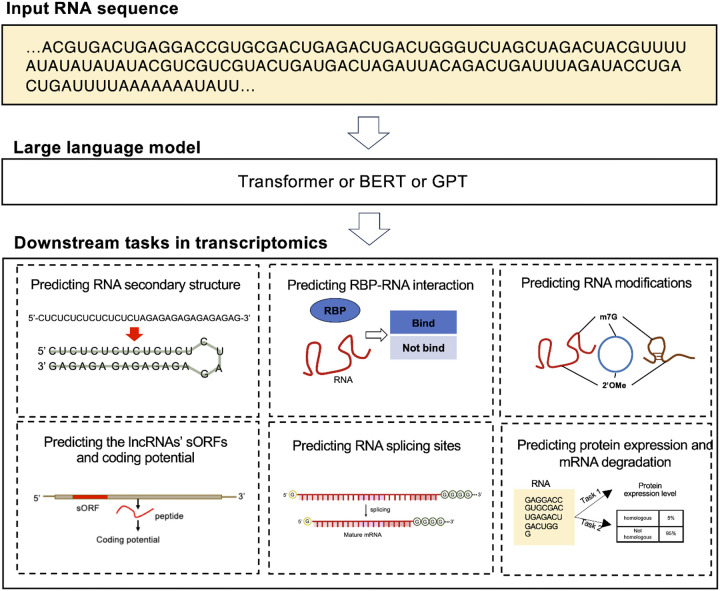
Applications of large language models in transcriptomics. The RNA language models take RNA sequences as input, use transformer, BERT, GPT models to solve multiple biological tasks, including RNA 2D/3D structure prediction, RNA structural alignment,, RNA family clustering, RNA splice sites prediction from RNA sequence, RNA N7-methylguanosine modification prediction, RNA 2’-O-methylation modifications prediction, multiple types of RNA modifications prediction, predicting the association between miRNA, lncRNA and disease, identifying lncRNAs, lncRNAs’ coding potential prediction, protein expression and mRNA degradation prediction.

Efforts to develop BERT-based language models for DNA have faced challenges in accurately capturing evolutionary information from homologous sequences. Unlike proteins, RNA sequences are less conserved. In response to this, two notable RNA foundation models have been introduced: RNA-FM [31] and RNA-MSM [32]. RNA-FM employs self-supervised learning to predict secondary/3D structures, leveraging the vast dataset of all 23 million non-coding RNA sequences. This approach allows RNA-FM to effectively capture diverse structural information, providing a comprehensive understanding of RNA sequence features. On the other hand, RNA-MSM utilizes homologous sequences sourced from RNAcmap through an automated pipeline. This model excels in accurately mapping to 2D base pairing probabilities and 1D solvent accessibilities. The pre-trained model can be fine-tuned for various downstream tasks related to RNA structure and function, as shown in Figure 4, Table 1, and **Supplementary Table 1**.

#### 3.2.1 RNA sequence language models used to predict the RNA family classification and secondary structure

RNA secondary structure prediction poses a substantial challenge for RNA structural biologists, requiring focused efforts to better understand RNA folding rules and enhance the accuracy of structure prediction models. Such models hold significant potential for facilitating downstream tasks, including the development of RNA-targeting drugs [33]. RNABERT [34] is designed with three key components: tokens and position embedding, a transformer model, and pre-training tasks. Leveraging the pre-training BERT algorithm for non-coding RNA (ncRNA), RNABERT is specifically tailored for secondary structure prediction and RNA family classification. The model’s architecture and training tasks are strategically crafted to capture the intricate rules governing RNA folding, enabling more accurate predictions. One notable application of RNABERT lies in addressing the practical need for rapid and precise structural alignment of unknown sequences to existing RNA families. This capability makes RNABERT a valuable tool for annotating novel transcripts, assisting researchers in understanding the structural characteristics of previously uncharacterized RNA molecules. The potential contributions of RNABERT extend beyond structure prediction, offering practical solutions for tasks critical to advancing our knowledge of RNA biology and its applications in therapeutic development.

#### 3.2.2 RNA sequence language models used to predict the RNA splicing

RNA splicing is a vital process in the post-transcriptional gene expression of eukaryotic organisms. Researchers have made strides in enhancing the sequence-based modeling of RNA splicing through the development of a pre-trained model known as SpliceBERT [35]. This model is trained on precursor messenger RNA sequences derived from 72 vertebrates, enabling it to generate embeddings that preserve both the evolutionary information of nucleotides and the functional characteristics of splice sites. SpliceBERT serves a dual purpose by not only capturing the nuances of RNA splicing but also facilitating the identification of potential splice-disrupting variants. The pre-trained model allows for the unsupervised prioritization of such variants based on their impact on the output of SpliceBERT within the sequence context. This capability offers a valuable tool for researchers seeking to understand the influence of genetic variations on RNA splicing, providing insights that can aid in the identification and prioritization of potentially significant variants in an efficient and data-driven manner.

#### 3.2.3 RNA sequence language models used to identify the lncRNAs and predict the lncRNAs’ coding potential

Long non-coding RNA (lncRNA) is a crucial transcript form that plays a substantial regulatory role in the development of cancers and diseases without encoding proteins [36]. Initially, small Open Reading Frames (sORFs) in lncRNA were thought to be weak in protein translation. However, recent studies have revealed that they can indeed encode peptides. This discovery adds complexity to the identification of lncRNA, particularly those containing sORFs, which is crucial for uncovering novel regulatory factors. Addressing this challenge, LncCat [37] utilizes category boosting and ORF-attention features for improved performance on both long ORF and sORF datasets. The ORF-attention feature positively influences the prediction of lncRNA. LncCat employs the BERT model to represent peptide sequences encoded by ORFs as part of the ORF-attention features. CatBoost[38] is utilized to build the prediction model, incorporating the aforementioned features, to identify lncRNA. The effectiveness of LncCat is demonstrated across five species datasets and the Ribo-seq dataset, showcasing its utility in accurately identifying lncRNA with sORFs and contributing to the discovery of novel regulatory elements.

The prediction of translatable sORFs within lncRNAs, referred to as lncRNA-sORFs, is crucial f or expediting the discovery of peptides encoded by these RNAs. Computational prediction metho ds play a vital role in this task. In this context, LSCPP-BERT (https://github.com/Sakuraxia/LSC PP-BERT) emerges as a novel method designed for predicting the coding potential of lncRNA-s ORFs in plants. Leveraging pre-trained bidirectional encoder representations from transformer m odels, LSCPP-BERT offers a reliable tool for predicting coding lncRNA-sORFs and holds the po tential to significantly contribute to drug development and agricultural applications by enhancing our understanding of the coding potential within lncRNAs.

#### 3.2.4 RNA sequence language models used to predict the RNA–RBP interactions

RNA sequences differ from DNA sequences by a single base (thymine to uracil), resulting in a singular variance where the syntax and semantics largely remain congruent. The versatility of BERT extends beyond DNA to encompass Cross-linking and Immunoprecipitation (CLIP-seq) data, providing a valuable tool for predicting the binding preferences of RNA-binding proteins (RBPs). BERT-RBP [39] is a model specifically designed for forecasting RNA-RBP interactions. It adapts the BERT architecture and is pre-trained on a human reference genome. BERT-RBP outperforms contemporary prediction models when assessed against eCLIP-seq data from 154 RBPs. Additionally, the model exhibits the ability to discern both transcript region types and RNA secondary structures based solely on sequence information. In essence, BERT-RBP not only contributes insights into the fine-tuning mechanisms of BERT in biological contexts but also provides compelling evidence of the model’s versatility in addressing various challenges related to RNA, showcasing its potential in advancing our understanding of RNA-protein interactions.

#### 3.2.5 RNA sequence language models used to predict the RNA modification

Post-transcriptional modifications of RNA play a crucial role in a diverse array of biological processes. Among these modifications, N7-methylguanosine (m7G) stands out as one of the most prevalent, playing an essential role in gene expression regulation [36]. The precise identification of m7G sites within the transcriptome is of immense value for a comprehensive understanding of their potential functional mechanisms. While high-throughput experimental methods offer precise localization of m7G sites, their costliness and time-consuming nature present challenges. In response to this, BERT-m7G [40] emerges as a transformative computational tool. Grounded in the transformer architecture of BERT and utilizing stacking ensemble techniques, BERT-m7G excels at identifying RNA N7-methylguanosine sites solely from RNA sequence information. This computational method proves imperative in accurately pinpointing m7G sites, providing a more efficient alternative to labor-intensive experimental approaches. BERT-m7G showcases the power of computational approaches in unraveling post-transcriptional modifications, offering a valuable tool for researchers seeking to understand the functional implications of m7G in gene expression regulation.

The post-transcriptional 2’-O-methylation (Nm) RNA modification plays a significant role in diverse cellular processes and is linked to several diseases [41]. To gain profound insights into the underlying biological mechanisms, the Bert2Ome [42] method proves to be an efficient tool for inferring 2’-O-methylation RNA modification sites directly from RNA sequences. Bert2Ome integrates a BERT-based model with Convolutional Neural Networks (CNN) to discern the intricate relationship between modification sites and the content of RNA sequences. This innovative approach not only reduces the time required for labor-intensive biological experiments but also surpasses existing methodologies across various datasets and species, demonstrating superior performance across multiple metrics. Bert2Ome showcases the power of computational methods in advancing our understanding of post-transcriptional RNA modifications, providing a valuable tool for researchers exploring the role of 2’-O-methylation in diverse cellular contexts and diseases.

#### 3.2.6 RNA sequence language models used to predict protein expression and mRNA degradation

mRNA vaccines have emerged as a cost-effective, swift, and secure alternative to traditional vaccines, displaying high potency[43]. The mechanism of action for mRNA vaccines involves introducing a segment of mRNA corresponding to a viral protein, typically derived from the virus’s outer membrane. Thus, CodonBERT[44] has been specifically designed for mRNA sequences to predict protein expression. Utilizing a multi-head attention transformer architecture framework, CodonBERT underwent pre-training on a vast dataset comprising 10 million mRNA coding sequences from diverse organisms. This extensive pre-training equips CodonBERT to excel in various mRNA prediction tasks, including protein expression and mRNA degradation prediction. One of CodonBERT’s notable strengths lies in its capacity to assimilate new biological information, positioning it as an asset for advancing mRNA vaccine design. By surpassing existing state-of-the-art methods, CodonBERT contributes to optimizing mRNA-based vaccine development, promising improved efficacy and broader applicability in the realm of immunization. The model’s proficiency in predicting protein expression levels enhances its utility in designing mRNA sequences for vaccines, ultimately impacting the efficiency and effectiveness of the vaccine development process.

### 3.3 Applications of large language models in proteomics

Protein is an indispensable molecule in life, assuming a pivotal role in the construction and sustenance of vital processes. As the field of protein research advances, there has been a substantial surge in the accumulation of protein data [45]. In this context, the utilization of large language models emerges as a viable approach to extract pertinent and valuable information from these vast reservoirs of data. Several pre-trained protein language models (PPLMs) have been proposed to learn the characteristic representations of proteins data (e.g., protein sequences, gene ontology annotations, property descriptions), then applied to different tasks by fine-tuning, adding or altering downstream networks, such as protein structure, post-translational modifications (PTMs), and biophysical properties, which align with corresponding downstream tasks like secondary structure prediction, major PTMs prediction, and stability prediction [46, 47].

**Figure 5.**
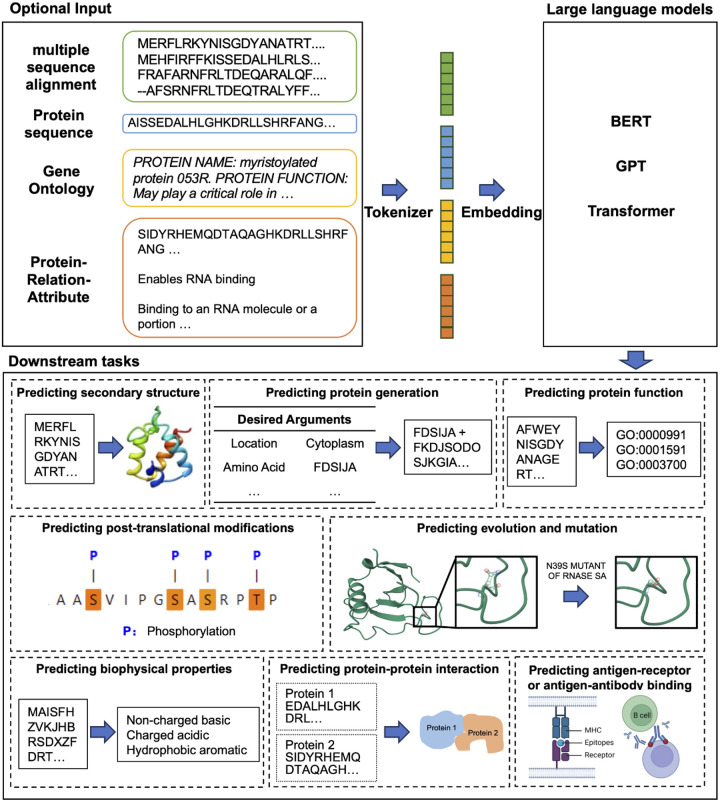
Applications of large language models in proteomics. The protein language models take multiple sequence alignment, protein sequence, gene ontology and protein-relation-attribute as input, use transformer, BERT, GPT models to solve multiple biological tasks, including predicting secondary structure, predicting protein generation, predicting protein function, predicting post-translational modifications, predicting evolution and mutation, predicting biophysical properties, predicting protein-protein interaction and predicting antigen-receptor or antigen-antibody binding.

Even though antibodies are classified as proteins, the datasets of antibodies and subsequent tasks differ significantly from those of proteins. Through the establishment and continuous updates of the Observed Antibody Space (OAS) database [48], a substantial amount of antibody sequence data has become available, which can be utilized to facilitate the development of pre-trained antibody large language models (PALMs). PALMs primarily delve into downstream topics encompassing therapeutic antibody binding mechanisms, immune evolution, and antibody discovery, which correspond to tasks like paratope prediction, B cell maturation analysis, and antibody sequence classification (Figure 5).

In this section, some of the popular protein-related large language models of recent years are introduced, as well as corresponding important downstream tasks. It is important to emphasize that the capabilities of both PPLMs and PALMs extend beyond the specific downstream tasks outlined in this section. For further details, additional information can be referenced within Table 2 and **Supplementary Table 2**.

#### 3.3.1 Protein language models for secondary structure and contact prediction

The structure of proteins plays a crucial and decisive role in their function and interactions [49]. Nonetheless, the conventional laboratory-based techniques employed for protein structure analysis are frequently characterized by their time-consuming, labor-intensive nature. In addition to traditional template-based and physics-based methods, with the development of deep learning, the use of large language models to predict protein structures has gradually shown advantages in computational speed and prediction accuracy [50].

MSA Transformer [51] presents a protein language model that takes a set of sequences as input in the form of a multiple sequence alignment (MSA). This model employs a unique mechanism of interleaved row and column attention across the input sequences. After model trained with a variant of MLM objective across many protein families, it outperformed other unsupervised approaches at the time. Furthermore, it exhibits superior parameter efficiency compared to prior state-of-the-art protein language models. When using PPLMs to predict secondary structure or contact, based on the experience brought by BERT, it seems that using a language model with a larger number of parameters is easier to achieve better performance. Few models seem to have more parameters than the largest models in ProtTrans [52]. ProtTrans trains a series of large language models based on two autoregressive models (Transformer-XL [53], XLNet [28]) and four automatic encoder models (BERT [2], Albert [27], Electra [29], T5 [54]) on data from UniRef [55] and BFD [56] [57] containing up to 393 billion amino acids. The parameters of models range from millions to billions. In addition to predicting secondary structure, it is worth highlighting that ProtTrans achieved a significant breakthrough in per-residue predictions. For the first time, the transfer of the most informative embeddings (ProtT5) outperformed the state-of-the-art methods without relying on evolutionary information, thus bypassing the need for costly database searches.

#### 3.3.2 Protein language models for protein sequence generation

Generation of protein has broad application prospects in fields such as drug design and protein engineering [58]. By using methods such as machine learning or deep learning, protein sequences can be generated. The generated sequences are hoped to have good foldability so that they can form stable three-dimensional structures. Moreover, the desired proteins are expected to exhibit specific functional properties, including enzyme activity and antibody binding capability. The advancement of large language models and the integration of conditional models have significantly propelled the progress in the field of protein generation [59].

The model, referred to as ProGen [60], incorporates UniprotKB Keywords as conditional tags in 2020. These tags encompass a vocabulary consisting of various categories, including ‘biological process’, ‘cellular component’, and ‘molecular function’. In total, the conditional tags encompass over 1,100 distinct terms. When assessing protein sequences generated by ProGen using metrics for sequence similarity, secondary structure accuracy, and conformational energy, they exhibit desired structural properties. In 2022, inspired by the remarkable achievements of generative Transformer-based language models like the GPT-x series, the development of ProtGPT2 [61] emerged. Notably, the proteins generated by ProtGPT2 exhibit amino acid propensities t following the principles of natural ones. Assessments involving disorder and secondary structure prediction reveal that a substantial majority (88%) of ProtGPT2-generated proteins possess globular characteristics, aligning with the attributes found in natural sequences. Employing AlphaFold [62, 63] on ProtGPT2 sequences yields well-folded non-idealized structures, encompassing the presence of extensive loops, and the emergence of previously unseen topologies that are absent from current structure databases. It appears that ProtGPT2 has acquired the language specific to proteins.

#### 3.3.3 Protein language models for protein function prediction

Proteins are molecules that play a crucial role in various aspects of cellular metabolism, signal transduction, and structural support in living organisms. A deep understanding of the function of proteins in living organisms is of great significance for drug development and disease mechanism analysis. The diversity and complexity of proteins make it difficult to accurately predict and annotate their functions. Fortunately, PPLMs can effectively address these challenges [64, 65]. Taking into account the substantial presence of local semantics within protein sequences, a novel approach to pre-training modeling, referred to as SPRoBERTa [66], was introduced in 2022. This method takes protein sequences as inputs and offers the flexibility of straightforward fine-tuning for diverse protein-related tasks, encompassing prediction tasks at the protein-level tasks (such as remote homology prediction and protein function prediction), as well as amino acid level (e.g., secondary structure prediction) and amino acid pair-level (e.g., contact prediction). In the next year, ProtST [67] introduced a multimodal training framework for proteins, which involves the integration of a protein language model (PLM) whose input is protein sequences and a biomedical language model (BLM) whose input is protein property descriptions into a large multimodal model. This integration is achieved through three pre-training tasks: unimodal mask prediction, multimodal representation alignment, and multimodal mask prediction. The proposed model demonstrates exceptional performance in diverse downstream tasks related to protein representation. In addition to finishing the task of protein function annotation, it shows the effectiveness on zero-shot protein classification. Furthermore, the model possesses the capability to facilitate the retrieval of functional proteins from a vast-scale database, even in the absence of any functional annotation.

#### 3.3.4 Protein language models for major post-translational modification prediction

Post-translational modification (PTM) refers to the process in which the structure and function of proteins are changed through a series of chemical modification reactions after translation is completed, including various chemical changes such as phosphorylation, methylation, acetylation, and glycosylation of proteins. These modifications can significantly impact protein stability, subcellular localization, interactions, and functional expression. In-depth investigation of PTMs yields valuable insights for disease diagnosis and therapeutic interventions [68, 69]. Language models can perform tasks such as signal peptide prediction and major PTMs prediction effectively. ProteinBERT [70] is not really a large language model in terms of parameters (only ~ 16M), but thanks to the introduction of the GO annotation prediction task and the interaction of GO with protein sequences, compared with other deep learning models with larger parameters, this model has achieved considerable or even better performance on multiple benchmarks covering diverse protein properties including major PTMs prediction.

#### 3.3.5 Protein language models for evolution and mutation prediction

During the process of biological evolution, the sequence and structure of proteins undergo changes. Evolution and mutation serve as vital mechanisms that generate functional diversity in proteins [71]. Gaining insights into the process of protein evolution and mutation can reveal strategies for organisms to adapt to environmental changes and survival competition, as well as the origin and evolution of protein function, and provide new ideas for drug development and disease treatment [72].

In the context of protein sequence inputs, early protein language models treated an entire sequence as either a paragraph or a sentence, with individual amino acids representing individual words [73, 74]. In 2019, a model known as UniRep [75], built upon the Long Short-Term Memory (LSTM) architecture emerged. This model underwent training using the UniRef50 [55] dataset and exhibited a remarkable improvement in efficiency, surpassing other models in several tasks including remote homology detection and mutational effect prediction. Since 2020, more and more large language models of protein have been proposed to perform prediction in evolution and mutation. In 2020, a deep transformer model known as ESM-1b [76] underwent training on a vast and diverse dataset comprising 250 million sequences. This training enabled the model to acquire protein sequence representations encompassing essential characteristics. The architecture of the model comprised 33 layers, housing approximately 650 million parameters. To facilitate its training, ESM-1b utilized self-supervised strategy, masking language modeling objective. This approach allowed the model to learn and capture crucial patterns and dependencies within the protein sequences, thereby enhancing its overall performance and representation capabilities.

#### 3.3.6 Protein language models for biophysical properties prediction

The biophysical properties of proteins include fluorescence landscapes, stability landscapes, and so on [77]. Accurate prediction of these properties plays a crucial role in advancing our understanding of protein folding mechanisms, stability, conformational alterations, and so on. This is of great significance for the development of drug design, protein engineering, enzyme engineering, and other relevant fields. The progressive advancements in deep learning have enabled the efficient utilization of the continuously evolving PPLMs for the precise prediction of biophysical properties associated with proteins.

A significant development known as Tasks Assessing Protein Embeddings (TAPE) [78] emerged in 2019, which introduced a comprehensive benchmark of protein bioinformatics tasks. This paper aimed to establish a standardized evaluation system for protein transfer learning by providing well-defined tasks, curated datasets, and rigorous assessment metrics. The task set encompassed five distinct problems including fluorescence landscape prediction and stability landscape prediction and spanning three major aspects of protein analysis: protein structure prediction, remote protein homolog detection, and protein design. This systematic approach facilitated the rigorous evaluation and comparison of different methodologies and models within the field of protein transfer learning. In 2022, PromptProtein [79] (taking protein sequences as inputs) stands as the pioneering prompt-based pre-trained protein model, aiming to address various levels of protein structures through prompt-guided multi-task pre-training. Furthermore, it incorporates a prompt fine-tuning module, enabling downstream tasks to effectively leverage specific levels of structural information as required. Through extensive experimentation in the domains of function prediction and biophysical properties prediction, PromptProtein demonstrates significant superiority over existing methods, showcasing substantial performance gains.

#### 3.3.7 Protein language models for protein-protein interaction and binding affinity prediction

Protein-protein interaction (PPI) constitutes a fundamental molecular-level process in biological activities, and its prediction holds profound significance in the realm of drug discovery and design. Investigating the interaction between proteins can help us discover new drug targets and design drugs with high efficiency and selectivity. PPLMs can help us efficiently and relatively accurately obtain protein-protein interaction types and binding affinities between proteins [80, 81].

The underlying motivation behind the KeAP [82] model aligns with that of ProtST, as both aim to incorporate more fine-grained information compared to OntoProtein [83]. KeAP adopts a triplet format consisting of (Protein, Relation, Attribute) as input, which is subsequently processed by distinct encoders and a specially designed cascaded decoder based on the Transformer architecture. The model employs Masked Language Modeling (MLM) as the primary pre-training task, facilitating efficient training. Leveraging its unique cross-attention fusion mechanism, the model excels in capturing intricate protein information at a finer granularity. As a result, KeAP exhibits exceptional performance across nine diverse downstream tasks including protein-protein interaction identification and protein-protein binding affinity estimation.

#### 3.3.8 Antibody large language models for antigen-receptor binding and antigen-antibody binding prediction

Antigen proteins break down in the cytoplasm, forming neoantigen peptides. These peptides bind to the Major Histocompatibility Complex (MHC), creating pMHC complexes. After a series of steps, these complexes reach the cell membrane for presentation. Subsequently, the T-cell Receptor (TCR) recognizes the pMHC complex, stimulating B cells to produce antibodies, triggering an immune response [84].

The application of language models in this process aims at accurately predicting the binding of peptides to HLA molecules as a key objective[85, 86]. Peptides serve as a life’s language, with large language models excelling in extracting context, particularly in pMHC binding and presentation prediction. For instance, MHCRoBERTa [87] utilizes the pretrained BERT to model the input amino acid sequences. Through effectively learning the biological meanings of each token, the MHCRoBERTa model is able to distinguish between different alleles. However, MHCRoBERTa primarily focused on pMHC-I prediction. BERTMHC [88] is a specific pMHC-II binding predicting method that incorporated 2,413 MHC–peptide pairs, encompassing 47 MHC class II alleles, into the training data. This effectively fills a gap in the field of pMHC-II binding prediction.

Another key goal is predicting the binding specificity of adaptive immune receptors (AIRs) for antigens. This variability in specificity mainly arises from the flexibility of three complementarity-determining region (CDR) loops (CDR1–3), with CDR3 being crucial for binding to antigenic peptides[89]. TCR-BERT [90] leverages unlabeled TCR CDR3 sequences to learn a general representation of TCR sequences. This enables downstream applications for predicting the antigen specificity in TCR recognition. However, TCR-BERT trains each individual AIR chain separately and fails to understand the paired interaction between the two chains of AIR. Jianhua Yao et al., effectively addressed this issue by pre-training a specially designed BERT model, SC-AIR-BERT [91], which outperforms other state-of-the-art methods in both TCR and BCR antigen-binding specificity prediction tasks.

After the antigen recognition process by T cells concludes, it stimulates B cells to produce specific antibodies that bind to the corresponding antigen[92]. In antibody language models section, three recent studies from PALMs will be introduced. PALMs are presented independently mainly because of the differences between their downstream tasks and those of PPLMs. It is also worth mentioning that the research on the large language model of antibodies is also a hot topic of recent research.

AbLang [93], an antibody language model built upon RoBERTa [94], was developed with the hypothesis that models trained on antibody databases would exhibit superior performance in addressing antibody-related challenges. One of the specific problems AbLang aims to solve is the restoration of residues that are lost during the sequencing process due to errors. Comparative evaluations have demonstrated that AbLang outperforms both IMGT germlines [95] and the general protein language model ESM-1b [76] in terms of accurately restoring the missing residues in antibody sequences. Furthermore, AbLang achieves this with increased efficiency, demonstrating a higher processing speed compared to the aforementioned models.

AntiBERTa [96] leverages the latent vectors derived from protein sequences, the model exhibits a discernible comprehension of the antibody “language” to a certain degree, as evidenced by the visual representations generated. The model’s ability is exemplified through a diverse array of tasks, including tracing the B cell origin of the antibody, quantifying immunogenicity, and predicting the antibody’s binding site.

The EATLM [97] is a straightforward architecture composed of a series of stacked transformer layers. Its uniqueness lies in the pre-training tasks it employs. Alongside the conventional Masked Language Modeling (MLM), the model introduces additional pre-training tasks, namely Ancestor Germline Prediction (AGP) and Mutation Position Prediction (MPP). These tasks aim to incorporate specific biological mechanisms into the pre-training phase. The most important contribution of the paper that proposed this model was to propose a reliable antibody-specific benchmark to evaluate different pre-protein language models and antibody language models.

### 3.4 Applications of large language models in drug discovery

**Figure 6.**
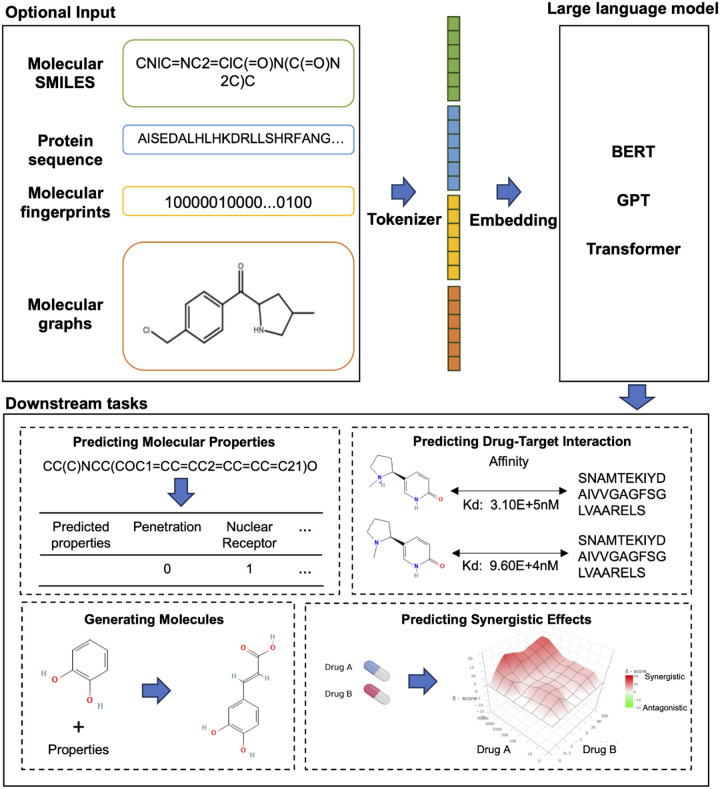
Applications of large language models in drug discovery. The language models for drug discovery take molecular SMILES, protein sequence, molecular fingerprints and molecular graphs as input, use transformer, BERT, GPT models to solve multiple biological tasks, including predicting molecular properties, predicting drug-target interaction, generating molecules and predicting synergistic effects.

Drug discovery is an expensive and long-term process that exhibits a low success rate. During the early stages of drug discovery, computer-aided drug discovery, employing empirical or expert knowledge algorithms, machine learning algorithms, and deep learning algorithms, serve to accelerate the generation and screening of drug molecules and their lead compounds [98–100]. It speeds up the entire drug discovery process, especially the development of small molecule drugs. Among commonly used medications, small molecule drugs can account for up to 98% of the total [101]. The structure of small molecule drugs exhibits excellent spatial dispersibility, and their chemical properties determine their good drug-like properties and pharmacokinetic properties [102]. With the development of deep learning and the proposal of large language models, it has become easy to apply these methods to discover hidden patterns of molecules and interactions between molecules for drugs (such as small molecules) and targets (such as proteins and RNA) that can be easily represented as sequence data. The Simplified Molecular-Input Line-Entry System (SMILES) string and chemical fingerprint are commonly used to represent molecules. Additionally, through the pooling process of graph neural networks(GNN), small molecules can be transformed into sequential representations [103]. With the protein sequence, large language models can engage in drug discovery through various inputs. Within this section, key tasks within the early drug discovery process that have effectively leveraged large language models will be introduced (Figure 6, Table 3, **Supplementary Table 3**).

#### 3.4.1 Large language models for drug-like molecular properties prediction

During the drug discovery process, significant attention is devoted to specific properties associated with candidate molecules, such as Absorption, Distribution, Metabolism, Excretion and Toxicology (ADMET) and Pharmacokinetics (PK). The objective is to facilitate the development of more efficacious, accessible, and safe drugs [104, 105]. The utilization of large language models in molecular property prediction encompasses downstream tasks that involve predicting these properties. Since the input of molecular SMILES representation is consistent, it is easy to improve and fine-tune the model based on specific datasets according to the requirements (properties of interest to researchers).

In contrast to its predecessors, SMILES-BERT [106] departed from the usage of knowledge-based molecular fingerprints as input. Instead, it adopted a representation method where molecules were encoded as SMILES sequences and employed as input for both pre-training and fine-tuning within a BERT-based model. This novel approach yielded superior outcomes across various downstream molecular property prediction tasks, surpassing the performance of previous models reliant on molecular fingerprints. ChemBERTa [107] is also a network based on BERT, which is essentially not much different from previous methods, and even not the most advanced in terms of performance at that time. However, it emphasizes the scalability of models based on large language models, and explores the impact of pre-training dataset size, tokenizer, and string representation, which provides several directions for discussions on molecular property prediction methods based on large language models. K-BERT [108], similarly based on the BERT architecture, distinguishes itself through the adoption of three distinct pre-training tasks in its pre-training phase: atom feature prediction, molecular feature prediction, and contrastive learning. This unique approach empowers the model to transcend the mere discovery of the SMILES paradigm and “comprehend” the underlying essence of SMILES representations. As a result, K-BERT exhibits remarkable performance across 15 drug datasets, showcasing its competence in the field of drug discovery. It is worth noting that with the growth of data, more and more molecules can be easily represented as graphs and processed through graph neural networks. Given the importance of graph neural networks in the development of molecular pre-training models, A BERT-based molecular pre-training network will be briefly introduced to demonstrate the use of large language model variants. The design of pre-training tasks greatly affects the performance of large language models. Drawing inspiration from the MLM task in BERT, Mole-BERT [109], a BERT-based graph-based pre-training neural network, introduces atom-level Masked Atoms Modeling (MAM) task and graph-level Triplet Masked Contrastive Learning (TMCL) task. These tasks enable the network to acquire a comprehensive understanding of the “language” embedded within molecular graphs. By adopting this approach, the network achieves exceptional performance across eight downstream data task datasets.

#### 3.4.2 Large language models for drug-like molecules generation

It is very difficult to chase the full coverage of the enormous drug-like chemical space (estimated at more than 10^63^ compounds), and traditional virtual screening libraries usually contain less than 10^7^ compounds and are sometimes not available. In such circumstances, the utilization of deep learning methods to generate molecules exhibiting drug-like properties emerges as a viable approach [110, 111]. Inspired by the generative pre-training model GPT, MolGPT [112] model was introduced. In addition to performing the next token prediction task, MolGPT incorporates an extra training task for conditional prediction, facilitating the capability of conditional generation. Beyond its capacity to generate innovative and efficacious molecules, the model has demonstrated an enhanced ability to capture the statistical characteristics within the dataset.

#### 3.4.3 Large language models for drug-target interaction predictions

The investigation of Drug-Target Interaction (DTI) holds paramount significance in the realm of drug development and the optimization of drug therapy. By attaining a profound comprehension of the interaction between drugs and their target proteins, it offers valuable guidance for the design and development of pharmaceutical agents. This expedites the drug development process and mitigates the expenditure of time and resources entailed in laboratory experimentation and trial-and-error methodologies [113, 114].

During the exploration of DTI, diligent focus is placed on the prediction of drug-target binding affinity. A proficiently trained DTI language model possesses the capability to conduct high-throughput drug screening, thereby expediting the drug discovery process. DTI-BERT employs a fine-tuned ProtBERT [115] model to process protein sequences, while employing discrete wavelet transform for the processing of molecular fingerprints of drug molecules. Subsequently, by acquiring the hidden states of the corresponding pairs, the ultimate outcome is achieved through concatenation and subsequent neural network processing. This approach is simple and effective. TransDTI [116] is a multi-class classification and regression workflow. In contrast to DTI-BERT, this model not only uses fine-tuned SMILES-BERT to extract drug features, but also expands the selection of fine-tuned large protein models. After acquiring potential representations of drug-target pairs, the authors subject the representations to downstream neural networks for the completion of a multi-classification task. Additionally, the paper employs molecular docking and dynamic analysis as means of verifying the model’s predictions. Hyeunseok Kang et al. did similar work using different pre-trained models in 2022 [117]. The Chemical-Chemical Protein-Protein Transferred DTA (C2P2) method uses pre-trained protein and molecular large language models to capture the interaction information within molecules, similar to previous methods. Given the relatively limited scale of the DTI dataset, C2P2 leverages protein-protein interaction (PPI) and chemical-chemical interaction (CCI) tasks to acquire knowledge of intermolecular interactions and subsequently transfer this knowledge to affinity prediction tasks. The incorporation of this training framework undeniably enhances the network’s ability to predict the binding affinity between the two molecules, as evidenced by the experimental outcomes [118].

It is worth highlighting that in scenarios involving the docking or when emphasizing the spatial structure of a complex, methodologies incorporating 3D convolution networks, point clouds-based networks, and graph networks are often employed [119–122]. Although these methods can better capture the inter-molecular interactions, they inevitably consume more computing resources. In situations where the molecular structure is unknown, but the sequence is available, the prediction of DTI using large-scale models still holds significant promise.

#### 3.4.4 Large language models for drug synergistic effects predictions

Combination therapy is common for complex diseases like cancer, infections, and neurological disorders, often surpassing single-drug treatments. Predicting drug pair synergy, where combining drugs boosts therapeutic effects, is vital in drug development. However, it’s challenging due to many drug combinations and complex biology [123, 124].

Various computational methods, including machine learning, help predict drug pair synergy. Wei Zhang et al. [125] introduced DCE-DForest, a model for predicting drug combination synergies. It uses a pretrained drug BERT model to encode the drug SMILES and then predicts synergistic effects based on the embedding vectors of drugs and cell lines using the deep forest method. Mengdie Xua et al. [126] utilized a fine-tuned pre-trained large language model and a dual feature fusion mechanism to predict synergistic drug combinations. Its input includes hashed atom pair molecular fingerprints of drugs, SMILES string encodings, and cell line gene expressions. They conducted ablation analyses on the dual feature fusion network for drug-drug synergy prediction, highlighting the significant role of fingerprint inputs in ensuring high-quality drug synergy predictions.

### 3.5 Applications of large language models in single cell analysis

**Figure 7.**
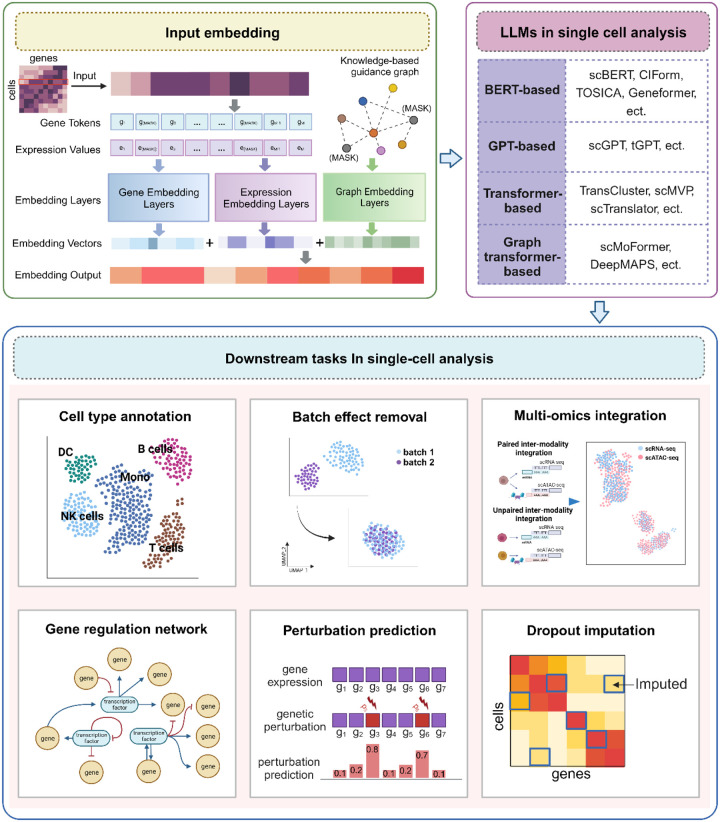
Applications of large language models in single cell analysis. The single cell language models take gene expression or single cell multi-omics data as input, use transformer, BERT, GPT models to solve multiple biological tasks, including cell type annotation, batch effect removal, multi-omics integration, gene regulation network inference perturbation prediction, dropout imputation.

The emergence of single-cell RNA sequencing (scRNA-seq) has marked the onset of a revolutionary era in genomics and biomedical research. Unlike traditional bulk RNA sequencing methods, scRNA-seq allows us to delve into the intricacies of gene expression at single-cell resolution, offering unprecedented insights and paving the way for numerous groundbreaking advancements [127–130]. One of the most significant changes brought about by scRNA-seq is its ability to uncover cellular heterogeneity within tissues and organisms. It facilitates the identification and recognition of diverse cell types, subpopulations, and rare cell states that were previously concealed in bulk measurements. As we discussed in the previous sections, large language models have found successful applications in the domains of genomics, transcriptomics, proteomics and drug discovery. In this section, our attention turns to single-cell language models that can be employed for various downstream tasks in single-cell analysis. These tasks include identifying cell types and states, discovering novel cell populations, inferring gene regulation networks, and integrating single-cell multi-omics (scMulti-omics) data, among others. (Figure 7, Table 4, **Supplementary Table 4**).

#### 3.5.1 Single-cell language models for cell clustering based on scRNA-seq data

Cell clustering for single-cell RNA sequencing (scRNA-seq) is crucial steps in deciphering the complex landscape of cellular heterogeneity within biological samples [131–136]. Single-cell clustering aims to group individual cells into clusters according to their gene expression profiles. Traditional single cell clustering methods mainly use clustering algorithms such as hierarchical clustering, k-means algorithm, neural network-based methods, etc. to divide cells into clusters. Large language models enable cell clustering on large amount of scRNA-seq data from different tissues, species, organs, sequencing technologies, platforms, etc. For example, tGPT [137] is a generative pretraining model from transcriptomes that requires input genes ranked in descending order of their expression. tGPT can be trained to learn the feature representation of scRNA-seq based on high-expressed genes. The learned feature representation is applied to cell clustering on atlas-scale data including Human Cell Atlas (HCA) [138], Human Cell Landscape (HCL) [139], Tabula Muris [136] and Macaque Retina [140] datasets using Leiden algorithm [141]. scFoundation [142] is the currently largest large language model in single-cell field, featuring 100 million parameters across 50 million gene expression profiles. scFoundation introduces a novel pre-training task known as read-depth-aware (RDA) modeling based on Bayesian down sampling, which selects genes of a low read-depth variant in the same cell instead of directly using neighbor genes to predict masked genes. scFoundation has a transformer-based encoder-decoder structure. Only non-zero and non-masked gene were fed into the encoder to learn cell embedding for cell clustering.

#### 3.5.2 Single-cell language models for cell type annotation based on scRNA-seq data

Single-cell annotation focuses on assigning biological labels, typically cell type or cell state, to individual cells or clusters. It plays a pivotal role in understanding cell function, identifying disease-specific cell populations, and unraveling the intricacies of tissue development and homeostasis. However, annotating cell types in single-cell RNA sequencing data is very challenging due to the high levels of noise, dropout events, and batch effects inherent in scRNA-seq data. The remarkable success of large language models in natural language processing and computer vision opens new avenues for addressing cell type annotation in single-cell RNA sequencing data. Currently, there have been emerging some computational tools utilizing large language models for cell type annotation using scRNA-seq data, such as CIForm [143], TOSICA [144], scTransSort [145], TransCluster [146], scBERT [147] and scGPT [148]. CIForm [143] is designed for cell type annotation on large-scale datasets with multiple reference data based on transformer. After a transformer encoder to learn the cell embeddings, a multi-layer neural network-based classifier is employed in CIForm to predict the cell types of single cells. CIForm was evaluated on both intra-datasets and inter-datasets, considering the annotation on different species, organs, tissues and technologies, reference and query data from different sequencing platforms or studies (which we refer to batch size effect in single cell analysis), and even multi reference data from different sources. TOSICA [144] developed an interpretable cell type annotation method that converts gene tokens into pathway/regulons tokens by adding a knowledge-based mask matrix from GSEA to the fully connected weight matrix in the gene embedding step. Only highly variable genes are used as input in TOSICA and the class token (CLS) in the output of the transformer is utilized to predict the cell type probabilities using the whole conjunction neural network cell type classifier. In addition to interpretable cell type annotation, TOSICA can also discover new cell types, perform interpretable trajectory analysis and be immune to batch effects in scRNA-seq data. scTransSort [145] and TransCluster [146] were designed by the same author team. scTransSort [145] proposed a gene patch embedding that uses CNN to generate a sequence of flattened 2D gene embedding patches to alleviate the problem of scRNA-seq data sparsity and avoid using HVGs. Positional embedding representing the relative positions between genes is added to each patch, and then passed through a transformer consisting of a multi-head self-attention mechanism and a fully connected feedforward to obtain the learned cell embedding, and then a linear classifier is used for supervised cell type classification training. The transformer structure in TransCluster [146] included both self-attention-based encoder and decoder, combined with a one-dimensional CNN to extract features from input. The linear classifier is the final step to conduct cell type annotation. scBERT [147] acquires a broad understanding of the syntax of gene-gene interactions during the pre-training phase, aiming to eliminate batch effects across datasets and enhance generalizability. In the fine-tuning step, model’s parameters guided by reference datasets are retained since a classifier is added to the pre-trained performer. The excellent design of scBERT lies in giving up the utilization of HVGs and dimensionality reduction. Instead, scBERT replaces the transformer encoder employed in BERT with Performer [149] to enhance the model’s scalability. Consequently, scBERT enables the unbiased, data-driven discovery of gene expression patterns and longer-range dependencies for cell type annotation. scGPT [148] was developed based on a generative pre-trained foundation model to learn cell and gene representations from a variety of single-cell data after HVG selection. In the pre-training step, scGPT employs stacked transformer layers with multi-head attention, enabling the simultaneous learning of cell and gene embeddings. scGPT was trained in an autoregressive manner via zero-shot learning, initially generating gene expression values from cell embeddings, and then gradually learning to generate gene expression of cells by leveraging existing knowledge of gene expressions. During the fine-tuning step. scGPT used a supervised model to annotate unknown cells from their cell representation by introducing a multilayer perceptron-based (MLP-based) classifier to the generative pre-trained foundation model. Notably, scGPT employed special tokens, batch tokens and modality tokens, to eliminate batch effects and modality differences in the pre-training single-cell reference datasets to improve the annotation accuracy.

#### 3.5.3 Single-cell language models for gene function analysis based on scRNA-seq data

In addition to cell-level tasks, such as cell clustering and cell type annotation, the attention mechanism in the transformer can learn the relationship between genes, and the transformer can output the learned gene embedding after pre-training and finetuning, which can be used for gene function analysis for scRNA-seq data. As mentioned in section 3.5.2, scGPT [148] is a generalizable feature extractor based on zero-shot learning that enables scGPT to be applied to gene expression prediction and genetic perturbation prediction. The attention matrix learned by scGPT is used to infer gene regulation network. Similar to scGPT, scFoundation [142] is a foundation model that can learn both cell representation and gene representation. Zero-expressed genes and masked genes are combined with the the output from the transformer-based encoder. This combined information is then input into the decoder and projected to gene expression values through a multilayer perceptron (MLP). The gene context expression is employed to formulate a cell-specific gene graph, facilitating the prediction of perturbations using the GEARS [150] model. It is worth mentioning that another large language model Geneformer [151] is pre-trained on a vast scale of single-cell transcriptomes. All genes of each cell are reordered according to their gene expression and input into the transformer for training. Subsequently, it undergoes fine-tuning for diverse downstream tasks, encompassing the prediction of dosage-sensitive disease genes and downstream targets, forecasting chromatin dynamics, and anticipating network dynamics. This fine-tuning process leverages the pre-trained weights transferred to the task-specific models with limited data.

#### 3.5.4 Single-cell language models for single-cell multi-omics data

Studying single-cell multi-omics data involves integrating information from different omics technologies (e.g., genomics, transcriptomics, epigenomics and proteomics) at the single-cell level, which has multiple advantages compared to studying single-omics data types. The adaptability, generalization capabilities, and feature extraction abilities of large language models make them valuable tools to find solutions for feature-variance, sparsity and cell heterogeneity that scMuti-omics data suffers from. A crucial phase in the analysis of single-cell multi-omics data involves the integration of such diverse datasets. scGPT [148] utilizes supplementary sets of tokens to signify distinct sequencing modalities in the context of scMulti-omics integration tasks. The modality tokens are associated with input features, such as genes, regions, and proteins. They are added to the transformer output, either at the feature or cell level, before proceeding with specific fine-tuning objectives. This deliberate inclusion helps prevent the transformer from amplifying attention within features of the same modalities, while simultaneously downplaying the significance of those associated with different modalities. scMVP [152] is designed specific for integration of paired single-cell RNA-seq and ATAC-seq data, where gene expression and chromatin accessibility are in the same cell [153–156]. scMVP uses mask attention-based scRNA encoders and transformer multi-head self-attention-based scATAC encoders to project scRNA-seq and scATAC-seq into latent space. The distribution of latent embedding representing the joint profiling of scRNA and scATAC is the GMM distribution. A cell type-guided attention module calculates correlations between scRNA and scATAC in the same cell. Subsequently, scRNA-seq and scATAC-seq data are reconstructed and imputed by learning parameters of the negative binomial (NB) and zero-inflated Poisson (ZIP) distributions using a two-channel decoder network with a structure similar to the encoder network. DeepMAPS [157] is a graph transformer-based method, but it is designed for data integration and inference of biological networks from scMulti-omics data, encompassing scRNA-seq, scATAC-seq, and CITE-seq data. The graph constructed by DeepMAPS consists of nodes for genes and cells, so all other modalities should map their features to genes. The transformer in DeepMAPS aims to learn both local and global features to build cell-cell and gene-gene relations combined with RNA velocity. The cell-cell relation can further be used to infer cell-cell communications.

In recent years, advancements in technology have enabled the concurrent characterization of different modalities in the same cell. This progress has given rise to computational tools capable of predicting one modality from another. One such tool is scTranslator [158], which translates single-cell transcriptome to proteome. scTranslator is pre-trained on both paired bulk data and paired single-cell data, then it is fine-tuned to infer protein abundance from scRNA-seq data by minimizing the mean squared error (MSE) loss between predicted and actual proteins. The learned attention matrix is applied to infer integrative gene-gene, protein-protein, and gene-protein regulatory. Another method called scMoFormer [159] can not only translate gene expression to protein abundance, but is also applicable to multi-omics predictions, including protein abundance to gene expression, chromatin accessibility to gene expression, gene expression to chromatin accessibility using graph transformers. Taking protein prediction task as an example, scMoFormer constructs cell-gene graph, gene-gene graph, protein-protein graph, and gene-protein graph based on gene expression profiles and prior knowledge from STRING database [160]. Each modality has a separate transformer to learn the global information that may not be included in prior knowledge. Message-passing graph neural networks (GNNs) link nodes across various graphs, while transformers are employed to precisely map gene expression to protein abundance.

## 4. Conclusion

Pre-trained large language models have been used in multiple biological tasks. In this review, we discuss the applications of LLMs in genomics, transcriptomics, proteomics, single-cell analysis, and drug discovery. As discussed above, LLMs can learn the DNA and RNA sequencing pattern to predict DNA and RNA-based modification and regulation. LLMs can be pre-trained using proteins to achieve protein structure prediction, protein generation, protein function annotation, and protein interaction prediction. LLMs can learn cell and gene embeddings from scRNA-seq and scMulti-omics data to annotate cell types, integrate datasets and predict gene-related functional analysis. LLMs can be trained to predict molecular properties or generate molecules based on molecular scaffolds and specified molecular properties, predict interactions between drugs and targets as well as drug synergies.

In genomics and transcriptomics, large language models have been used in plenty of biological tasks. Currently, DNA and RNA sequences are recognized as similar languages, existing works have largely hinged on k-mer, fixed-length permutations of A, G, C, and T/U, as the token of the genome language due to its simplicity. LLMs were asked to learn the complex statistical properties of existing biological systems, the pre-trained foundational LLM models have made significant strides in this area for effectively addressing diverse categories of downstream tasks. DNABERT is a DNA pre-trained on DNA languages, however, some RNA languages use DNABERT instead of RNA foundation models to train the RNA sequences for specific biological tasks. For example, M6A-BERT-Stacking serves as a tissue-specific predictor designed to identify RNA N6-methyladenosine sites, utilizing DNABERT and a Stacking Strategy. In its approach, M6A-BERT-Stacking utilizes pre-trained DNABERT and fine-tuned DNABERT attention models. Notably, it has been observed that DNABERT effectively directs attention to critical regions within known m6A sites and captures informative feature representations from input sequences. What’s more, some biological tasks can be predicted from DNA or RNA sequences. Like DNABERT and DNABERT-2 can predict the splice site from the reference genome, however, SpliceBERT was trained in the RNA sequence of RNA-seq and predicted the splice site of specific pre-mRNA. Since DNA and RNA sequences both consist of four letters, their foundational sequences language model might be able to be used in both DNA and RNA-related tasks. In summary, today’s LLMs are sufficiently advanced to model molecular biology. However, we expect it to learn one-step causality relationships which can be learned from the correlations across modalities such as DNA variation, and mRNA abundance.

In proteomics, the protein language models take sequence information as input and produce fine-tuned output results tailored to different downstream tasks associated with proteins. In terms of the model’s parameter magnitude, the large parameter quantity of the language model establishes a solid foundation for effectively addressing diverse categories of downstream tasks. Nonetheless, the excessive parameters often pose deployment challenges for ordinary researchers. One approach involves utilizing large models online, akin to our daily use of GPT, albeit at the expense of additional deployment costs. Another method entails employing knowledge distillation or other algorithms to capture the refined knowledge that researchers deem valuable from the large model. Considering the model’s input perspective, the most basic protein language model is limited to handling inputs such as MSA and protein sequences. However, incorporating other modalities of information, such as 3D structural information of proteins, introduces challenges in training and utilizing large language models. One approach involves transforming the additional modalities of information into a sequence-based format, necessitating careful consideration of the effectiveness and rationality of such information conversion across modalities. Another method involves integrating other large models to collectively capture the multi-modal information pertaining to proteins. This approach requires consideration of multi-modal fusion techniques, fusion timing, and related concerns. The increase of diverse types of protein-related data has driven the protein language models to evolve from an independent model to an integral component of larger protein models.

In computer-aided drug discovery, the key steps include processes such as docking, scoring, and screening. When employing large language models, it becomes feasible to solely consider the sequence information of molecules, leading to heightened prediction efficiency. However, the absence of spatial structural information significantly impacts prediction accuracy. Therefore, the use of deep learning for drug discovery is more based on structure, which means that the sequence features extracted by large language models are usually input as prior knowledge into the network. Drawing inspiration from the construction of other large language models in the field of bioinformatics, it is also plausible to construct large models based on molecular structure, such as large graph models, to predict binding affinity, based on the CrossDocked2020 dataset. Additionally, the experience gained from the prediction of PPI and CCI can be transferred to the prediction of DTI using techniques like transfer learning. Computer-aided drug discovery also involves molecular generation. The generation of drug molecules needs to consider properties such as effectiveness, novelty, and drug-likeness. While existing methods do incorporate these properties to some extent, there remains a dearth of extensive research on the generated molecules, as well as a scarcity of practical chemical or biological experimental verification to support their viability.

In single-cell analysis, large language models are pre-trained on a large scale of gene expression to apply to cell-level and gene-level downstream tasks. There are many differences from NLP in single-cell applications of LLMs. First, scRNA-seq data suffers from high sparsity, which is also one of the difficulties to be solved in large language models applied to single-cell RNA-seq data. CIForm, TOSICA, TransCluster and scGPT select highly variable genes as input to alleviate the problem of scRNA-seq data sparsity and reduce the training burden of large language models. Second, gene expression differs from human language and sequence data, it is continuous values and does not have orders between gene tokens. Thus, how to define the genes’ position is a critical problem of LLMs in single-cell analysis. tGPT and Geneformer sort genes from high to low according to their gene expression. CIForm and TransCluster apply sine and cosine functions to determine the position information. scBERT uses gene2vec to transfer gene expression to discrete values. Third, batch effects can pose a significant challenge in cell type clustering, annotation, and integration, especially when the input comprises multiple datasets from distinct sequencing batches or technologies. The generalizability of large language models can adapt to different biological datasets and applications, making them versatile tools to be applied to various single-cell datasets. It is worth mentioning that the combination of Graph Neural Networks (GNNs) and transformers has brought about significant advancements and meaningful contributions to the field of single-cell analysis, such as scMoFormer uses graph transformers to construct cell-gene graph, gene-gene graph, protein-protein graph, and gene-protein graph for multi-omics predictions. DeepMAPS constructs a graph of cells and genes and uses graph transformer to estimate the importance of genes to cells. The combination of GNNs and Transformers allows for a more comprehensive representation of the intricate relationships and dependencies present in single-cell data. GNNs excel at capturing local interactions within cellular neighborhoods, while Transformers effectively capture long-range dependencies. This synergy enables a holistic understanding of the cellular landscape, leading to improved feature learning. In summary, large language models excel at extracting meaningful features from raw data in single-cell analysis. They can learn representations of gene expression patterns, cell types, and other relevant information from the data, even without prior domain-specific knowledge.

In conclusion, today’s large language models have reached a level of sophistication that enables them to effectively model the intricacies of molecular biology. With continuous advancements in single-cell technologies and the expanding landscape of omics sciences, including proteomics, metabolomics, lipidomics, and other-omic assays, there is a growing capacity for conducting increasingly detailed and efficient measurements. These developments contribute to our ability to unravel the complexities inherent in the diverse molecular layers spanning from DNA to the intricacies of human physiology. As we delve deeper into the realms of these cutting-edge technologies, we unlock new insights that pave the way for a more comprehensive understanding of the dynamic interplay within the molecular landscape.

## Figures and Tables

**Table 1.** Large language models for genomic and transcriptomic tasks

Input data	Biological tasks	Models
DNA sequence	Genome-wide variant effects prediction	DNABERT, DNABERT-2, GPN, Nucleotide Transformer
	DNA cis-regulatory regions prediction	DNABERT, DNABERT-2, BERT-Promoter, iEnhancer-BERT, Nucleotide Transformer
	DNA-protein interaction prediction	DNABERT, DNABERT-2, TFBert, GROVER, and MoDNA
	DNA methylation (6mA,4mC 5hmC) prediction	BERT6mA, iDNA-ABF, iDNA-ABT, and MuLan-Methyl
	RNA splice sites prediction from DNA sequence	DNABERT, DNABERT-2
RNA sequence	RNA 2D/3D structure prediction	RNA-FM, RNA-MSM, and RNA-FM
	RNA structural alignment, RNA family clustering	RNABERT
	RNA splice sites prediction from RNA sequence	SpliceBERT
	RNA N7-Methylguanosine modification prediction	BERT-m7G
	RNA 2’-O-methylation Modifications prediction	Bert2Ome
	Multiple types of RNA modifications prediction	Rm-LR
	Predicting the association between miRNA, lncRNA and disease	BertNDA
	Identifying lncRNAs	LncCat
	lncRNAs’ coding potential prediction	LSCPP-BERT
	Protein expression and mRNA degradation prediction	CodonBERT

**Table 2.** Large language models for proteomic tasks.

Input data	Downstream tasks	Models
	Secondary structure and contact prediction	MSA Transformer, ProtTrans, SPRoBERTa, TAPE, KeAP
	Protein sequence generation	ProGen, ProtGPT2
Protein sequences	Protein function prediction	SPRoBERTa, ProtST, PromptProtein
MSAs	Major PTMs prediction	ProteinBERT
Gene ontology annotations	Evolution and mutation prediction	SPRoBERTa, UniRep, ESM-1b, TAPE
Triplets of protein-relation-attribute	Biophysical properties prediction	TAPE, PromptProtein
Protein property descriptions	Protein-protein interaction and binding affinity prediction	KeAP
	Antigen-Recepter binding prediction	MHCRoBERTa, BERTMHC, TCR-BERT, SC-AIR-BERT
	Antigen-Antibody binding prediction	AbLang, AntiBERTa, EATLM

* The input of models is one or several types of protein language data.

**Table 3.** Large language models for drug discovery tasks.

Input data	Downstream tasks	Models
Molecular SMILES	Predicting Molecular Properties	SMILES-BERT, ChemBERTa, K-BERT
	Generating Molecules	MolGPT
Molecular graphs	Predicting Molecular Properties	MOLE-BERT
Molecular fingerprints and protein sequences	Predicting Drug-Target Interaction	DTI-BERT
Molecular SMILES and protein sequences	Predicting Synergistic Effects	TransDTI, C2P2, Hyeunseok Kang et al.

**Table 4.** Large language models for single cell tasks.

Input data	Downstream tasks	Models
scRNA-seq data	Cell clustering	tGPT, scFoundation
	Cell type annotation	CIForm, TOSICA, scTransSort, TransCluster, scBERT, scGPT
	Gene function analyses (Gene expression prediction, gene network inference, gene perturbation prediction, discovery of key network regulators, and identifying candidate therapeutic targets)	scGPT, scFounfation, Geneformer
scMuti-omics data	Single-cell multi-omics integration	scGPT, scMVP, DeepMAPS
	Biological network inference	DeepMAPS
	Cell-cell communications	
	Data imputation	scMVP
	Translating gene expression to protein abundance	scTranslator, scMoFormer
	Single-cell multimodal prediction	scMoFormer
	Integrative regulatory inference	scTranslator
