# Advancing bioinformatics with large language models: components, applications and perspectives

**Published:** 2025-01-31

**Authors:** Jiajia Liu, Mengyuan Yang, Yankai Yu, Haixia Xu, Tiangang Wang, Kang Li, Xiaobo Zhou

**Affiliations:** 1 Center for Computational Systems Medicine, McWilliams School of Biomedical Informatics, The University of Texas Health Science Center at Houston, Houston, Texas, 77030, USA; 2 Department of Cell Biology and Genetics, School of Basic Medical Sciences, Xi’an Jiaotong University Health Science Center, Xi’an, China; 3 School of Computing and Artificial Intelligence, Southwest Jiaotong University, Chengdu, Sichuan 611756, China; 4 West China Biomedical Big Data Center, West China Hospital, Sichuan University, Chengdu, Sichuan 610041, China; 5 McGovern Medical School, The University of Texas Health Science Center at Houston, Houston, TX 77030, USA; 6 School of Dentistry, The University of Texas Health Science Center at Houston, Houston, TX 77030, USA

## Abstract

Large language models (LLMs) are a class of artificial intelligence models based on deep learning, which have great performance in various tasks, especially in natural language processing (NLP). Large language models typically consist of artificial neural networks with numerous parameters, trained on large amounts of unlabeled input using self-supervised or semi-supervised learning. However, their potential for solving bioinformatics problems may even exceed their proficiency in modeling human language. In this review, we will provide a comprehensive overview of the essential components of large language models (LLMs) in bioinformatics, spanning genomics, transcriptomics, proteomics, drug discovery, and single-cell analysis. Key aspects covered include tokenization methods for diverse data types, the architecture of transformer models, the core attention mechanism, and the pre-training processes underlying these models. Additionally, we will introduce currently available foundation models and highlight their downstream applications across various bioinformatics domains. Finally, drawing from our experience, we will offer practical guidance for both LLM users and developers, emphasizing strategies to optimize their use and foster further innovation in the field.

## 1. Introduction

Significant progress has been made in the field of natural language processing with the advent of large language models. Examples of these models include OpenAI’s GPT-X [1] and Google’s BERT [2] models. These models are transformative because they can understand, generate, and manipulate human language at an unprecedented scale. Vast Large language models are typically trained on datasets that encompass a significant portion of the internet’s text, enabling them to learn the complexities of language and context. These models are built upon a neural network architecture called transformers [3]. The transformer architecture revolutionized NLP due to its parallelization, scalability, and ability to capture long-range dependencies in text. Instead of relying on recurrent or convolutional layers, transformers use self-attention mechanisms, as previously described, which allow them to assess the importance of every word in a sentence when understanding context. This innovation is key to their remarkable performance.

**Figure 1.**
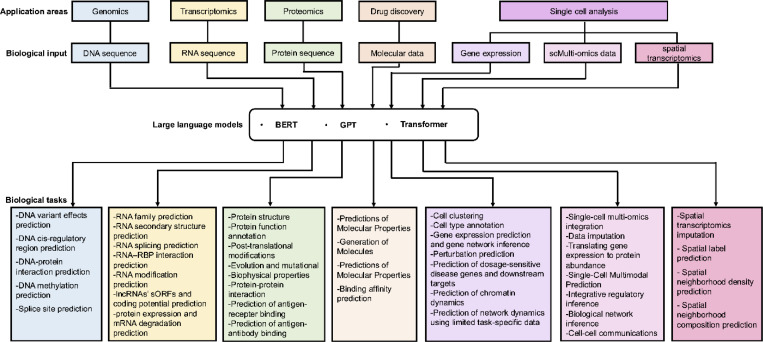
Summary of the application of large language models in bioinformatics in this review. Applications of large language models in bioinformatics include applications in genomics, transcriptomics, proteomics, drug discovery and single-cell analysis. Applications of LLMs in genomics focus on LLMs using DNA sequence; applications of LLMs in transcriptomics focus on using RNA sequence; applications of LLMs in proteomics focus on LLMs using protein sequence; applications of LLMs in drug discovery focus on LLMs using molecular data and applications of LLMs in single-cell analysis focus on LLMs using scRNA-seq, scMulti-omics and spatial transcriptomics data. Each corresponds to a variety of biological downstream tasks.

The training regimen for large language models comprises two phases: pre-training and fine-tuning. During pre-training, the model is trained on an extensive corpus of text data to acquire proficiency in grammar, factual knowledge, reasoning abilities, and word understanding. Fine-tuning tailors these models for specific tasks like translation, summarization, or question-answering. The adaptability of large language models is a major advantage; they can excel at various NLP tasks without task-specific architectures. However, they have found applications in diverse fields beyond NLP, including biology, healthcare, education, finance, customer service, and more. In particular, there have been many successful applications of large language models in the field of bioinformatics. In this manuscript, we focus on the applications of large language models to several bioinformatic tasks through five areas: DNA level, RNA level, protein level, drug discovery and single-cell analysis. Applications of LLMs in genomics focus on LLMs using DNA sequence; applications of LLMs focus on in transcriptomics using RNA sequence; applications of LLMs in proteomics focus on LLMs using protein sequence; applications of LLMs in drug discovery focus on LLMs using Molecular SMILES (seq) and applications of LLMs in single-cell analysis focus on LLMs using scRNA-seq, scMulti-omics and spatial transcriptomics data (Figure 1).

## 2. Understanding the Building Blocks of Large Language Models in Bioinformatics

Building large language models involves several critical components, including tokenization methods, embedding techniques, attention mechanisms, transformer architectures, and the training processes for large-scale models. Each of these elements plays a vital role in enabling the models to process, understand, and generate complex data.

### 2.1 Tokenization and input embedding

**Figure 2.**
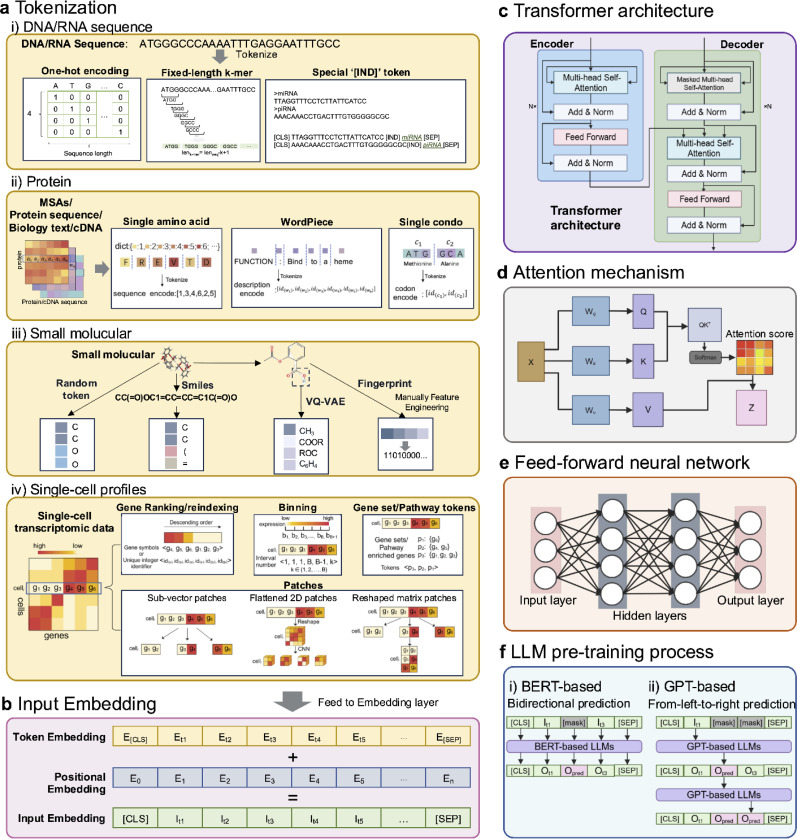
Building blocks of large language models in bioinformatics. **a,** tokenization methods tailored to various data types, including DNA/RNA sequences, proteins, small molecules, and single-cell data. **b,** input embedding strategies used in large language models to encode tokenized data. **c,** schematic representation of the transformer architecture, a foundational structure in LLMs. **d,** the attention mechanism, enabling models to focus on important features in sequences. **e,** the feed-forward network, a critical component of transformers for learning hierarchical representations. **f,** pre-training processes for BERT and GPT-based models, highlighting BERT’s bidirectional prediction approach and GPT’s left-to-right prediction strategy.

Tokenization methods are essential for processing raw input data, breaking it down into smaller, manageable units (tokens) that can be analyzed and processed by models. The choice of tokenization method varies depending on the type of data being handled (Figure 2a, Table 1).

In DNA and RNA sequence data, tokenization converts raw nucleotide sequences (A, T, C, G for DNA or A, U, C, G for RNA) into a numerical format suitable for computational models. A common method is one-hot encoding, where each nucleotide is represented as a binary vector with a ‘1’ indicating its position (e.g., [1, 0, 0, 0] for A in DNA), as used in RNA-FM [4] and RNA-MSM [5]. Another widely adopted approach is k-mer tokenization, which segments sequences into overlapping substrings of fixed length ‘k’ (e.g., for k=3, “ATGC” becomes “ATG” and “TGC”). This method is employed in models like DNABERT[6], DNAGPT [7], and RNABERT [8].

Additionally, specialized tokens such as ‘[IND]’ can be introduced to mark the start or end of sequences or to handle unknown characters or gaps, as demonstrated in RNAErnie [9].

In protein language models, the input data primarily includes multiple sequence alignments (MSAs), protein sequences, biomedical/biological text, and cDNA. The basic units of MSAs and protein sequences are amino acids, leading most protein language models to use Single Amino Acid Tokenization, where protein sequences are segmented into individual amino acids. This approach is akin to the k-mers method used for DNA and RNA sequences and is employed in models such as ESM-1b [10], ProtTrans [11], and ProGen [12]. For biomedical and biological text, including general descriptions, conditioning tags in generative models, and resources like Gene Ontology (GO), tokenization methods from natural language processing (NLP) are widely used. Methods like WordPiece Tokenization build vocabulary using frequency-based greedy algorithms and segment text into discrete tokens, as demonstrated in ProtST [13]. For cDNA data, tokenization is similar to that of protein sequences but differs in the basic unit. Instead of amino acids, sequences are tokenized into codons, or triplets of nucleotides, as seen in CaLM [14].

In drug discovery, small molecule drugs account for 98% of commonly used medications [1]. LLMs leverage four main tokenization methods to uncover molecular patterns and drug-target interactions. Atom-level tokenization treats molecules as sequences of individual atoms, analogous to character-level text representation, as seen in K-BERT [15]. MolGPT [16] utilizes a SMILES tokenizer that segments molecular structures into units such as atoms, bond types, and ring markers. A Graph-based VQ-VAE approach enhances this by encoding atoms into context-aware discrete values, distinguishing roles like aldehyde versus ester carbons, based on latent codes derived from a graph-based Vector Quantized Variational Autoencoder (VQ-VAE). This method categorizes atoms into chemically meaningful sub-classes, enriching the molecular vocabulary. Fingerprint tokens, another method, represent molecules through binary or numerical vectors summarizing molecular properties or structural patterns, as seen in SMILES-BERT [17].

Tokenization methods for single-cell profiles include four main strategies. Gene ranking/reindexing-based methods rank genes by expression levels and create tokens using ranked gene symbols or unique integer identifiers, as seen in Geneformer [18] and tGPT [19]. Binning-based methods divide gene expression into predefined intervals, assigning tokens based on the corresponding bin, used in models like scBERT [20] and scGPT [21]. Gene set or pathway-based methods group genes into biologically meaningful sets, such as pathways or Gene Ontology terms, with tokens representing the activation of these sets, exemplified by TOSICA [22]. Patch-based methods segment gene expression vectors into equal-sized sub-vectors, as seen in CIForm [152]. Alternatively, convolutional neural networks (CNNs) can be used to transform the reshaped gene expression matrix into several flattened 2D patches, as demonstrated by scTranSort [23]. Another variation involves reshaping the sub-vectors into a gene expression matrix after segmentation, as employed in scCLIP [24]. In addition to the four methods mentioned above, a more direct approach involves projecting gene expression directly, as seen in models like scFoundation [25], and scMulan [26]. Alternatively, some methods tokenize cells instead of genes, as exemplified by models such as CellPLM [27], ScRAT [28], and mcBERT [29], which utilize cell tokens during model training (Table 1). These strategies allow models to capture biological structure and variability, tailoring tokenization to single-cell data characteristics.

After tokenization, embedding converts tokens into continuous vector representations, capturing the semantic relationships between them. Positional encoding represents the token order by adding vectors that encode the relative or absolute positions of tokens in the sequence. The final step involves combining the token embeddings with the positional embeddings to create a unified input embedding, which is then fed into the model for further processing (Figure 2b).

### 2.2 Architecture of transformer models

Transformers are the foundational architecture in large language models (LLMs) and consist of two main components: the encoder and the decoder. The encoder takes the input data and processes it in parallel across multiple layers to capture relationships within the sequence. The decoder, on the other hand, generates output sequences based on the encoder’s processed information, typically used in tasks like translation or text generation. Each component is built on layers of multi-head attention, add and norm layer, and feed-forward layer (Figure 2c).

#### Attention Mechanism:

A key innovation of the transformer is the attention mechanism, particularly self-attention [3], which allows the model to weigh the importance of different tokens in a sequence relative to each other. In self-attention, each token computes a score based on how much attention it should pay to other tokens in the sequence. This is done by calculating three key components: Query (Q), Key (K), and Value (V) vectors for each token (Figure 2d). The attention score is computed as the dot product between the Query of one token and the Key of another token, followed by a softmax operation to normalize the scores. These scores are then used to weight the Value vectors, which are aggregated to form the output representation for each token as following [3]:

(1) $$Attention(Q,K,V)=softmax\left(\frac{QK^{T}}{\sqrt{d_{k}}}\right)V$$

**Multi-head attention** extends this idea by running multiple attention mechanisms (or “heads”) in parallel. Each attention head processes the input tokens in a slightly different way by using different sets of learned weights for the Q, K, and V vectors. The results of all heads are concatenated and linearly transformed, allowing the model to capture different aspects of relationships between tokens simultaneously. This mechanism enables the model to focus on various parts of the input sequence at once, learning different types of interactions between tokens. For example, in single-cell foundation models, self-attention can help identify important gene interactions by determining which genes (tokens) should focus on each other during processing. In this way, multi-head attention allows the model to capture complex relationships between genes in single-cell RNA-seq data, where multiple aspects of gene expression (such as co-expression patterns or functional relationships) need to be captured simultaneously.

#### Add and norm layer:

The add and norm layer performs layer normalization and residual connections, which help stabilize training by ensuring that the output from each layer is added to the input before being normalized. This allows for smoother gradient flow and avoids the vanishing gradient problem.

#### Feed-forward layer:

After the attention mechanism, the feed-forward network is a fully connected neural network (Figure 2e), helping the model learn complex mappings and capture more abstract representations of the input data.

### 2.3 BERT and GPT models

BERT and GPT stand as two exceptional language models. Both BERT and GPT leverage the transformer architecture, employing attention mechanisms to grasp dependencies within input data.

#### BERT (Bidirectional Encoder Representations from Transformers).

BERT is basically an encoder stack of transformer architecture, which was introduced by Google in 2018 [2]. BERT is trained using a bidirectional approach, meaning it considers context from both the left and right of each token during training. This enables BERT to capture richer, more context-aware representations. BERT is typically pre-trained using a masked language model (MLM) task, where random tokens in a sequence are masked, and the model is tasked with predicting them. This bidirectional training allows BERT to better understand the full context of a sequence or biological sentence, such as a cell (Figure 2f). For example, scBERT, a single-cell adaptation of BERT, applies this approach to single-cell RNA-seq data. By masking random gene tokens and predicting them during pretraining, scBERT learns complex dependencies and co-expression patterns between genes. This enables it to capture the full transcriptional context of individual cells, improving downstream tasks like cell type classification.

#### GPT (Generative Pretrained Transformer).

Introduced by OpenAI [1], GPT is based on a decoder stack of transformer architecture. Unlike BERT, GPT uses a unidirectional training approach, processing the input sequence from left to right (Figure 2f). It is trained using autoregressive learning, where each token is predicted based on the previous ones, making it particularly suited for generational tasks. GPT excels in zero-shot learning, where they perform tasks without needing task-specific training data. For example, DNAGPT leverages its pretrained knowledge to perform tasks like predicting DNA motifs or identifying regulatory elements without explicit task-specific training. When prompted with a sequence such as “Find the transcription factor binding motif in the following DNA sequence: AGCTTAGGCC...”, DNAGPT can identify or generate plausible motifs based on its understanding of DNA patterns learned during pretraining.

## 3. Foundation models in bioinformatics

### 3.1 Key components of biological foundation models

**Figure 3.**
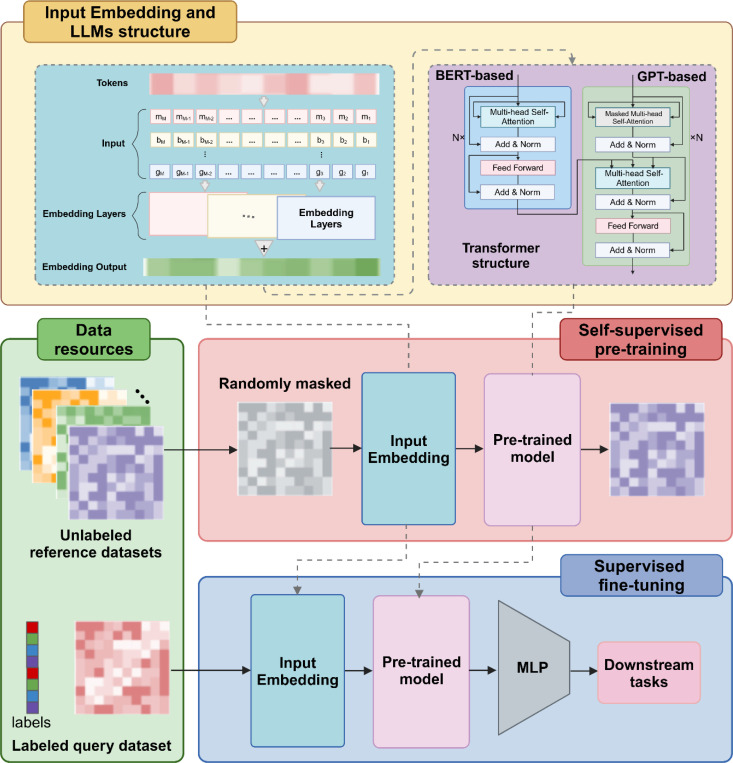
Schematic diagram of the large language model pretraining and fine-tuning process. The workflow begins with tokenizing the input data, which is then fed into the embedding layer and transformer models. The training process comprises two stages: pretraining and fine-tuning. Pretraining employs self-supervised learning on large-scale, unlabeled reference datasets to develop a general-purpose model with robust generalization capabilities. Fine-tuning builds upon the pretrained model, involving task-specific training to optimize performance for designated applications.

Foundation models are a category of large-scale, pre-trained models designed to be versatile and adaptable to various downstream tasks. They are built upon several fundamental components that enable their widespread applicability and effectiveness across domains. First, foundation models are trained on extensive and diverse datasets to capture broad, generalizable patterns. In single-cell biology, for example, datasets with millions of cells spanning multiple tissues and conditions are often used. Second, the architecture of foundation models is typically designed for flexibility and scalability. Their architecture, often transformer-based (e.g., GPT and BERT), are specifically designed for flexibility and scalability. Third, Self-supervised learning is a core training strategy for foundation models. By creating tasks such as masked prediction, contrastive learning, or next-token prediction, models can learn representations without requiring labeled data. Fourth, foundation models exhibit multi-task transferability, leveraging a two-step process of pre-training and fine-tuning (Figure 3). During pre-training, these models are trained on large-scale datasets to develop robust generalization capabilities by capturing broad patterns and knowledge. Fine-tuning involves adapting the pre-trained model to specific tasks by exposing it to unique data and additional training. This approach enables foundation models to adjust effectively to diverse applications while maintaining their versatility across a wide range of domains. Last but not least, training foundation models requires significant computational resources, often involving GPU or TPU clusters. Foundation models typically feature billions or even trillions of parameters.

### 3.2 Foundation models in different biological domains

#### DNA foundation models.

Currently, DNA sequence-based foundation models are powerful tools that leverage advanced deep learning architectures to analyze and interpret genomic data [30]. These models are built on frameworks like BERT and GPT, which have been adapted for the specific challenges of genomic sequences (Table 2). For example, DNABERT [6]is a BERT-based model trained on the human reference genome, enabling it to capture the contextual relationships between nucleotides and perform tasks such as sequence classification and variant prediction. Expanding beyond a single species, Nucleotide Transformer [31] and Genomic Pre-trained Network (GPN) [32] are transformer-based models that incorporate the multiple species reference genomes, providing a broader understanding of genomic diversity. DNABERT-2 [33] takes this a step further by training on multi-species genomic data from 135 species, allowing for cross-species genomic analysis. Similarly, GROVER [34], another BERT-based model, is focused on the human reference genome and is designed for applications such as understanding gene expression and functional genomics. On the other hand, DNAGPT, based on the GPT architecture, is trained not only on the human reference genome but also on reference genomes from nine other species, facilitating tasks such as sequence generation and evolutionary analysis. Together, these DNA sequence-based foundation models represent a leap forward in computational genomics, enabling more accurate predictions, better understanding of genetic variation, and advancements in personalized medicine.

#### RNA foundation models.

RNA sequence-based language models, particularly BERT-based and Transformer-based models, have gained significant traction in the analysis of RNA sequences due to their ability to understand the complex patterns and structures of RNA. These models are trained using a wide variety of RNA types, including non-coding RNAs (ncRNAs), coding RNA, and untranslated regions (UTRs), across diverse organisms (Table 2). For instance, RNABERT [8], RNA-FM [4], RNA-MSM [5], and UNI-RNA [35] focus on all ncRNA types from a broad range of species, enabling insights into RNA function and interactions. Models like SpliceBERT [36] specialize in coding RNA sequences from 72 vertebrates, while 3UTRBERT [37] is specifically designed for human mRNA transcripts, particularly the 3’ untranslated regions. Additionally, UTR-LM [38] focuses on 5’ UTR sequences from five species, and RNAErnie [9], a Transformer-based model, covers a wide range of ncRNAs. These models are part of a rapidly growing field aimed at advancing RNA sequence analysis, facilitating the study of RNA biology and its role in various biological processes and diseases. Through the use of these RNA-based language models, researchers can make significant strides in understanding RNA structure, function, and regulatory mechanisms.

#### Protein foundation models.

Foundation models for proteins can be directly utilized to obtain high-quality protein embeddings and support various downstream applications. The foundational protein models listed in Table 2 not only fulfill these requirements but also exhibit unique characteristics. For example, TAPE [39] made a significant contribution by introducing a comprehensive benchmark for protein bioinformatics tasks. ESM-1b [40] applied the transformer architecture of large language models in a highly standardized manner to protein representation learning. This model has since been widely used to generate protein sequence embeddings, and its variants can also be found via the same link provided in Table 2. ProtTrans [11], compared to ESM-1b, significantly expanded the model architecture, the number of parameters, and the size of the training dataset. It has been widely adopted as a frozen encoder for protein sequences. ProtGPT2 [41], as its name suggests, extends GPT-2 into the protein domain (with links providing details on the GPT-2 training framework). Recent foundation models like ProtBert [42] and KeAP [43] integrate biomedical text information alongside protein sequences. Notably, KeAP incorporates a knowledge graph to enhance this integration. Both models demonstrate that multimodal fusion within proteomics often produces more expressive features. CaLM [14], on the other hand, represents proteins using cDNA, embedding cross-omics biological information. From the perspective of algorithmic advancements, the integration of multimodal information within a single omics domain, as well as cross-omics data fusion, represents key strategies for constructing unified large-scale biological models.

#### Drug discovery foundation models.

It has been postulated that the total number of potential drug like candidates range from 10^23^ to 10^60^ molecules[44]. Foundation models leverage diverse tokenization strategies, embedding techniques, and pre-training mechanisms to enhance molecular representation learning, facilitating the optimization of various downstream tasks (Table 2). For instance, Mol-BERT [45] employs a context-aware tokenizer to encode atoms into chemically meaningful discrete values, although this approach results in an unbalanced atom vocabulary. SMILES-BERT [46], a semi-supervised model incorporating an attention-based Transformer architecture, utilizes datasets such as LogP, PM2, and PCBA-686978 to pre-train the model via a Masked SMILES Recovery (MSR) task. This model demonstrates strong generalization capabilities, enabling its application to diverse molecular property prediction tasks through fine-tuning. Similarly, Mol-GPT [47] facilitates the generation of molecules with specific scaffolds and desired molecular properties by conditioning the generation process on scaffold SMILES strings and property values. Notably, SynerGPT [48] enables a pre-trained GPT model to perform in-context learning of “drug synergy functions”, showcasing potential for future advancements in personalized drug discovery. These foundation models developed based on distinct strategies, effectively learn representations from raw sequence data and molecular descriptors. They provide significant insights into the design of small-molecule drugs, drug-drug interactions, and drug-target interactions.

#### Single-cell foundation models.

Foundation models in single-cell analysis are revolutionizing the field by offering scalable and versatile solutions for a wide range of tasks, leveraging both cell and gene-level representations (Table 2). Models like scBERT [20], tGPT [19], scMulan [26], UCE [49] and CancerFoundation [50] focus on learning robust cell representations, effectively supporting applications such as cell clustering, cell type annotation, batch effect correction, trajectory inference and drug response prediction. These models excel at analyzing heterogeneous cellular populations and uncovering cellular dynamics. In contrast, models like scGPT [21], scFoundation [25], Geneformer [18] GeneCompass [51] and scPRINT [52] combine the ability to learn both cell and gene-level representations. They capture inter-gene relationships and regulatory networks, making them highly effective for tasks such as gene expression profiling, gene regulatory network (GRN) inference, gene perturbation prediction, and drug dose-response prediction. Notably, scGPT can also handle single-cell multi-omics data, facilitating tasks like scRNA-seq and scATAC-seq integration. Another notable model is Nicheformer [53], a foundation model specifically designed for spatial transcriptomics. It focuses on learning cell representations while being highly adaptable to various downstream tasks in spatial transcriptomics, such as spatial label prediction (e.g., cell type, niche, and region labels), niche composition analysis, and neighborhood density prediction. Additionally, Nicheformer can generate joint embeddings of scRNA-seq and spatial transcriptomics data, facilitating the integration of these modalities for a more comprehensive understanding of cellular and spatial interactions.

## 4. Applications of large language models in bioinformatics

**Figure 4.**
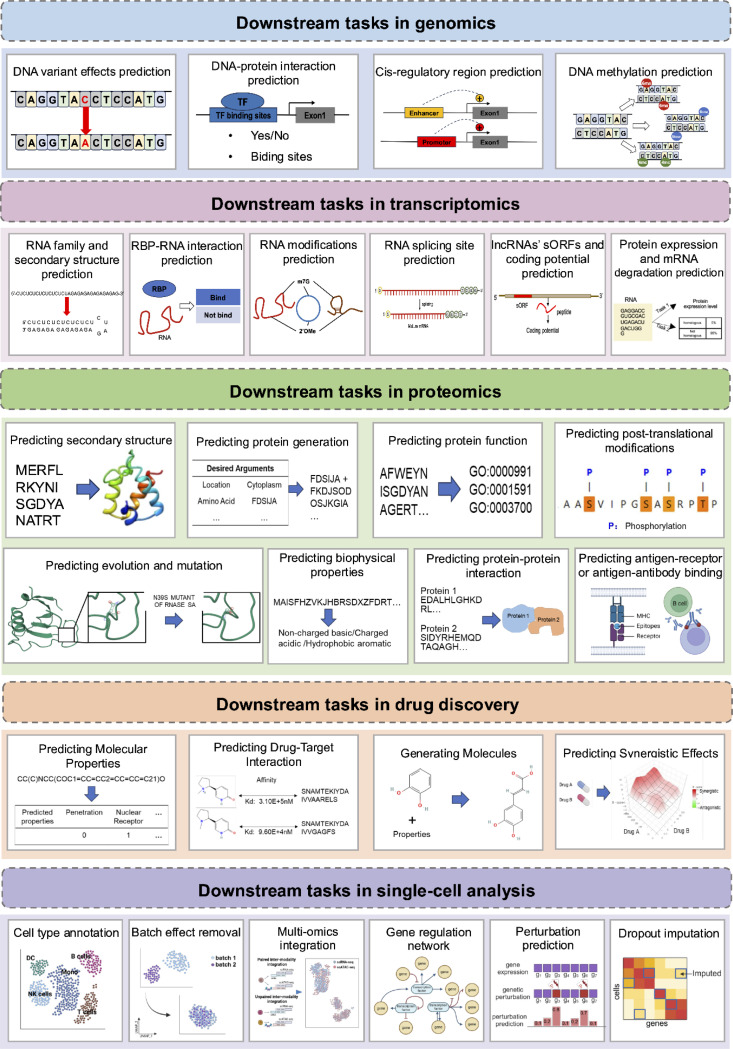
Downstream tasks of large language models in bioinformatics. Large language models (LLMs) have seen numerous successful applications in bioinformatics, addressing a wide array of tasks across DNA, RNA, protein, drug discovery, and single-cell analysis.

Large language models (LLMs) have seen numerous successful applications in bioinformatics, addressing a wide array of tasks across DNA, RNA, protein, drug discovery, and single-cell analysis (Figure 4). These applications highlight the adaptability and potential of LLMs in overcoming bioinformatic challenges, enabling deeper insights into complex biological systems and fostering advancements across multiple domains.

### 4.1 Applications of large language models in genomics

The DNA language models take DNA sequence as input, use transformer, BERT, GPT models to solve multiple biological tasks, including genome-wide variant effects prediction, DNA cis-regulatory regions prediction, DNA-protein interaction prediction, DNA methylation (6mA,4mC 5hmC) prediction, splice sites prediction from DNA sequence (Table 3, Supplementary Figure 1). A detailed list of DNA language models, their downstream tasks, and the datasets used can be found in Supplementary Table 1.

#### Genome-wide variant effects prediction.

Genome-wide variant effects prediction is crucial for understanding the role of DNA mutations in species diversity. Genome-wide association studies (GWAS) provide valuable insights but often struggle to identify specific causal variants [30, 54]. The Genome Prediction Network (GPN) [32] addresses this by using unsupervised pre-training on genomic DNA sequences. During this process, GPN predicts nucleotides at masked positions within a 512-bp DNA sequence. This model is particularly effective at predicting rare variant effects, often missed by traditional GWAS methods. Additionally, models like DNABERT, DNABERT-2, and the Nucleotide Transformer also predict variant effects from DNA sequences. These advancements highlight ongoing efforts to better understand how DNA mutations contribute to biological diversity.

#### Cis-regulatory regions prediction.

Cis-regulatory sequences, such as enhancers and promoters, play crucial roles in gene expression regulation, influencing development and physiology [55]. However, identifying these sequences remains a major challenge [56]. Pre-trained models like DNABERT, DNABERT-2, GROVER, and DNAGPT have been developed to predict promoter regions and their activities with high accuracy. BERT-Promoter [57] utilizes a pre-trained BERT model for feature representation and SHAP analysis to filter data, improving prediction performance and generalization over traditional methods. Enhancers, which bind transcription factors to regulate gene expression [58, 59], are predicted by iEnhancer-BERT [60], which leverages DNABERT and uses a novel transfer learning approach. This model employs output from all transformer encoder layers and classifies features with a Convolutional Neural Network (CNN). These advancements highlight the growing trend of treating biological sequences as a natural language for computational modeling, offering new tools for identifying cis-regulatory regions and understanding their roles in diseases.

#### DNA-protein interaction prediction.

Accurate identification of DNA-protein interactions is crucial for gene expression regulation and understanding evolutionary processes [61]. Several DNA language models, including DNABERT, DNABERT-2, and GROVER, have been developed to predict protein-DNA binding from ChIP-seq data. TFBert [62] is a pre-trained model specifically designed for DNA-protein binding prediction, which treats DNA sequences as natural sentences and k-mer nucleotides as words, allowing effective context extraction. Pre-trained on 690 ChIP-seq datasets, TFBert delivers strong performance with minimal fine-tuning. The MoDNA [63] framework introduces domain knowledge by incorporating common DNA functional motifs. During self-supervised pre-training, MoDNA performs tasks such as k-mer and motif prediction. Pre-training on extensive unlabeled genome data, MoDNA acquires semantic-level genome representations, enhancing predictions for promoter regions and transcription factor binding sites. Essentially, MoDNA functions as a biological language model for DNA-protein binding prediction.

#### DNA methylation prediction.

DNA methylation is a key biological process in epigenetic regulation and is linked to various medical conditions and applications, such as metagenomic binning [64]. DNA methylation types depend on the nucleotide where the methyl group attaches [65]. Several models predict DNA methylation with varying accuracy. BERT6mA [66] is designed for predicting 6-methyadenine (6mA) sites, while iDNA-ABT [67], iDNA-ABF [68], and MuLan-Methyl [69] are versatile models predicting various methylation types (6mA, 5hmC, 4mC). iDNA-ABT, a deep learning model, integrates BERT with transductive information maximization (TIM), though it has yet to fully explore feature representation. iDNA-ABF uses a multi-scale architecture, applying multiple tokenizers for diverse embeddings, and MuLan-Methyl employs four transformer-based models (DistilBERT [70], ALBERT[71], XLNet [72], and ELECTRA [73]) to predict methylation sites, enhancing performance through joint model utilization.

#### DNA level splice site identification.

Accurate pre-mRNA splicing is essential for proper protein translation, driven by splice site selection. Identifying splice sites is challenging, particularly with prevalent GT-AG sequences [74]. To address this, DNABERT and DNABERT-2 were developed, trained on 10,000 donors, acceptor, and non-splice site sequences from the human reference genome to predict splice sites. DNABERT showed high attention to intronic regions, suggesting the functional role of intronic splicing enhancers and silencers as cis-regulatory elements in splicing regulation. This highlights DNABERT’s potential in understanding splicing mechanisms.

### 4.2 Applications of large language models in transcriptomics

The RNA language models take RNA sequences as input, use transformer, BERT, GPT models to solve multiple biological tasks, including RNA 2D/3D structure prediction, RNA structural alignment,, RNA family clustering, RNA splice sites prediction from RNA sequence, RNA N7-methylguanosine modification prediction, RNA 2’-O-methylation modifications prediction, multiple types of RNA modifications prediction, predicting the association between miRNA, lncRNA and disease, identifying lncRNAs, lncRNAs’ coding potential prediction, protein expression and mRNA degradation prediction (Table 3, Supplementary Figure 1). A detailed list of RNA language models, their downstream tasks, and the datasets used can be found in Supplementary Table 1.

#### Secondary structure prediction.

RNA secondary structure prediction is a major challenge for RNA structural biologists, with models holding potential for RNA-targeting drug development [75]. Several RNA language models, such as RNABERT [8], RNA-MSM [5], RNA-FM [4], and UNI-RNA [35], have been developed to predict RNA structures with varying sophistication. RNABERT uses BERT architecture to predict structural features like base-pairing and stem loops. RNA-MSM integrates sequence and structural information to predict local and long-range folding patterns. RNA-FM focuses on RNA folding, stability, and energetics, including pseudoknots. UNI-RNA combines sequence and structure predictions across various RNA types. These models advance RNA structure prediction by applying deep learning and advanced techniques to improve understanding of RNA folding and function.

#### RNA splicing prediction.

RNA splicing is crucial for gene expression in eukaryotes, and advancements have been made in sequence-based splicing modeling through models like SpliceBERT [36] and UNI-RNA [35]. SpliceBERT, based on BERT, is trained to predict RNA splicing events by capturing long-range dependencies, identifying splice sites, and predicting alternative splicing events. UNI-RNA, a more generalized model, integrates multiple RNA tasks, including splicing, and combines sequence and structural data to predict splicing regulatory elements and interactions with splicing factors. These models enhance the understanding of RNA splicing, gene regulation, and its role in diseases, providing powerful tools for studying splicing defects and mutations.

#### lncRNAs identification and lncRNAs’ coding potential prediction.

Long non-coding RNAs (lncRNAs) play significant regulatory roles in cancer and diseases, and their small Open Reading Frames (sORFs), once thought weak in protein translation, are now known to encode peptides [76]. Identifying lncRNAs with sORFs is crucial for discovering new regulatory factors. LncCat [77] addresses this challenge by using category boosting and ORF-attention features, including BERT for peptide sequence representation, to improve prediction accuracy for both long ORF and sORF datasets. It demonstrates effectiveness across multiple species and Ribo-seq datasets in identifying lncRNAs with sORFs. In predicting translatable sORFs in lncRNAs (lncRNA-sORFs), LSCPP-BERT [78] is a novel method designed for plants, leveraging pre-trained transformer models for reliable coding potential prediction. LSCPP-BERT is poised to impact drug development and agriculture by enhancing understanding of lncRNA coding potential.

#### RNA–RBP interactions prediction.

RNA sequences differ from DNA sequences by a single base (thymine to uracil), maintaining largely congruent syntax and semantics. BERT’s versatility extends to Cross-linking and Immunoprecipitation data, particularly in predicting RNA-binding protein (RBP) binding preferences. BERT-RBP [79] is a model pre-trained on a human reference genome, designed to forecast RNA-RBP interactions. It outperforms existing models when tested on eCLIP-seq data from 154 RBPs and can identify transcript regions and RNA secondary structures based on sequence alone. BERT-RBP demonstrates BERT’s adaptability in biological contexts and its potential to advance RNA-protein interaction understanding.

#### RNA-RNA interaction prediction.

RNA–RNA interactions occur between various RNA species, including long non-coding RNAs, mRNAs, and small RNAs (e.g., miRNAs and lncRNAs), driven by complementary sequences, secondary structures, and other motifs [80]. Accurate prediction of these interactions provides insights into RNA-mediated regulation, enhancing understanding of biological processes like gene expression, splicing, and translation. RNAErnie, used for this purpose, employs a TBTH architecture combining RNAErnie with a hybrid network (CNN, Bi-LSTM, and MLP) to predict RNA–RNA interactions. This approach demonstrates RNAErnie’s potential in advancing RNA-based regulatory network studies.

#### RNA modification prediction.

Post-transcriptional RNA modifications, such as N7-methylguanosine (m7G) and 2’-O-methylation (Nm), regulate gene expression and are linked to diseases [76, 81]. Identifying modification sites is essential but challenging due to the high cost and time required by experimental methods. Computational tools like BERT-m7G [82] and Bert2Ome [83] address this issue. BERT-m7G uses a stacking ensemble approach to identify m7G sites directly from RNA sequences, offering an efficient, cost-effective alternative. Bert2Om combines BERT and CNN to predict 2’-O-methylation sites, outperforming existing methods across datasets and species. These tools enhance the accuracy, scalability, and efficiency of RNA modification site identification, advancing research into RNA modifications and their roles in gene regulation and disease.

#### Protein expression and mRNA degradation prediction.

mRNA vaccines are a cost-effective, rapid, and safe alternative to traditional vaccines, showing high potency [84]. These vaccines work by introducing mRNA that encodes a viral protein. CodonBERT [85] is a model specifically designed for mRNA sequences to predict protein expression. It uses a multi-head attention transformer architecture and was pre-trained on 10 million mRNA sequences from various organisms. This pre-training enables CodonBERT to excel in tasks like protein expression and mRNA degradation prediction. Its ability to integrate new biological information makes it a valuable tool for mRNA vaccine development. CodonBERT surpasses existing methods, optimizing mRNA vaccine design and improving efficacy and applicability in immunization. Its strength in predicting protein expression enhances mRNA vaccine development efficiency and effectiveness.

#### 5’ UTR-based mean ribosome loading prediction and mRNA subcellular localization prediction.

The 5’ UTR sequence plays a critical role in regulating translation efficiency. RNA sequence models like 3UTRBERT, UNI-RNA, UTR-LM, RNA-FM, and Nucleotide Transformer have been developed to predict key features of the 5’ UTR, focusing on ribosome loading efficiency and mRNA localization. These models use Transformer-based architecture to analyze sequence patterns, motifs, and structural elements. For example, 3UTRBERT [37] and RNA-FM [4] predict ribosome loading efficiency, identifying regions likely to recruit ribosomes for translation initiation. UTR-LM [38], UNI-RNA [35], and Nucleotide Transformer [31] predict mRNA subcellular localization, determining where mRNA will localize in the cell (cytoplasm, ribosomes, or nucleus), which is crucial for regulating mRNA stability and translation. Together, these models provide valuable insights into gene expression, translation control, and RNA localization, advancing molecular biology research.

### 4.3 Applications of large language models in proteomics

Protein is an indispensable molecule in life, assuming a pivotal role in the construction and sustenance of vital processes. As the field of protein research advances, there has been a substantial surge in the accumulation of protein data [86]. In this context, the utilization of large language models emerges as a viable approach to extract pertinent and valuable information from these vast reservoirs of data. Several pre-trained protein language models (PPLMs) have been proposed to learn the characteristic representations of proteins data (e.g., protein sequences, gene ontology annotations, property descriptions), then applied to different tasks by fine-tuning, adding or altering downstream networks, such as protein structure, post-translational modifications (PTMs), and biophysical properties, which align with corresponding downstream tasks like secondary structure prediction, major PTMs prediction, and stability prediction [87, 88].

Even though antibodies are classified as proteins, the datasets of antibodies and subsequent tasks differ significantly from those of proteins. Through the establishment and continuous updates of the Observed Antibody Space (OAS) database [89], a substantial amount of antibody sequence data has become available, which can be utilized to facilitate the development of pre-trained antibody large language models (PALMs). PALMs primarily delve into downstream topics encompassing therapeutic antibody binding mechanisms, immune evolution, and antibody discovery, which correspond to tasks like paratope prediction, B cell maturation analysis, and antibody sequence classification (Table 3, Supplementary Figure 2).

In this section, some of the popular protein-related large language models of recent years are introduced, as well as corresponding important downstream tasks. It is important to emphasize that both PPLM and PALM are capable of handling not only the downstream tasks introduced in this section. For further details, additional information can be referenced within Supplementary Table 2.

#### Secondary structure and contact prediction.

Protein structure is critical to its function and interactions [90]. However, traditional experimental techniques for protein structure analysis are time-consuming and labor-intensive. With the rise of deep learning, large language models have demonstrated significant advantages in computational efficiency and prediction accuracy for protein structure prediction [91]. MSA Transformer [92] introduces a protein language model that processes MSAs using a unique mechanism of interleaved row and column attention. Trained with a MLM objective across diverse protein families, it outperformed earlier unsupervised approaches and showed greater parameter efficiency than previous models. Drawing on insights from BERT, large parameter models tend to achieve better performance for predicting secondary structures and contacts. Few models have more parameters than the largest models in ProtTrans [11], which includes a series of autoregressive models (Transformer-XL [93], XLNet [72]) and four encoder (BERT [2], Albert [71], Electra [73], T5 [94]) trained on datasets like UniRef [95] and BFD [96], comprising up to 393 billion amino acids. Model sizes vary from millions to billions of parameters. Notably, ProtTrans made a significant breakthrough in per-residue predictions.

#### Protein sequence generation.

Protein sequence generation holds significant potential in drug design and protein engineering [97]. Using machine learning or deep learning, generated sequences aim for good foldability, stable 3D structures, and specific functional properties, such as enzyme activity and antibody binding. The development of large language models, combined with conditional models, has greatly advanced protein generation [98]. ProGen [12] incorporates UniprotKB keywords as conditional tags, covering over 1,100 categories like ‘biological process’ and ‘molecular function’. Proteins generated by ProGen, assessed for sequence similarity, secondary structure, and conformational energy, exhibit desirable structural properties. In 2022, ProtGPT2 [41] inspired by GPT-x was developed. ProtGPT2-generated proteins show amino acid propensities like natural proteins. Prediction of disorder and secondary structure reveals that 88% of these proteins are globular, resembling natural sequences. Employing AlphaFold [99, 100] on ProtGPT2 sequences produces well-folded, non-idealized structures with unique topologies not seen in current databases, suggesting ProtGPT2 has effectively learned “protein language”.

#### Protein function prediction.

Proteins are essential in cellular metabolism, signal transduction, and structural support, making their function critical for drug development and disease analysis. However, predicting and annotating protein functions is challenging due to their complexity. PPLMs offer effective solutions to these challenges [101, 102]. ProtST [103] introduced a multimodal framework combining a PPLM for sequences and a biomedical language model (BLM) for protein property descriptions. Through three pre-training tasks, unimodal mask prediction, multimodal representation alignment, and multimodal mask prediction, the model excels in tasks like protein function annotation, zero-shot classification, and functional protein retrieval from large databases. While most methods focus on increasing model parameters to improve performance, CaLM [14] introduces an alternative representation, the cDNA sequence, akin to an amino acid sequence, as input. The core idea lies in the relationship between synonymous codon usage and protein structure [104], and the information encoded in codons is no less than that of amino acids. Experimental results demonstrate that even with a small parameter language model, using cDNA sequences as input enhances performance in tasks such as protein function prediction, species recognition, prediction of protein and transcript abundance, and melting point estimation.

#### Major post-translational modification prediction.

Post-translational modifications (PTMs) are chemical changes, such as phosphorylation, methylation, and acetylation, that alter protein structure and function after translation. PTMs influence protein stability, localization, interactions, and function, making their study crucial for disease diagnosis and therapeutic strategies [105, 106]. Language models can effectively predict PTMs and related tasks like signal peptide prediction. ProteinBERT [42] , with only ~16M parameters, is not large enough but performs well due to its inclusion of Gene Ontology (GO) annotation tasks. By incorporating GO interactions with protein sequences, ProteinBERT achieves strong performance on PTM prediction and other protein property benchmarks, outperforming models with larger parameter sizes.

#### Evolution and mutation prediction.

Protein evolution and mutation drive functional diversity, aiding adaptation to environmental changes and offering insights into protein function origin, which can inform drug development and disease treatment [107, 108]. UniRep [109], built on the LSTM architecture, was trained on the UniRef50 [95] and excelled in tasks like remote homology detection and mutation effect prediction. ESM-1b [40] , a deep transformer model trained on 250 million sequences, with 33 layers and 650 million parameters, captures essential protein sequence patterns through self-supervised learning. ESM-1b is also integral to frameworks like PLMSearch [110] and DHR [111] , which enable fast, sensitive homology searches. PLMSearch uses supervised training, while DHR relies on unsupervised contrastive learning and enhances structure prediction models like AlphaFold2 [100].

#### Biophysical properties prediction.

Biophysical properties of proteins, such as fluorescence and stability landscapes [112], are crucial for understanding protein folding, stability, and conformational changes, with significant implications for drug design, protein engineering, and enzyme engineering. Deep learning advancements have enabled more accurate prediction of these properties using PPLMs. TAPE benchmark [39] established standardized tasks for evaluating protein, including fluorescence and stability landscape prediction. In 2022, PromptProtein [113], a prompt-based pre-trained model, incorporated multi-task pre-training and a fine-tuning module to improve task-specific performance. It outperformed existing methods in function and biophysical properties prediction, demonstrating substantial gains in predictive accuracy.

#### Protein-protein interaction and binding affinity prediction.

Protein-protein interactions (PPIs) are crucial for biological functions, and their prediction is also vital for drug discovery and design. PPLMs provide efficient, accurate predictions of PPI types and binding affinities [114, 115]. KeAP model [43], like ProtST, aims to integrate fine-grained information beyond OntoProtein [116]. KeAP uses a triplet format (Protein, Relation, Attribute) as input, processed by encoders and a cascaded decoder based on the Transformer architecture. Using MLM for pre-training, KeAP employs a cross-attention fusion mechanism to capture detailed protein information, achieving superior performance on tasks such as PPI identification and binding affinity estimation.

#### Antigen-receptor binding and antigen-antibody binding prediction.

Antigen proteins are processed into neoantigen peptides that bind to the Major Histocompatibility Complex (MHC), forming pMHC complexes. These complexes are presented to T-cells, stimulating antibody production by B-cells, which triggers an immune response [117] . Predicting peptide binding to MHC molecules is a key focus of language models in this process [118, 119]. MHCRoBERTa [120] uses a pretrained BERT model to predict pMHC-I binding by learning the biological meaning of amino acid sequences. BERTMHC [121], trained on 2,413 MHC–peptide pairs, focuses on pMHC-II binding prediction, filling a gap in this area.

Another goal is predicting the binding specificity of adaptive immune receptors (AIRs), particularly TCRs. TCR-BERT [122] learns TCR CDR3 sequences to predict antigen specificity but lacks the ability to model the interaction between TCR chains. SC-AIR-BERT [123] addresses this by pre-training a model that outperforms others in TCR and BCR binding specificity. Additionally, the Antiformer [124] integrates RNA-seq and BCT-seq data in a graph-based framework to improve antibody development. In antibody modeling, three recent models focus on unique tasks. AbLang [125], built on RoBERTa [126], excels at restoring lost residues during sequencing and outperforms other models in accuracy and efficiency. AntiBERTa [127] understands antibody “language” through tasks like predicting immunogenicity and binding sites. EATLM [128] , with its unique pre-training tasks (Ancestor Germline Prediction and Mutation Position Prediction), contributes a reliable benchmark for antibody language models.

### 4.4 Applications of large language models in drug discovery

Drug discovery is an expensive and long-term process that exhibits a low success rate. During the early stages of drug discovery, computer-aided drug discovery, employing empirical or expert knowledge algorithms, machine learning algorithms, and deep learning algorithms, serve to accelerate the generation and screening of drug molecules and their lead compounds [129–131]. It speeds up the entire drug discovery process, especially the development of small molecule drugs. Among commonly used medications, small molecule drugs can account for up to 98% of the total [132]. The structure of small molecule drugs exhibits excellent spatial dispersibility, and their chemical properties determine their good drug-like properties and pharmacokinetic properties [133]. With the development of deep learning and the proposal of large language models, it has become easy to apply these methods to discover hidden patterns of molecules and interactions between molecules for drugs (such as small molecules) and targets (such as proteins and RNA) that can be easily represented as sequence data. The Simplified Molecular-Input Line-Entry System (SMILES) string and chemical fingerprint are commonly used to represent molecules. Additionally, through the pooling process of graph neural networks(GNN), small molecules can be transformed into sequential representations [134]. With the protein sequence, large language models can engage in drug discovery through various inputs. Within this section, key tasks within the early drug discovery process that have effectively leveraged large language models will be introduced (Table 3, Supplementary Figure 3). A detailed list of drug discovery language models, their downstream tasks, and the datasets used can be found in Supplementary Table 3.

#### Drug-like molecular properties prediction.

In drug discovery, significant focus is placed on properties like ADMET and PK to develop more effective, accessible, and safe drugs[135, 136]. Large language models (LLMs) are used for molecular property prediction, including these properties. Since molecular SMILES representations are consistent, models can be easily improved and fine-tuned for specific tasks based on researchers’ requirements. SMILES-BERT [17] departed from the usage of knowledge-based molecular fingerprints as input. Instead, it adopted a representation method where molecules were encoded as SMILES sequences and employed as input for both pre-training and fine-tuning within a BERT-based model. This novel approach yielded superior outcomes across various downstream molecular property prediction tasks, surpassing the performance of previous models reliant on molecular fingerprints. ChemBERTa [137] is a BERT-based model that focuses on the scalability of large language models, exploring the impact of pre-training dataset size, tokenizer, and string representation. Subsequently, ChemBERTa-2[138] improved upon ChemBERTa by using a larger dataset of 77 million compounds from PubChem, enhancing its ability to learn from diverse chemical structures. It also integrates advanced self-supervised learning techniques and fine-tuning strategies, resulting in better generalization performance across various downstream tasks. K-BERT [15] stands out by using three pre-training tasks: atom feature prediction, molecular feature prediction, and contrastive learning. This approach enables the model to understand the essence of SMILES representations, resulting in exceptional performance across 15 drug datasets, highlighting its effectiveness in drug discovery. Given the importance of graph neural networks in the development of molecular pre-training models, Mole-BERT [139] introduces atom-level Masked Atoms Modeling (MAM) task and graph-level Triplet Masked Contrastive Learning (TMCL) task. These tasks enable the network to acquire a comprehensive understanding of the “language” embedded within molecular graphs. By adopting this approach, the network demonstrates exceptional performance across eight downstream tasks, showcasing its adaptability and effectiveness in diverse applications.

#### Drug-like molecules generation.

It is very difficult to chase the full coverage of the enormous drug-like chemical space (estimated at more than 10^63^ compounds), and traditional virtual screening libraries usually contain less than 10^7^ compounds and are sometimes not available. In such circumstances, the utilization of deep learning methods to generate molecules exhibiting drug-like properties emerges as a viable approach [140, 141]. Inspired by the generative pre-training model GPT, MolGPT [16] model was introduced. In addition to performing the next token prediction task, MolGPT incorporates an extra training task for conditional prediction, facilitating the capability of conditional generation. Beyond its capacity to generate innovative and efficacious molecules, the model has demonstrated an enhanced ability to capture the statistical characteristics within the dataset.

#### Drug-target interaction predictions.

The investigation of Drug-Target Interaction (DTI) holds paramount significance in the realm of drug development and the optimization of drug therapy. Understanding drug-target interactions aids in pharmaceutical design, accelerates drug development, and reduces time and resource costs in lab experimentation and trial-and-error methods [142, 143]. During the exploration of DTI, diligent focus is placed on the prediction of drug-target binding affinity. DTI-BERT employs a fine-tuned ProtBERT [144] model to process protein sequences and applies discrete wavelet transform to drug molecular fingerprints.. TransDTI [145] is a multi-class classification and regression workflow. This model not only uses fine-tuned SMILES-BERT to extract drug features, but also expands the selection of fine-tuned large protein models. After acquiring potential representations of drug-target pairs, the authors subject the representations to downstream neural networks for the completion of a multi-classification task. Additionally, The Chemical-Chemical Protein-Protein Transferred DTA (C2P2) [146] method uses pre-trained protein and molecular large language models to capture the interaction information within molecules. Given the relatively limited scale of the DTI dataset, C2P2 leverages protein-protein interaction (PPI) and chemical-chemical interaction (CCI) tasks to acquire knowledge of intermolecular interactions and subsequently transfer this knowledge to affinity prediction tasks [147]. It is worth highlighting that in scenarios involving the docking or when emphasizing the spatial structure of a complex, methodologies incorporating 3D convolution networks, point clouds-based networks, and graph networks are often employed [148–151]. In situations where the molecular structure is unknown, but the sequence is available, the prediction of DTI using large-scale models still holds significant promise.

#### Drug synergistic effects predictions.

Combination therapy is common for complex diseases like cancer, infections, and neurological disorders, often surpassing single-drug treatments. Predicting drug pair synergy, where combining drugs boosts therapeutic effects, is vital in drug development. However, it’s challenging due to many drug combinations and complex biology [152, 153]. Various computational methods, including machine learning, help predict drug pair synergy. Carl Edwards et al. introduced SynerGPT [48], which is based on GPT trained to in-context learn drug synergy functions without relying on domain-specific knowledge. Wei Zhang et al. [154] introduced DCE-DForest [154], a model for predicting drug combination synergies. It uses a pretrained drug BERT model to encode the drug SMILES and then predicts synergistic effects based on the embedding vectors of drugs and cell lines using the deep forest method. Mengdie Xua et al. [155] utilized a fine-tuned pre-trained large language model and a dual feature fusion mechanism to predict synergistic drug combinations. Its input includes hashed atom pair molecular fingerprints of drugs, SMILES string encodings, and cell line gene expressions. They conducted ablation analyses on the dual feature fusion network for drug-drug synergy prediction, highlighting the significant role of fingerprint inputs in ensuring high-quality drug synergy predictions.

### 4.5 Applications of large language models in single-cell analysis

Large language models have demonstrated significant applications in single-cell analysis, including cell-level tasks such as identifying cell types, determining cell states, and discovering novel cell populations; gene-level tasks like inferring gene regulatory networks; and multi-omics tasks, such as integrating single-cell multi-omics (scMulti-omics) data (Supplementary Figure 4). Additionally, this section will explore emerging language models based on spatial transcriptomics (Table 3). A detailed list of single-cell large language models, their downstream tasks, and the datasets used can be found in Supplementary Table 4.

#### Cell-level tasks.

Cell-level tasks, such as cell clustering, cell type annotation, novel cell type discovery, batch effect removal and trajectory inference, are critical in single-cell analysis. These tasks often rely on cell representations learned during pretraining, which are subsequently fine-tuned for different tasks. Single-cell language models derive cell representations in two primary ways. The first method utilizes a special class token (<cls>) appended to the input sequence; its embedding is updated through the transformer layers, and the final embedding at the <cls> position serves as the cell representation. The second method generates a cell embedding matrix from the model output, where each row represents a specific cell. Both approaches facilitate downstream tasks, as demonstrated by TOSICA [22], which uses the <cls> token to predict cell type probabilities using the whole conjunction neural network cell type classifier to annotate single cells, and iSEEK [156], which generates cell embedding for cell clustering, cell type annotation, and developmental trajectory exploration. Models like scBERT [20] and UCE [49] leverage multi-head attention mechanisms to extract information from diverse representation subspaces, discerning subtle differences between novel and known cell types. Their large receptive fields capture long-range gene-gene interactions, enabling comprehensive characterization of novel cellular states. Addressing batch effects, which arise from variations in species, tissues, operators, and experimental protocols, remains a significant challenge in single-cell analysis. Large language models, pretrained on extensive datasets, utilize attention mechanisms to incorporate prior biological knowledge, enabling batch-insensitive data annotation. Without relying on explicit batch information, models such as CIForm [152] have demonstrated effectiveness in both intra-dataset and inter-dataset scenarios. They handle annotations across diverse species, organs, tissues, and technologies while also supporting the integration of reference and query data from various sequencing platforms or studies. This capability allows them to address batch effects in single-cell analysis. Drug response or sensitivity prediction is a classification task akin to cell type annotation, where a classifier is appended to the learned cell embeddings to predict whether a cell will respond to or exhibit sensitivity to a specific drug. Models like scFoundation [25] and CellLM [157] effectively utilize this approach, leveraging the robust cell representations learned during pretraining to enhance prediction accuracy.

#### Gene-level tasks.

Gene-level tasks, such as gene expression prediction, gene regulatory network (GRN) inference, gene perturbation prediction, and drug dose-response prediction, are integral to understanding single-cell transcriptomics. Self-attention mechanisms have transformed deep learning by enabling context-aware models that prioritize relevant elements in large input spaces. These models, particularly transformers, are well-suited for modeling the context-dependent dynamics of gene regulatory networks. By focusing on key interactions, transformers can effectively capture the complexities of regulatory relationships, such as the attention matrix in Geneformer [18] and scGPT [21] reflect which genes that gene pays attention to and which genes pay attention to that gene, aiding to infer gene regulation network. Geneformer is pretrained on a vast repository of single-cell transcriptomes to learn gene relationships for diverse downstream applications, including predicting dosage-sensitive disease genes, identifying downstream targets, forecasting chromatin dynamics, and modeling network dynamics. In addition, after pretraining and fine-tuning, single-cell language models output gene embeddings that can be utilized for functional analysis of scRNA-seq data. For instance, scGPT [21] serves as a generalizable feature extractor leveraging zero-shot learning, enabling applications in gene expression prediction and genetic perturbation prediction. Similarly, in scFoundation [25], zero-expressed genes and masked genes are combined with the output from the transformer-based encoder. This combined information is then input into the decoder and projected to gene expression values through a multilayer perceptron (MLP). The gene context expression is employed to formulate a cell-specific gene graph, facilitating the prediction of perturbations using the GEARS [158] model. It is worth noting that genes have a lot of prior knowledge that can be used to enhance many gene-level tasks. For example, GeneCompass [51] incorporates four types of biological prior knowledge, including GRN, promoter information, gene family annotation and gene-co-expressed relationship, making it capable for various gene tasks.

#### scMulti-omics tasks.

Studying single-cell multi-omics data requires integrating diverse information from genomics, transcriptomics, epigenomics, and proteomics at the single-cell level. The adaptability, generalization capabilities, and feature extraction strengths of large language models make them effective in addressing challenges such as feature variance, data sparsity, and cell heterogeneity inherent in single-cell multi-omics datasets. scMulti-omics integration can be viewed as a specialized form of batch effect removal. For example, scGPT [21] treats each modality as a distinct batch and incorporates a special modality token to represent input features (such as genes, regions, or proteins) associated with each modality. This approach helps the transformer model balance attention across modalities, preventing overemphasis on intra-modality features while integrating inter-modality relationships effectively. Another approach involves processing different modalities through separate transformers before projecting their embeddings into a common latent space. Models like scMVP [159] use mask attention-based encoders for scRNA-seq data and transformer-based multi-head self-attention encoders for scATAC-seq. By aligning variations between different omics in this latent space, scMVP captures joint profiling of scRNA-seq and scATAC-seq, achieving paired integration where gene expression and chromatin accessibility are studied within the same cells. Graphs are increasingly recognized as powerful tools for characterizing feature heterogeneity in scMulti-omics integration. For example, DeepMAPS [160] leverages graph transformers to construct cell and gene graphs, learning both local and global features that build cell-cell and gene-gene relationships for data integration, inference of biological networks from scMulti-omics data and cell-cell communication.

Recent advances in sequencing technologies that capture multiple modalities within the same cell have enabled the development of computational tools for cross-modality prediction. One approach involves training large language models on paired datasets to predict one modality from another. For instance, scTranslator [161], pre-trained on paired bulk and single-cell data, fine-tunes to infer protein abundance from scRNA-seq data by minimizing the mean squared error (MSE) between predicted and actual protein levels. Another strategy leverages graph learning with prior knowledge to model feature relationships. For example, scMoFormer [162] can not only translate gene expression to protein abundance, but is also applicable to multi-omics predictions, including protein abundance to gene expression, chromatin accessibility to gene expression, gene expression to chromatin accessibility using graph transformers. Taking protein prediction task as an example, scMoFormer constructs cell-gene graph, gene-gene graph, protein-protein graph, and gene-protein graph based on gene expression profiles and prior knowledge from STRING database [163]. Each modality has a separate transformer to learn the global information that may not be included in prior knowledge. Message-passing graph neural networks (GNNs) link nodes across various graphs, while transformers are employed to precisely map gene expression to protein abundance.

#### Spatial transcriptomics tasks.

The rapid development of single-cell and spatial transcriptomics has advanced our understanding of cellular heterogeneity and tissue architecture. Spatial transcriptomics retains cells’ native spatial context, enabling insights into cellular interactions. Large language models address the challenge of high-dimensional spatial data analysis by integrating spatial and molecular information, enhancing tissue-specific pattern interpretation. For example, Nicheformer [53] is the latest large language model in spatial transcriptomics. It integrates extensive spatial transcriptomics and single-cell transcriptomics data, leveraging metadata across multiple modalities, species, and sequencing technologies. By doing so, Nicheformer is capable of learning joint information from single-cell and spatial transcriptomics, enabling the resolution of various spatial prediction tasks even with limited data. Spaformer [164] is another transformer-based model designed for spatial transcriptomics data. Spaformer is designed to address two key challenges: how to encode spatial information of cells into a transformer model and how to train a transformer to overcome the sparsity of spatial transcriptomics data, enabling data imputation. Spatial transcriptomics, as one of the most popular technologies in recent years, focuses on integrating single-cell resolution gene expression data with tissue spatial information to reveal spatial relationships and functional characteristics among cells. However, large language models (LLMs) specifically designed for spatial transcriptomics are still in their early stages of development. The creation of these models faces unique challenges, such as effectively integrating high-dimensional gene expression data with complex spatial information and addressing the sparsity and irregularity of the data.

In addition to the single-cell large language models discussed above, another category of single-cell prediction models leverages natural language, utilizing textual data such as human-readable descriptions of gene functions and biological features to support various single-cell analyses. For example, GPT-4 [165] leverages its strong contextual understanding for interpreting high-dimensional single-cell analysis for accurate cell type annotation. GenePT [166] utilizes OpenAI’s ChatGPT text embedding to classify gene properties and cell types effectively. More and more models demonstrate that natural language pretraining can significantly boost performance on single-cell downstream tasks, including cell generation [167], cell identity (e.g., cell type, pathway, and disease information) [167–171], and gene enrichment analysis [169]. These models demonstrate significant potential in advancing single-cell analysis by integrating natural language processing techniques. However, the reliance on textual data may constrain performance in less-annotated or novel datasets.

## 5. Conclusion and Suggestions on large language models in bioinformatics

### 5.1 Summary of large language models in bioinformatics

Large language models (LLMs) have catalyzed transformative progress across biological disciplines, including genomics, transcriptomics, proteomics, drug discovery, and single-cell analysis. These models, trained on vast datasets, address challenges like the sparsity, high dimensionality, and heterogeneity of biological data while capturing the complexity of sequence relationships. Tokenization methods are pivotal, converting sequences into manageable formats, such as, for genomics and transcriptomics, k-mer encoding is prevalent, segmenting DNA/RNA sequences into overlapping units. In proteomics, amino acid residue-based tokenization captures protein structure and function. These preprocessing strategies enable LLMs to interpret biological language effectively.

Representation learning allows LLMs to uncover contextual and hierarchical relationships within biological data, forming the basis for various downstream applications. These tasks can be grouped into four primary categories: 1) Classification/Prediction Tasks: Examples include identifying functional genomic elements (e.g., promoters, enhancers), predicting protein structures and interactions, and cell type annotation in single-cell data. 2) Generation Tasks: LLMs can create biologically relevant sequences, such as gene expression imputation and synthetic DNA, RNA, or protein sequences, aiding in vaccine development or enzyme engineering. 3) Interaction Tasks: These involve modeling interactions like drug-target binding, cell-cell interaction, protein-protein interactions, or cross-omics relationships (e.g., gene expression to protein abundance). 4) Transfer Learning Tasks: Pretrained LLMs, such as DNABERT and scGPT, are fine-tuned for specific applications, including single-cell data annotation or predicting RNA modifications like N6-methyladenosine sites. Despite their capabilities, challenges persist. Biological data often exhibit sparsity, as seen in single-cell and spatial transcriptomics, and irregularity due to sequencing errors or noise. To address this, LLMs must effectively integrate multi-modal data, balance computational efficiency, and ensure interpretability of their outputs. As foundational models evolve, their ability to unify diverse biological datasets into a single framework for prediction, generation, interaction, and transfer learning tasks will continue to reshape our understanding and applications of biological systems.

### 5.2 Guidance on how to use and develop LLMs in practice

**Figure 5.**
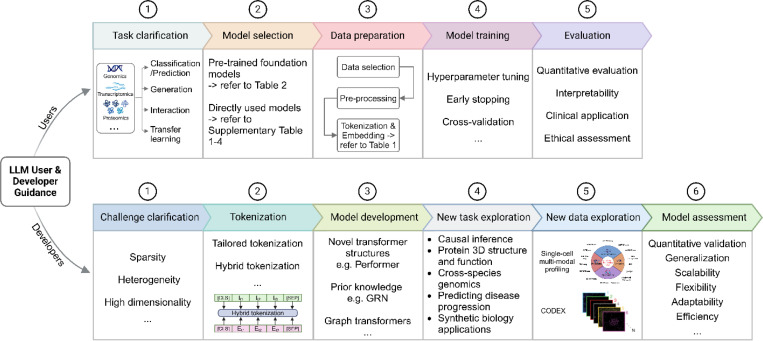
Guidance for LLM users and developers on how to use and develop LLM in practice. Guidance for LLM users includes steps such as clarifying the task, selecting an appropriate model, preparing the dataset, training the model, and evaluating its performance. For LLM developers, the focus involves identifying domain-specific challenges, designing tokenization strategies, advancing model architectures, exploring novel tasks and data types, and assessing model capabilities comprehensively.

Large Language Models offer immense potential in bioinformatics and other fields, but their effective utilization and development require distinct approaches for end-users and developers (Figure 5).

For LLM users, the process begins by clearly defining the research domain and task, specifying the relevant omics level (e.g., genomics, transcriptomics, proteomics) and identifying whether the objective involves classification or prediction, generation, interaction, or transfer learning. A well-defined objective streamlines the selection of appropriate models and workflows. Next, users should choose models pretrained on data relevant to their domain, as detailed in Table 2, which includes information on foundation models, their training data types, and availability. For instance, DNABERT is ideal for genomics tasks, while scGPT is tailored for single-cell analysis. Additionally, users must assess computational requirements and ensure compatibility with their dataset size and complexity. Proper data preparation is critical, including aligning data with model requirements, addressing missing values, and incorporating metadata like cell types or genomic regions. Table 1 provides common tokenization methods for reference. To leverage transfer learning, users can fine-tune foundation models listed in Table 2 for their specific dataset, optimizing performance through hyperparameter tuning, early stopping, and cross-validation. Alternatively, users can utilize predeveloped models listed in Supplementary Tables 1–4 for similar tasks to obtain results directly. Finally, rigorous evaluation using metrics like accuracy, precision, and recall is essential, complemented by interpretation tools such as attention maps or feature embeddings to extract meaningful biological insights (Figure 5).

For LLM developers, it is essential to **first** understand domain-specific challenges to address issues like sparsity, heterogeneity, and high dimensionality. For example, single-cell and spatial transcriptomics datasets often suffer from sparsity and noise, necessitating innovative solutions in model architecture. **Second,** developers should choose or develop tokenization strategies tailored to biological data. For instance, k-mer encoding works well for DNA/RNA sequences, while gene ranking-based tokenization is effective for scRNA-seq data. Exploring hybrid tokenization can enhance cross-modal understanding. **Third,** in model development, developers should employ or design novel transformer structures. For example, scBERT utilizes Performer to improve scalability. Incorporating knowledge-based information into model training can further enhance performance. For instance, GeneCompass integrates four types of biological prior knowledge including GRNs, promoter information, gene family annotation, and gene co-expression relationships, making it versatile for various gene-related tasks. Similarly, basic protein language models, which are often limited to MSA and protein sequences, can be improved by incorporating additional modalities like 3D structural data. This can be achieved by converting such modalities into sequence formats or integrating large models to collectively capture multi-modal information using fusion techniques. Moreover, combining Graph Neural Networks (GNNs) with transformers has led to significant advancements. For example, scMoFormer constructs cell-gene, gene-gene, protein-protein, and gene-protein graphs for multi-omics predictions, while DeepMAPS uses cell-gene graphs to estimate gene importance. GNNs excel in capturing local interactions, while transformers effectively model long-range dependencies, enabling comprehensive representations of intricate relationships in single-cell data. **Fourth,** novel tasks that can be explored in developing LLMs for bioinformatics include causal inference in multi-omics, such as determining how DNA variations influence mRNA abundance or protein expression. Spatial transcriptomics interpretation can model cell spatial organization within tissues. Epigenetic modulation prediction focuses on regulatory roles of histone modifications, DNA methylation, or chromatin accessibility. Synthetic biology applications can involve generating optimized gene or protein sequences, while cross-species genomics identifies conserved functional genomic elements. These tasks exemplify how LLMs can tackle emerging challenges in biological research. **Fifth,** developers should expand LLMs to accommodate emerging data types, such as CODEX imaging data and long-read sequencing data, which bring unique challenges in terms of data structure, preprocessing, and representation. **Lastly,** validation, application, and interpretability should be prioritized. Developers should not only evaluate models on specific tasks but also ensure that foundational challenges, such as the impact of sparsity in scRNA-seq data on cell type annotation performance, are fully addressed to enhance the robustness and utility of the models (Figure 5).

## Supplementary Material

Supplement 1

## Figures and Tables

**Table 1.** Tokenization methods for different types of data

Application area	Data type	Method	Example
Genomics/Transcriptomics	DNA/RNA sequence	One-hot encoding Fixed-length k-mers Special ‘[IND]’ token	RNA-FM, RNA-MSM DNABERT, Nucleotide Transformer, DNABERT-2, DNAGPT, RNABERT RNAErnie
Proteomics	MSAs/Protein sequences Biomedical text cDNA	Single Amino Acid Tokenization WordPiece Single condo Tokenization	MSA Transformer/TAPE, ESM-1b, ProtTrans, Progen ProtST CaLM
Drug discovery	Simplified Molecular-Input Line-Entry system (SMILES)	Random token SmilesTokenizer Graph VQ-VAE fingerprint	K-BERT ChemBERTa, ChemBERTa-2, MolGPT Mole-BERT SMILES-BERT
Single-cell analysis	Expression profiles	Gene expression Ranking Binning Gene set/Pathway tokens Patches Gene value projection Cell tokens	Geneformer, tGPT, iSEEEK scBERT, scGPT, scFormer, CellLM, BioFormers, CancerFoundation TOSICA CIForm, scTranSort, scCLIP scTranslator, scFounfation, scMulan, scGREAT CellPLM, ScRAT, mcBERT

**Table 2.** Foundation models in bioinformatics

Application area	Model	Architecture	Pre-training Data	Code available
	GPN	Transformer-based	Reference genomes from 8 species	https://github.com/songlab-cal/gpn
	Nucleotide Transformer	Transformer-based	3.2 billion nucleotides in GRCh38/hg38 reference assembly, 20.5 trillion nucleotides including 125 million mutations (111 million SNPs, 14 million indels), and 174 billion nucleotides from 850 species	https://github.com/instadeepai/nucleotide-transformer
	DNABERT	BERT-based	2.75 billion nucleotide based human genome dataset	https://github.com/jerryji1993/DNABERT
	DNABERT-2	BERT-based	2.75 billion nucleotide based human genome dataset and 32.49 billion nucleotide bases from 135 species, spread across 6 categories	https://github.com/MAGICS-LAB/DNABERT_2
Genomics	MoDNA	BERT-based	Same as Nucleotide Transformer	https://github.com/uta-smile/MoDNA
	GROVER	BERT-based	Homo sapiens (human) genome assembly GRCh37 (hg19)	https://github.com/rowanz/grover
	MuLan-Methyl	BERT-based	3 main types of DNA methylation sites (6mA, 4mC, and 5hmC) across 12 genomes, in total 250,599 positive samples	https://github.com/husonlab/mulan-methyl
	iDNA-ABF	BERT-based	Same as MuLan-Methyl	https://github.com/FakeEnd/iDNA_ABF
	iDNA-ABT	BERT-based	Same as MuLan-Methyl	https://github.com/YUYING07/iDNA_ABT
	DNAGPT	GPT-based	Reference genomes from the Ensembl database include 3 billion bps, with a total of 1,594,129,992 bps across 9 species	https://github.com/TencentAILabHealthcare/DNAGPT

	RNABERT	BERT-based	76 237 human-derived small ncRNAs from RNAcentral	https://github.com/mana438/RNABERT
	RNA-FM	BERT-based	About 27 million ncRNA sequences across 47 different databases	https://github.com/ml4bio/RNA-FM
	RNA-MSM	BERT-based	4069 RNA families from rfam	https://github.com/yikunpku/RNA-MSM
Transcriptomics	SpliceBERT	BERT-based	2 million sequences and approximately covering 65 billion nucleotides of 72 vertebrates from UCSC genome browser	https://github.com/biomed-AI/SpliceBERT
	UNI-RNA	BERT-based	23 million ncRNA sequences obtained from the RNAcentral database	https://github.com/ComDec/unirna-tools
	3UTRBERT	BERT-based	108,573 unique mRNA transcripts from the GENCODE and each contains 3,754 nucleotides (median 3048 nts) on average.	https://github.com/yangyn533/3UTRBERT
	UTR-LM	BERT-based	214,349 unlabeled 5′ UTR sequences from Ensembl across 5 species	https://github.com/a96123155/UTR-LM
	RNAErnie	Transformer- based	23 million ncRNA sequences obtained from the RNAcentral database	https://github.com/CatIIIIIIII/RNAErnie

	TAPE	Transformer-based	31 million protein sequences from Pfam	https://github.com/songlab-cal/tape
	ESM-1b	Transformer-based	250 million protein sequences from UniRef50	https://github.com/facebookresearch/esm
Proteomics	ProtTrans	Transformer-XL, XLNet, BERT, Albert, Electra, T5	About 2.3 billion protein sequences from UniRef and BFD	https://github.com/agemagician/ProtTrans
	ProtGPT2	GPT-based	50 million protein sequences from UniRef50	https://huggingface.co/docs/transformers/main_classes/trainer
	ProteinBERT	BERT-based	106 million protein sequences with GO annotations from UniRef50	https://github.com/nadavbra/protein_bert
	KeAP	BERT-based	5 million Triplet in the format of (Protein, Relation, Attribute) with nearly 600k protein, 50k attribute terms, and 31 relation terms included	https://github.com/RL4M/KeAP
	CaLM	Transformer-based	9,858,385 cDNA sequences of seven model organisms	https://github.com/oxpig/CaLM

	SMILES-BERT	BERT-based	Two datasets from NCATS (NIH) and 128 datasets from PubChem	https://github.com/uta-smile/SMILES-BERT
	ChemBERTa	BERT-based	77 million unique SMILES	https://github.com/seyonechithrananda/bert-loves-chemistry
	K-BERT	BERT-based	Book review dataset contains 20,000 positive and 20,000 negative reviews collected from Douban	https://github.com/autoliuweijie/K-BERT
Drug discovery	Mole-BERT	BERT-based	2 million molecules	https://github.com/junxia97/Mole-BERT
	MolGPT	GPT-based	Datasets from MOSES and GuacaMol	https://github.com/devalab/molgpt
	ProtBERT	BERT-based	Datasets from Uniref50, UniRef100 and BFD	https://github.com/agemagician/ProtTrans/
	DeepDDS	BERT-based	Datasets from NCI-ALMANAC	https://github.com/sorachel/DFFNDDS
	SynerGPT	GPT-based	Datasets from DrugCombDB	*Code will be made available upon publication*

	scBERT	BERT-based	1,126,580 cells from 209 datasets across 74 tissues and 451,513 cells from four sequencing platforms	https://github.com/TencentAILabHealthcare/scBERT
	scGPT	GPT-based	33 million human cells from the CellXGene collection	https://github.com/bowang-lab/scGPT
	Geneformer	BERT-based	29.9 million human single-cell transcriptomes	https://huggingface.co/ctheodoris/Geneformer
	scFoundation	BERT-based	About 50 million human single-cell transcriptomic profiles	https://github.com/biomap-research/scFoundation
	tGPT	GPT-based	22.3 million single-cell transcriptomes	https://github.com/deeplearningplus/tGPT
Single-cell analysis	GeneCompass	BERT-based	over 120 million single-cell transcriptomes from humans and mice	https://github.com/xCompass-AI/GeneCompass
	scMulan	GPT-based	More than 10 million manually annotated single-cell RNA-seq data	https://github.com/SuperBianC/scMulan
	UCE	BERT-based	300 datasets from the CellXGene corpus includes over 36 million cells, 1,000+ cell types, dozens of tissues, and eight species	https://github.com/snap-stanford/uce
	scPRINT	BERT-based	More than 50M cells from theCellXGene database	https://github.com/cantinilab/scPRINT
	CancerFoundation	BERT-based	50 million cells with roughly a quarter being tumor cells	https://github.com/BoevaLab/CancerFoundation
	Nicheformer	BERT-based	57 million dissociated and 53 million spatially resolved cells across 73 tissues from both human and mouse	https://github.com/theislab/nicheformer

**Table 3.** Large language models for downstream tasks in bioinformatics

Input data	Biological tasks	Models
	Genome-wide variant effects prediction	DNABERT, DNABERT-2, GPN, Nucleotide Transformer
	DNA cis-regulatory regions prediction	DNABERT, DNABERT-2, BERT-Promoter, iEnhancer-BERT, Nucleotide Transformer
DNA sequence	DNA-protein interaction prediction	DNABERT, DNABERT-2, TFBert, GROVER, and MoDNA
	DNA methylation (6mA,4mC 5hmC) prediction	BERT6mA, iDNA-ABF, iDNA-ABT, and MuLan-Methyl
	RNA splice sites prediction from DNA sequence	DNABERT, DNABERT-2

	RNA 2D/3D structure prediction	RNA-FM, RNA-MSM, and RNA-FM
	RNA structural alignment, RNA family clustering	RNABERT
	RNA splice sites prediction from RNA sequence	SpliceBERT
	RNA N7-Methylguanosine modification prediction	BERT-m7G
RNA sequence	RNA 2’-O-methylation Modifications prediction	Bert2Ome
	Multiple types of RNA modifications prediction	Rm-LR
	Predicting the association between miRNA, lncRNA and disease	BertNDA
	Identifying lncRNAs	LncCat
	Protein expression and mRNA degradation prediction	CodonBERT

Protein sequences MSAs Gene ontology annotations Triplets of protein-relation-attribute Protein property descriptions cDNA sequences	Secondary structure and contact prediction	MSA Transformer, ProtTrans, SPRoBERTa, TAPE, KeAP
	Protein sequence generation	ProGen, ProtGPT2
	Protein function prediction	SPRoBERTa, ProtST, PromptProtein, CaLM
	Major PTMs prediction	ProteinBERT
	Evolution and mutation prediction	SPRoBERTa, UniRep, ESM-1b, TAPE, PLMsearch, DHR
	Biophysical properties prediction	TAPE, PromptProtein
	Protein-protein interaction and binding affinity prediction	KeAP
	Antigen-Receptor binding prediction	MHCRoBERTa, BERTMHC, TCR-BERT, SC-AIR-BERT, Antiformer
	Antigen-Antibody binding prediction	AbLang, AntiBERTa, EATLM

Molecular SMILES	Predicting Molecular Properties	SMILES-BERT, ChemBERTa, K-BERT
	Generating Molecules	MolGPT
Molecular graphs	Predicting Molecular Properties	MOLE-BERT
Molecular fingerprints and protein sequences	Predicting Drug-Target Interaction	TransDTI, FG-BERT
Molecular SMILES and protein sequences	Predicting Synergistic Effects	SynerGPT, C2P2

scRNA-seq data	Cell clustering	tGPT, scFoundation, UCE, iSEEEK, CellPLM, BioFormers, mcBERT
	Cell type annotation	scBERT, scGPT, CIForm, TOSICA, scTransSort, TransCluster, Geneformer, GeneCompass, scMulan, CellLM, CellPLM, scPRINT
	New cell type identification	scBERT, TOSICA, UCE
	Batch effect removal	scBERT, scGPT, CIForm, TOSICA, Geneformer, scMulan, iSEEEK, scPRINT, CancerFoundation, mcBERT
	Trajectory inference/Pseudotime analysis	tGPT, scMVP, iSEEEK
	Drug response/sensitivity prediction	scFoundation, CellLM, CancerFoundation

	Gene network inference	scGPT, Geneformer, GeneCompass, iSEEEK, scGREAT, BioFormers, scPRINT
	Gene perturbation prediction	scGPT, scFoundation, GeneCompass, CellPLM, BioFormers
	Gene expression prediction	scGPT, scMVP, scFoundation, GeneCompass, CellPLM, BioFormers
	cis-regulatory element identification	scMVP
	Drug dose-response prediction, Gene dosage sensitivity prediction	GeneCompass

scMuti-omics data	Single-cell multi-omics integration	scGPT, scMVP, DeepMAPS, scCLIP
	Biological network inference	DeepMAPS
	Cell-cell communications	
	Translating gene expression to protein abundance	scTranslator, scMoFormer
	Single-cell multimodal prediction	scMoFormer
	Integrative regulatory inference	scTranslator

Single-cell spatial transcriptomics data	Spatial transcriptomics imputation	CellPLM, Nicheformer, SpaFormer
	Spatial label prediction	
	Spatial neighborhood density prediction	Nicheformer
	Spatial neighborhood composition prediction	
