# Supplementary material for: Advancing bioinformatics with large language models: components, applications and perspectives
Source: ArXiv. 2025 Jan 31:arXiv:2401.04155v2. Originally published 2024 Jan 8. Preprint. [Version 2] (PMC10802675)
Supplement: Supplement 1 [file NIHPP2401.04155V2-supplement-1.pdf]

Supplementary figures

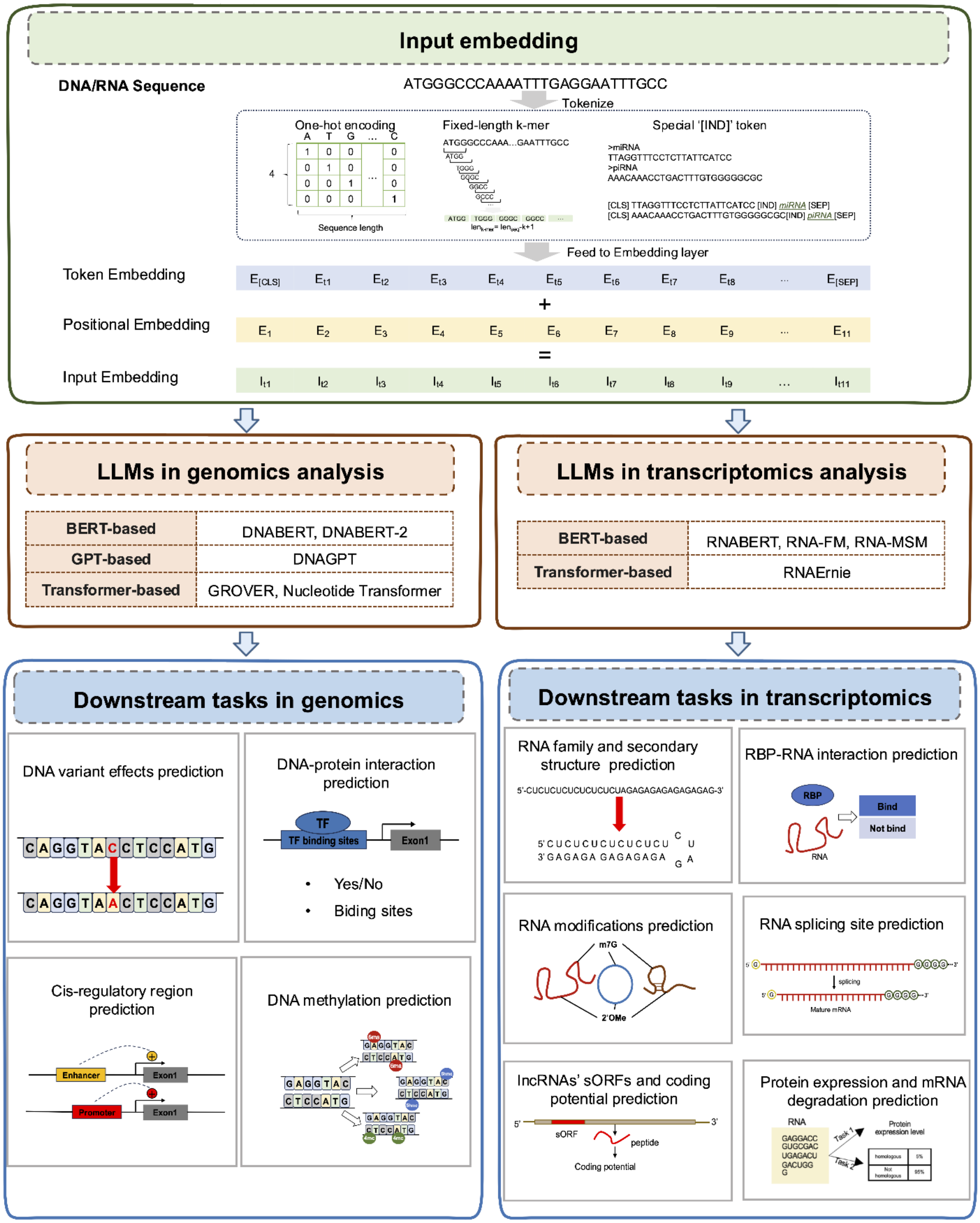

Supplementary figure 1. Applications of large language models in genomics and transcriptomics. DNA language models take DNA sequence as input, use transformer, BERT,

GPT models to solve multiple biological tasks, including genome-wide variant effects prediction, DNA cis-regulatory regions prediction, DNA-protein interaction prediction, DNA methylation (6mA,4mC 5hmC) prediction, splice sites prediction from DNA sequence. The RNA language models take RNA sequences as input, use transformer, BERT, GPT models to solve multiple biological tasks, including RNA 2D/3D structure prediction, RNA structural alignment,, RNA family clustering, RNA splice sites prediction from RNA sequence, RNA N7-methylguanosine modification prediction, RNA 2'-O-methylation modifications prediction, multiple types of RNA modifications prediction, predicting the association between miRNA, lncRNA and disease, identifying lncRNAs, lncRNAs' coding potential prediction, protein expression and mRNA degradation prediction.

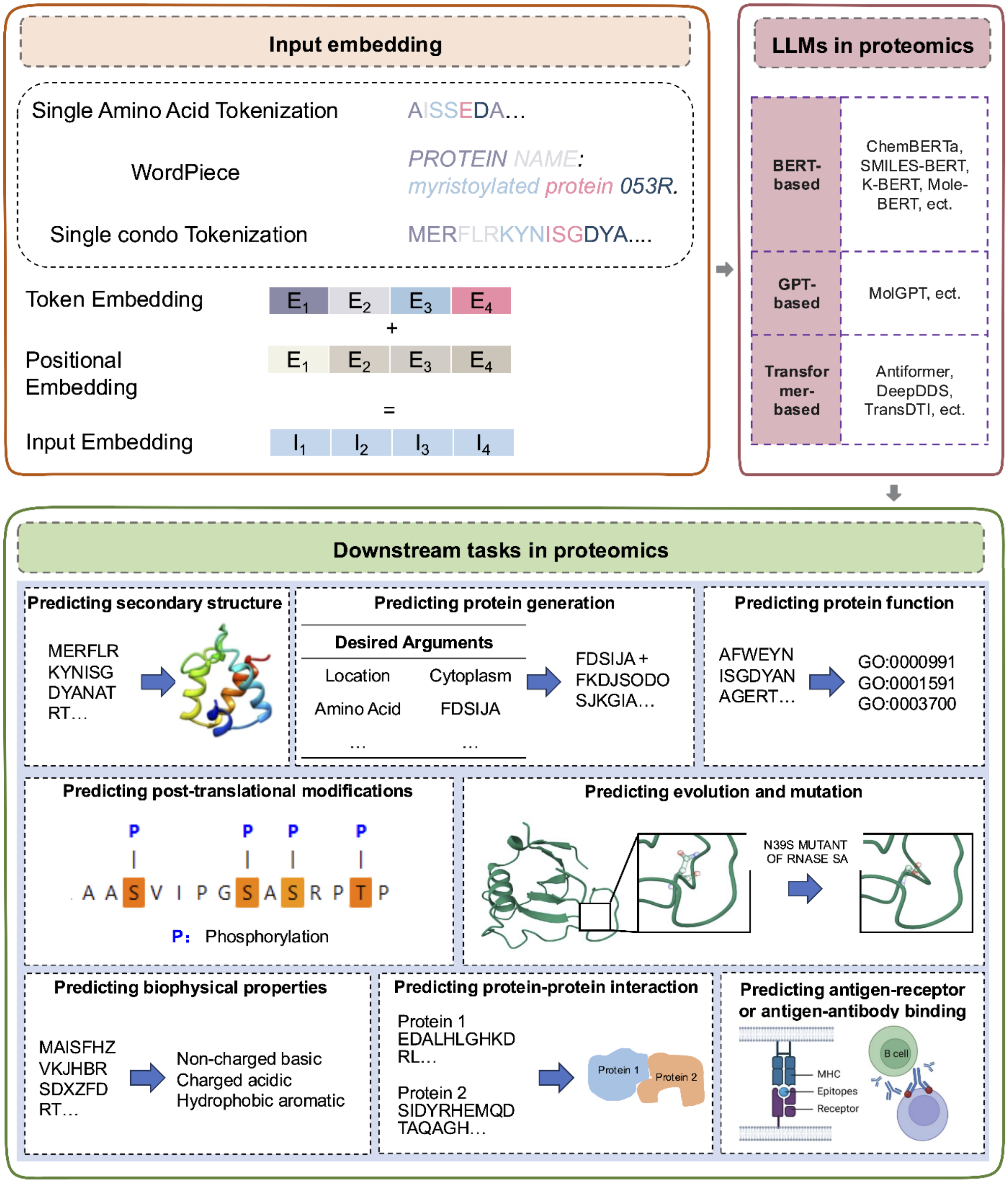

Supplementary figure 2. Applications of large language models in proteomics. The protein language models take multiple sequence alignment, protein sequence, gene ontology and protein-relation-attribute as input, use transformer, BERT, GPT models to solve multiple biological tasks, including predicting secondary structure, predicting protein generation, predicting protein function,

predicting post-translational modifications, predicting evolution and mutation, predicting biophysical properties, predicting protein-protein interaction and predicting antigen-receptor or antigen-antibody binding.

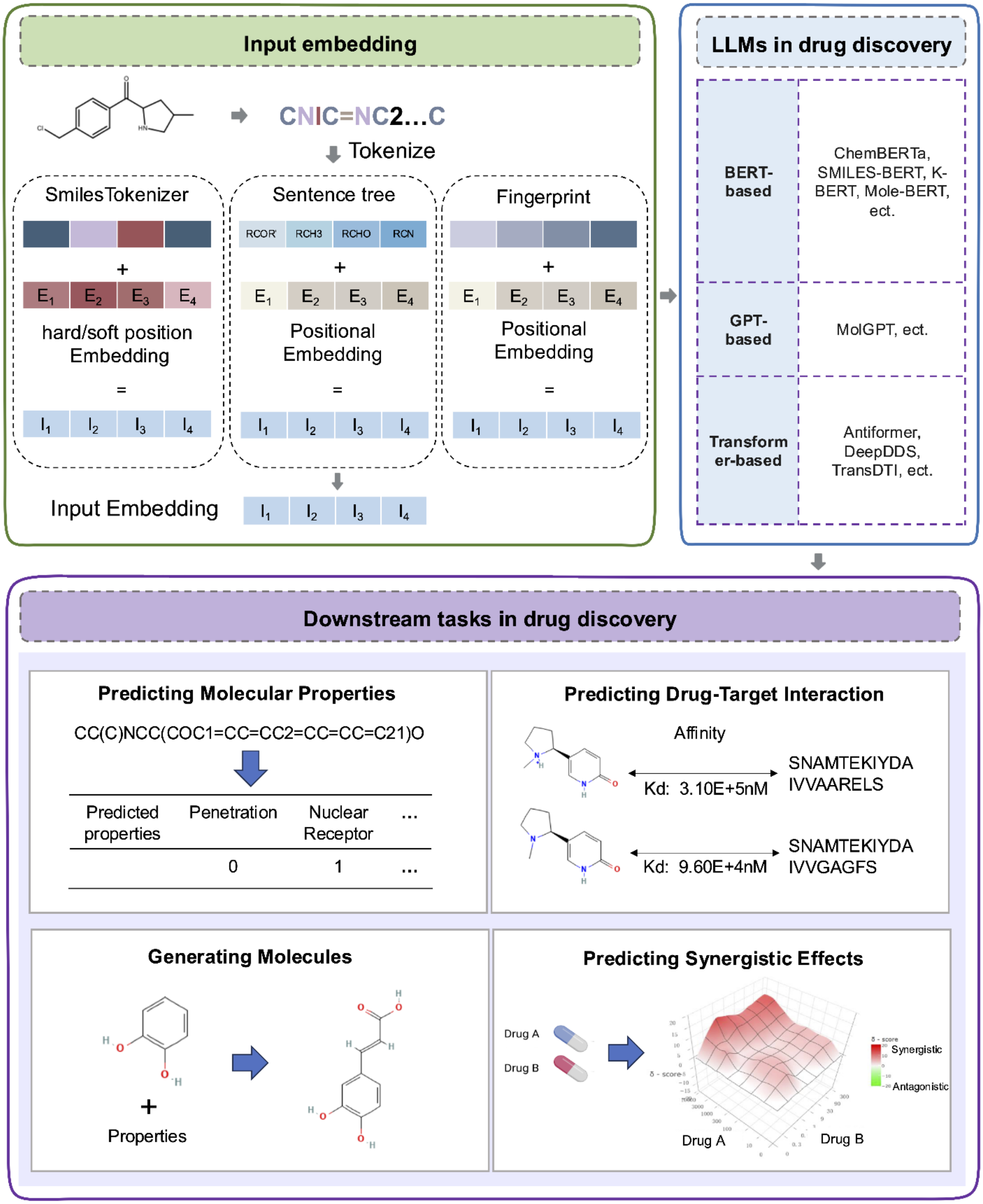

Supplementary figure 3. Applications of large language models in drug discovery. The language models for drug discovery take molecular SMILES, protein sequence, molecular fingerprints and molecular graphs as input, use transformer, BERT, GPT models to solve multiple

biological tasks, including predicting molecular properties, predicting drug-target interaction, generating molecules and predicting synergistic effects.

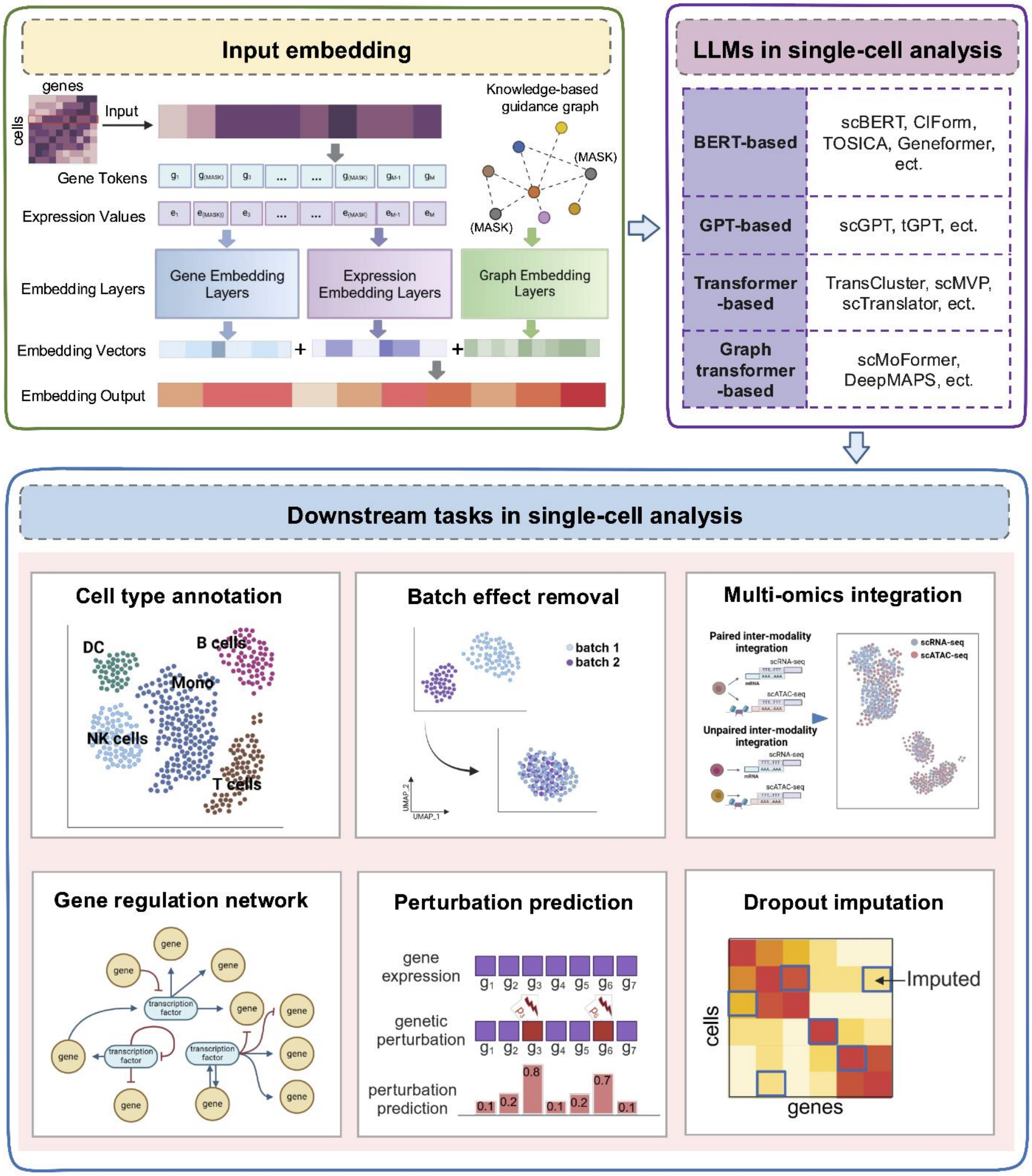

Supplementary figure 4. Applications of large language models in single-cell analysis. The single-cell language models take gene expression or single-cell multi-omics data as input, use transformer, BERT, GPT models to solve multiple biological tasks, including cell type annotation, batch effect removal, multi-omics integration, gene regulation network inference perturbation prediction, dropout imputation.

Supplementary Tables

Supplementary Table 1. Detailed information of large language models for genomic and transcriptomic tasks

Application area	Models	Ref	Publication time	Parameters	Architecture	Fine-tuning datasets			Downstream tasks
						Data type	Source	Size	
DNA sequence language model	DNABERT	[1]	Aug 2021	12 transformer layers with 768 hidden units and 12 attention heads in each layer	BERT-based	DNA sequence	Human TATA and non-TATA promoters of 10 000 bp length[2] and ChIP-seq dataset [3]	3,065 human TATA and 26,533 non-TATA promoter-containing sequences and 690 ChIP-seq dataset covers 161 transcription factor binding profiles in 91 human cell lines	Transcription factor binding sites prediction
							DNA sequence	-	Motif analysis
							Assembly GRCh38 FASTA file [4]	10,000 donor, acceptor, and non-splice site sequences	Splice donor and acceptor sites prediction
							dbSNP release 153 [5]	700 million short genetic variants	Identifying effects of genetic variants
	DNABERT-2	[6]	July 2023	batch size is 32, warmup step is 50, and weight decay is 0.01	BERT-based	DNA sequence	TATA and non-TATA promoters downloaded from Eukaryotic Promoter Database (EPDnew) [2]	3,065 human TATA and 26,533 non-TATA promoter-containing sequences	Promoter detection and core promoter detection
							ChIP-seq datasets [7]	161 TF binding profiles in 91 human cell lines(human) and 78 mouse ENCODE ChIP-seq data	Transcription factor binding site prediction
							Ensembl GRCh38 human reference genome [4]	10,000 splice donors, acceptors, and non-splice site sequences.	Splice site prediction
							Histone modification (Yeast)	H3, H3K14ac, H3K36me3, H3K4me1, H3K4me2, H3K4me3, H3K79me3, H3K9ac, H4, H4ac	Epigenetic marks prediction
							SARS_CoV_2 variants [8]	9 types of SARS_CoV_2 variants, including *Alpha, Beta,Delta, Eta, Gamma, Iota, Kappa, Lambda and Zeta.*	Covid variant prediction
	Nucleotide	[9]	Jan 2023	2 hidden layers	Transfor	DNA	Annotated DNA sequence	90,000 sequences annotated by	Detect known genomic

	Transformer				mer-based	sequence	[10]	Ensembl (“5’ UTR”, “3’ UTR”, “exon”, “intron”, “enhancer”, “promoter”, “CTCF binding site”, “open chromatin”, and “transcription factor binding sites”.)	elements
							DNA sequence with SNP [11]	Independent dataset of genetically diverse human genomes, originating from 7 different meta-populations	Detect human genetic variation
							1000 Genomes Project SNPs [12]	chromosome 22 sequence with 17 variant categories (e.g. stop gained, missense, intergenic)	Predict the impact of mutations
	DNAGPT	[13]	July 2023	12 layers of transformer blocks based on unidirectional attention, with each layer containing 12 attention heads and a hidden layer size of 768	GPT-based	DNA sequence	DNA sequence from DeepGSR [14]	20,933, 18,693, 12,082, and 27,203 true polyadenylation signals data; and 28,244, 25,205, 17,558, and 30,283 true translation initiation sites for human, mouse, bovine, and fruit fly, respectively which are used as ground-truth, non-genomic signals and regions sequences from the genome sequences and combined them with the true cases	Genomic signals and regions prediction
							DNA sequence from Xpresso [15]	18,377 and 21,856 promoters as well as the mRNA half-lives in human and mouse respectively and held out 1000 cases in each specie	mRNA expression level prediction
	GROVER	[16]	July 2023	12 transformer layers, 5,000 embeddings	BERT-based	DNA sequence	CTCF ChIP-seq data [17]	~85,000 binding motifs, only ~32,000 are indeed bound by CTCF	Protein-DNA binding prediction
	GPN	[18]	BioRxiv posted April 2023	25 convolutional blocks with a feed-forward layer, 512embedding sizes of the pre-trianed foundation model	BERT-based	DNA sequence	DNA sequence	-	DNA motifs predictions
							1001 Genomes Project [12]	10 million SNPs	Variant effect prediction

	BERT-Promoter	[19]	Aug 2022	BERT model included 12 layers, 768-hidden, 12 heads, and 110,000,000 parameters	BERT-based	DNA sequence	ChIP-chip data, gSELEX peaks, ChIP-exo plus RNA-seq [20, 21]	3382 promoters (1591 strong promoter samples and 1791 weak promoter samples) and 3382 non-promoters	DNA promoter prediction
	TFBert	[22]	Mar 2023	12-layer encoder	BERT-based	DNA sequence	ChIP-seq datasets [7]	690 ChIP-seq dataset contains a training set (80%) and a corresponding test set (20%)	DNA–protein binding sites prediction
	MoDNA	[23]	Aug 2022	-	BERT-based	DNA sequence	Same experiment data with DNABERT [2, 3]	3,065 human TATA and 26,533 non-TATA promoter-containing sequences and 690 ChIP-seq dataset covers 161 transcription factor binding profiles in 91 human cell lines	Promoter Prediction
							CHIP-Seq datasets [3]	690 CHIP-Seq datasets of uniform TFBS contains 161 TFs covering 91 human cell types	Transcription Factor Binding Sites Prediction
	iEnhancer-BERT	[24]	Aug 2022	12-layer transformer architecture with simple fine-tuning	BERT-based	DNA sequence	15 chromatin states of 9 cell types [25]	2968 samples including 1484 non-enhancers, 742 strong enhancers and 742 weak enhancers	Identifying Enhancers and Their Strength
	BERT6mA	[26]	Mar 2022	The hidden size of the LSTM unit is set to 128.DNA sequence embedding with Word2vec.	BERT-based	DNA sequence	Nuclei purification, MNase-seq and ChIP-seq [27-31]	6mA and non6mA data in 11 species including Arabidopsis thaliana (31873 6mAs and non-6mAs), Caenorhabditis elegans (79616 mAs and non-6mAs), Casuarina equisetifpolia (6066 6mAs and non-6mAs), Drosophila melanogaster (11 191 6mAs and non-6mAs), Fragaria vesca (3102 6mAs and non-6mAs), H. sapiens (18 335 6mAs and non-6mAs), Rosa chinensis (599 6mAs and non-6mAs), Saccharomyces cerevisiae (37866mAs and non-6mAs), Thermus thermophilus (107 600 6mAs and non-6mAs), Ts. SUP5–1 (3379	DNA N6-methyladenine site prediction

								6mAs and non6mAs) and Xoc. BLS256 (17 215 6mAs and non-6mAs)	
	iDNA-ABF	[32]	Oct 2022	12 transformer layers with 768 hidden units and 12 attention heads in each layer	BERT-based	DNA sequence	ChIP-seq data, ATAC-seq data, and histone modifications (HM) data of three human cell lines [33, 34], and DNA methylation dataset from the iDNA-MS [35]	3 main types of DNA methylation sites (6mA,4mC, and 5hmC) across 12 genomes (1 bacteria and 11 eukaryotes), in total 250,599 positive samples	DNA 6mA, 4mC, and 5hmC prediction
							DNA sequence	-	Motifs analysis
	iDNA-ABT	[36]	Sep 2021	12 transformer layers with 12 attention heads in each layer.	BERT-based	DNA sequence	ChIP-seq data, ATAC-seq data, and histone modifications (HM) data of three human cell lines [33, 34] , and DNA methylation dataset from the iDNA-MS [35]	3 main types of DNA methylation sites (6mA,4mC, and 5hmC) across 12 genomes (1 bacteria and 11 eukaryotes), in total 250,599 positive samples	DNA 6mA, 4mC, and 5hmC prediction
							DNA sequence	-	Motifs analysis
	MuLan-Methyl	[37]	July 2023	12 layers in the encoder stack, 768 hidden units for feed-forward networks, and 12 attention heads.	BERT-based	DNA sequence	ChIP-seq data, ATAC-seq data, and histone modifications (HM) data of three human cell lines [33, 34] , and DNA methylation dataset from the iDNA-MS [35]	3 main types of DNA methylation sites (6mA,4mC, and 5hmC) across 12 genomes (1 bacteria and 11 eukaryotes), in total 250,599 positive samples	DNA 6mA, 4mC, and 5hmC prediction
RNA sequence language model	RNA-MSM	[38]	Nov 2023	12 attention heads with embedding size of 768	BERT-based	RNA sequence	RNA secondary structure and three-dimensional RNA structures [39]	The training, validation, and test sets have 405, 40, and 70 RNAs.	RNA secondary structure prediction
							RNA secondary structure and three-dimensional RNA structures [39]	The training, validation, and test sets have 405, 40, and 70 RNAs.	RNA solvent accessibility prediction
	RNA-FM	[40]	Arxiv posted Apr 2022	12 transformer-based bidirectional encoder blocks and 640 embedding	BERT-based	RNA sequence	RNA secondary structure [41, 42]	37149 structures from 8 RNA types of RNAStralign and 3975 RNA structures from 10 RNA types of ArchiveII	RNA secondary structure prediction
							RNA secondary structure [41, 42]	37149 structures from 8 RNA types of RNAStralign and 3975 RNA structures from 10 RNA types of ArchiveII	RNA 3D closeness prediction

							whole genome of Severe acute respiratory syndrome coronavirus 2 (SARS-CoV-2) [43]	Whole genome	SARS-CoV-2 genome structure and evolution prediction
							In vivo RNA secondary structure profiles for RNA-protein interaction [44]	-	Protein-RNA interaction prediction
							Human 5'UTR library [45]	83,919 5'UTRs of 75 different lengths and their corresponding mean ribosome loadings	mRNA 5' UTR-based mean ribosome loading prediction
	RNABERT	[46]	Feb 2022	6 hidden layers of BERT, an embedding layer, one bidrectional-LSTM unit, two dense layers one with ReLU activation and a softmax output layer of LSTM	BERT-based	RNA sequence	RNA (ncRNA) families from RFam database[46]	31 RNA families	classifying RNA families RNA secondary structure prediction
	SpliceBERT	[47]	Mar 2024	6 transformer encoder layers, 512 hidden layer and 16 attention heads	BERT-based	RNA sequence	Reference genomes in fasta [48]	The pre-mRNA sequences from 72 vertebrate genomes for pre-training	Estimating splice sites
	BERT-m7G	[49]	Aug 2021	-	BERT-based	RNA sequence	RNA sequence with N7-methylguanosine sites and RNA sequence without N7-methylguanosine sites AlkAniline-Seq, MeRIP-seq, and miCLIP-seq [50]	741 RNA sequences with N7-methylguanosine sites and 741 RNA sequences without N7-methylguanosine sites	RNA N7-methylguanosine sites prediction
	M6A-BERT-Stacking	[51]	March 2023	12 transformer layers with 12 attention heads in each layer.	BERT-based	RNA sequence	RNA sequence with m6A sites and RNA sequence without m6A sites identified from MeRIP, m6A-seq, PA-m6A-seq, and miCLIP [52]	11 datasets including 3000~ 16000 RNA sequences for each dataset	RNA m6A sites prediction
	Bert2Ome	[53]	May 2023	16 heads, 12 layers, and 1024 hidden units	BERT-based	RNA sequence	2-O-methylation modification sites from RMBase database [54]	215 positive, 215 negative instances for the training part and 46 positive, 114 negative instances for the testing part.	RNA 2-O-methylation prediction
	Rm-LR	[55]	Sep 2023	6 transformer	BERT-	RNA	Transcriptomic-wide	20 different epi-transcriptome	Multiple types of RNA

				encoder layers, with a hidden layer size of 512 and 16 attention heads	based	sequence	profiling data derived from the MultiRM, GEO, RMBase, RADAR [54, 56-58]	profiles based on various base resolution techniques	modifications prediction
	BertNDA	[59]	Nov 2023	8 layers	BERT-based	RNA sequence	miRNA-disease associations, lncRNA-disease associations, and miRNA-lncRNA associations [60-63]	1000 positive pairs and 1000 negative pairs	ncRNA-Disease Association Prediction
	LncCat	[64]	Feb 2023	-	BERT-based	RNA sequence	lncRNAs and protein-coding transcripts of five species [10, 63, 65]	22960 coding transcripts and 21081 lncRNAs of human. 20707 coding transcripts and 10707 lncRNAs of mouse. 15891 coding transcripts and 4382 lncRNAs of zebrafish. 4693 coding transcripts and 5377 lncRNAs of wheat 20584 coding transcripts and 3897 lncRNAs of chicken	Identify lncRNA
	LSCPP-BERT	[66]	Dec 2023	4 identical layers and each layer is divided into two sublayers	BERT-based	RNA sequence	lncRNAs sequences from multispecies [67]	593251 plant lncRNAs sequences	lncRNA-sORFs coding potential prediction
	CodonBERT	[68]	Oct 2023	12 layers of bidirectional transformer encoders, Each transformer layer with 12 self-attention heads	BERT-based	RNA sequence	mRFP Expression dataset; [69] Fungal expression dataset; E. coli proteins dataset; mRNA stability dataset; Tc-Riboswitches dataset [70]	Experimental data for protein expression (2308 low expression proteins, 2067 medium expression proteins, and 1973 high expression proteins, respectively);	mRNA properties prediction
							SARS-CoV-2 Vaccine degradation dataset	-	Vaccine expression prediction
	RNA-TorsionBERT	[71]	Jun 2024	18 layer	BERT-based	RNA sequence	PDB structure and removed the structures from the nonredundant Training	4,267 structures with sequences from 11 to 508 nucleotides	RNA 3D structure prediction
	UNI-RNA	[72]	bioRxiv posted Jul 2023	-	BERT-based	RNA sequence	non-coding RNA sequences from RNAcentral, nucleic acid data from NCBI's database, and genomic data from repositories such as Genome Warehouse[73-75]	37,149 RNA structures 7,600 held-out real human 5'UTRs 3 million distinct UTR sequences	RNA secondary structure prediction RNA distance map prediction mRNA 5′-UTR mean ribosome load prediction Alternative

									polyadenylation isoform prediction<br>RNA splice site prediction<br>ncRNA classification<br>RNA modification prediction
	UTR-LM	[76]	Apr 2024	Six-layer transformer with 16 multi-head self-attention.	Transformer-based	RNA sequence	Unlabeled 5' UTR sequences from three sources: the Ensembl database	214,349 unlabeled 5' UTR sequences	Mean ribosome loading prediction.<br>mRNA expression level and translation efficiency prediction<br>Internal ribosome entry site identification<br>Attention-based motif detection
	3UTRBERT	[77]	Aug 2024	12 identical Transformer components， Each layer contained a multi-head self-attention module and a position-wise fully connected feed-forward layer	BERT-based	RNA sequence	3'UTR of human mRNAtranscript<br>eCLIP datasets<br>RNA localization data	108 573 unique mRNA transcripts<br>1000 samples RNA binding samples with1000 sequences<br>17 023 mRNAs	mRNA subcellular localization prediction
	RNAErnie	[78]	May 2024	12-layer transformer and a hidden state dimension of 768	Transformer-based	RNA sequence	non-coding RNA sequences from RNAcentral		RNA grouping<br>RNA sequence classification<br>RNA–RNA interaction prediction<br>RNA secondary structure prediction

References

1. Ji, Y., et al., *DNABERT: pre-trained Bidirectional Encoder Representations from Transformers model for DNA-language in genome.* Bioinformatics, 2021. **37**(15): p. 2112-2120.
2. Dreos, R., et al., *EPD and EPDnew, high-quality promoter resources in the next-generation sequencing era.* Nucleic acids research, 2013. **41**(D1): p. D157-D164.
3. Consortium, E.P., *An integrated encyclopedia of DNA elements in the human genome.* Nature, 2012. **489**(7414): p. 57.
4. Cunningham, F., et al., *Ensembl 2019.* Nucleic acids research, 2019. **47**(D1): p. D745-D751.
5. Sherry, S.T., et al., *dbSNP: the NCBI database of genetic variation.* Nucleic acids research, 2001. **29**(1): p. 308-311.
6. Zhou, Z., et al., *Dnabert-2: Efficient foundation model and benchmark for multi-species genome.* arXiv preprint arXiv:2306.15006, 2023.
7. Zeng, H., et al., *Convolutional neural network architectures for predicting DNA–protein binding.* Bioinformatics, 2016. **32**(12): p. i121-i127.
8. Chen, K., H. Zhao, and Y. Yang, *Capturing large genomic contexts for accurately predicting enhancer-promoter interactions.* Briefings in Bioinformatics, 2022. **23**(2): p. bbab577.
9. Dalla-Torre, H., et al., *The nucleotide transformer: Building and evaluating robust foundation models for human genomics.* bioRxiv, 2023: p. 2023.01. 11.523679.
10. Howe, K.L., et al., *Ensembl 2021.* Nucleic acids research, 2021. **49**(D1): p. D884-D891.
11. Bergström, A., et al., *Insights into human genetic variation and population history from 929 diverse genomes.* Science, 2020. **367**(6484): p. eaay5012.
12. Alonso-Blanco, C., et al., *1,135 genomes reveal the global pattern of polymorphism in Arabidopsis thaliana.* Cell, 2016. **166**(2): p. 481-491.
13. Zhang, D., et al., *DNAGPT: A Generalized Pretrained Tool for Multiple DNA Sequence Analysis Tasks.* bioRxiv, 2023: p. 2023.07. 11.548628.
14. Kalkatawi, M., et al., *DeepGSR: an optimized deep-learning structure for the recognition of genomic signals and regions.* Bioinformatics, 2019. **35**(7): p. 1125-1132.
15. Agarwal, V. and J. Shendure, *Predicting mRNA abundance directly from genomic sequence using deep convolutional neural networks.* Cell

reports, 2020. **31**(7).
16. Sanabria, M., et al., *DNA language model GROVER learns sequence context in the human genome.* Nature Machine Intelligence, 2024. **6**(8): p. 911-923.
17. de Souza, N., *The ENCODE project.* Nature methods, 2012. **9**(11): p. 1046-1046.
18. Benegas, G., S.S. Batra, and Y.S. Song, *DNA language models are powerful zero-shot predictors of genome-wide variant effects.* bioRxiv, 2022: p. 2022.08. 22.504706.
19. Le, N.Q.K., et al., *BERT-Promoter: An improved sequence-based predictor of DNA promoter using BERT pre-trained model and SHAP feature selection.* Computational Biology and Chemistry, 2022. **99**: p. 107732.
20. Gama-Castro, S., et al., *RegulonDB version 9.0: high-level integration of gene regulation, coexpression, motif clustering and beyond.* Nucleic acids research, 2016. **44**(D1): p. D133-D143.
21. Xiao, X., et al., *iPSW (2L)-PseKNC: A two-layer predictor for identifying promoters and their strength by hybrid features via pseudo K-tuple nucleotide composition.* Genomics, 2019. **111**(6): p. 1785-1793.
22. Luo, H., et al., *Improving language model of human genome for DNA–protein binding prediction based on task-specific pre-training.* Interdisciplinary Sciences: Computational Life Sciences, 2023. **15**(1): p. 32-43.
23. An, W., et al. *MoDNA: motif-oriented pre-training for DNA language model*. in *Proceedings of the 13th ACM International Conference on Bioinformatics, Computational Biology and Health Informatics*. 2022.
24. Luo, H., et al. *iEnhancer-BERT: A novel transfer learning architecture based on DNA-Language model for identifying enhancers and their strength*. in *International Conference on Intelligent Computing*. 2022. Springer.
25. Ernst, J., et al., *Mapping and analysis of chromatin state dynamics in nine human cell types.* Nature, 2011. **473**(7345): p. 43-49.
26. Tsukiyama, S., et al., *BERT6mA: prediction of DNA N6-methyladenine site using deep learning-based approaches.* Briefings in Bioinformatics, 2022. **23**(2): p. bbac053.
27. Xiao, C.-L., et al., *N6-methyladenine DNA modification in the human genome.* Molecular cell, 2018. **71**(2): p. 306-318. e7.
28. Ye, G., et al., *De novo genome assembly of the stress tolerant forest species Casuarina equisetifolia provides insight into secondary growth.* The Plant Journal, 2019. **97**(4): p. 779-794.
29. Ye, P., et al., *MethSMRT: an integrative database for DNA N6-methyladenine and N4-methylcytosine generated by single-molecular real-*

time sequencing. Nucleic acids research, 2016: p. gkw950.
30. Liu, Z.-Y., et al., *MDR: an integrative DNA N6-methyladenine and N4-methylcytosine modification database for Rosaceae.* Horticulture research, 2019. **6**.
31. Wang, Y., et al., *N6-adenine DNA methylation is associated with the linker DNA of H2A. Z-containing well-positioned nucleosomes in Pol II-transcribed genes in Tetrahymena.* Nucleic acids research, 2017. **45**(20): p. 11594-11606.
32. Jin, J., et al., *iDNA-ABF: multi-scale deep biological language learning model for the interpretable prediction of DNA methylations.* Genome biology, 2022. **23**(1): p. 1-23.
33. Luo, Y., et al., *New developments on the Encyclopedia of DNA Elements (ENCODE) data portal.* Nucleic acids research, 2020. **48**(D1): p. D882-D889.
34. Zhang, J., et al., *An integrative ENCODE resource for cancer genomics.* Nature communications, 2020. **11**(1): p. 3696.
35. Lv, H., et al., *iDNA-MS: an integrated computational tool for detecting DNA modification sites in multiple genomes.* Iscience, 2020. **23**(4).
36. Yu, Y., et al., *iDNA-ABT: advanced deep learning model for detecting DNA methylation with adaptive features and transductive information maximization.* Bioinformatics, 2021. **37**(24): p. 4603-4610.
37. Zeng, W., A. Gautam, and D.H. Huson, *MuLan-Methyl-Multiple Transformer-based Language Models for Accurate DNA Methylation Prediction.* bioRxiv, 2023: p. 2023.01. 04.522704.
38. Zhang, Y., et al., *Multiple sequence alignment-based RNA language model and its application to structural inference.* Nucleic Acids Research, 2024. **52**(1): p. e3-e3.
39. Singh, J., et al., *RNA secondary structure prediction using an ensemble of two-dimensional deep neural networks and transfer learning.* Nature communications, 2019. **10**(1): p. 5407.
40. Chen, J., et al., *Interpretable RNA foundation model from unannotated data for highly accurate RNA structure and function predictions.* bioRxiv, 2022: p. 2022.08. 06.503062.
41. Tan, Z., et al., *TurboFold II: RNA structural alignment and secondary structure prediction informed by multiple homologs.* Nucleic acids research, 2017. **45**(20): p. 11570-11581.
42. Sloma, M.F. and D.H. Mathews, *Exact calculation of loop formation probability identifies folding motifs in RNA secondary structures.* RNA, 2016. **22**(12): p. 1808-1818.

43. Wu, F., et al., *A new coronavirus associated with human respiratory disease in China.* Nature, 2020. **579**(7798): p. 265-269.
44. Sun, L., et al., *Predicting dynamic cellular protein–RNA interactions by deep learning using in vivo RNA structures.* Cell research, 2021. **31**(5): p. 495-516.
45. Sample, P.J., et al., *Human 5′ UTR design and variant effect prediction from a massively parallel translation assay.* Nature biotechnology, 2019. **37**(7): p. 803-809.
46. Akiyama, M. and Y. Sakakibara, *Informative RNA base embedding for RNA structural alignment and clustering by deep representation learning.* NAR genomics and bioinformatics, 2022. **4**(1): p. lqac012.
47. Chen, K., et al., *Self-supervised learning on millions of primary RNA sequences from 72 vertebrates improves sequence-based RNA splicing prediction.* Briefings in Bioinformatics, 2024. **25**(3): p. bbae163.
48. Haeussler, M., et al., *The UCSC genome browser database: 2019 update.* Nucleic acids research, 2019. **47**(D1): p. D853-D858.
49. Zhang, L., et al., *BERT-m7G: a transformer architecture based on BERT and stacking ensemble to identify RNA N7-Methylguanosine sites from sequence information.* Computational and Mathematical Methods in Medicine, 2021. **2021**.
50. Dai, C., et al., *Iterative feature representation algorithm to improve the predictive performance of N7-methylguanosine sites.* Briefings in Bioinformatics, 2021. **22**(4): p. bbaa278.
51. Li, Q., et al., *M6A-BERT-Stacking: A Tissue-Specific Predictor for Identifying RNA N6-Methyladenosine Sites Based on BERT and Stacking Strategy.* Symmetry, 2023. **15**(3): p. 731.
52. Dao, F.-Y., et al., *Computational identification of N6-methyladenosine sites in multiple tissues of mammals.* Computational and structural biotechnology journal, 2020. **18**: p. 1084-1091.
53. Soylu, N.N. and E. Sefer, *BERT2OME: Prediction of 2'-O-methylation Modifications from RNA Sequence by Transformer Architecture Based on BERT.* IEEE/ACM Transactions on Computational Biology and Bioinformatics, 2023.
54. Xuan, J.-J., et al., *RMBase v2. 0: deciphering the map of RNA modifications from epitranscriptome sequencing data.* Nucleic acids research, 2018. **46**(D1): p. D327-D334.
55. Liang, S., et al., *Rm-LR: A long-range-based deep learning model for predicting multiple types of RNA modifications.* Computers in Biology and Medicine, 2023. **164**: p. 107238.
56. Song, Z., et al., *Attention-based multi-label neural networks for integrated prediction and interpretation of twelve widely occurring RNA*

modifications. Nature communications, 2021. **12**(1): p. 4011.
57. Barrett, T., et al., *NCBI GEO: archive for functional genomics data sets—update.* Nucleic acids research, 2012. **41**(D1): p. D991-D995.
58. Ramaswami, G. and J.B. Li, *RADAR: a rigorously annotated database of A-to-I RNA editing.* Nucleic acids research, 2014. **42**(D1): p. D109-D113.
59. Ning, Z., et al., *BertNDA: a Model Based on Graph-Bert and Multi-scale Information Fusion for ncRNA-disease Association Prediction.* bioRxiv, 2023: p. 2023.05. 18.541387.
60. Li, Y., et al., *HMDD v2. 0: a database for experimentally supported human microRNA and disease associations.* Nucleic acids research, 2014. **42**(D1): p. D1070-D1074.
61. Jiang, Q., et al., *miR2Disease: a manually curated database for microRNA deregulation in human disease.* Nucleic acids research, 2009. **37**(suppl_1): p. D98-D104.
62. Bao, Z., et al., *LncRNADisease 2.0: an updated database of long non-coding RNA-associated diseases.* Nucleic acids research, 2019. **47**(D1): p. D1034-D1037.
63. Gao, Y., et al., *Lnc2Cancer 3.0: an updated resource for experimentally supported lncRNA/circRNA cancer associations and web tools based on RNA-seq and scRNA-seq data.* Nucleic acids research, 2021. **49**(D1): p. D1251-D1258.
64. Feng, H., et al., *LncCat: An ORF attention model to identify LncRNA based on ensemble learning strategy and fused sequence information.* Computational and Structural Biotechnology Journal, 2023. **21**: p. 1433-1447.
65. O'Leary, N.A., et al., *Reference sequence (RefSeq) database at NCBI: current status, taxonomic expansion, and functional annotation.* Nucleic acids research, 2016. **44**(D1): p. D733-D745.
66. Xia, S., et al. *A multi-granularity information-enhanced pre-training method for predicting the coding potential of sORFs in plant lncRNAs*. in *2023 IEEE International Conference on Bioinformatics and Biomedicine (BIBM)*. 2023. IEEE.
67. Di Marsico, M., et al., *GreeNC 2.0: a comprehensive database of plant long non-coding RNAs.* Nucleic Acids Research, 2022. **50**(D1): p. D1442-D1447.
68. Babjac, A.N., Z. Lu, and S.J. Emrich. *CodonBERT: Using BERT for Sentiment Analysis to Better Predict Genes with Low Expression*. in *Proceedings of the 14th ACM International Conference on Bioinformatics, Computational Biology, and Health Informatics*. 2023.
69. Nieuwkoop, T., et al., *Revealing determinants of translation efficiency via whole-gene codon randomization and machine learning.* Nucleic

acids research, 2023. **51**(5): p. 2363-2376.
70. Byrska-Bishop, M., et al., *High-coverage whole-genome sequencing of the expanded 1000 Genomes Project cohort including 602 trios.* Cell, 2022. **185**(18): p. 3426-3440. e19.
71. Bernard, C., et al., *RNA-TorsionBERT: leveraging language models for RNA 3D torsion angles prediction.* bioRxiv, 2024: p. 2024.06. 06.597803.
72. Wang, X., et al., *UNI-RNA: universal pre-trained models revolutionize RNA research.* bioRxiv, 2023: p. 2023.07. 11.548588.
73. *RNAcentral: a hub of information for non-coding RNA sequences.* Nucleic Acids Research, 2019. **47**(D1): p. D221-D229.
74. Sayers, E.W., et al., *Database resources of the national center for biotechnology information.* Nucleic acids research, 2022. **50**(D1): p. D20-D26.
75. Chen, M., et al., *Genome Warehouse: a public repository housing genome-scale data.* Genomics, Proteomics and Bioinformatics, 2021. **19**(4): p. 584-589.
76. Chu, Y., et al., *A 5′ UTR language model for decoding untranslated regions of mRNA and function predictions.* Nature Machine Intelligence, 2024. **6**(4): p. 449-460.
77. Yang, Y., et al., *Deciphering 3'UTR Mediated Gene Regulation Using Interpretable Deep Representation Learning.* Advanced Science, 2024. **11**(39): p. 2407013.
78. Wang, N., et al., *Multi-purpose RNA language modelling with motif-aware pretraining and type-guided fine-tuning.* Nature Machine Intelligence, 2024: p. 1-10.

Supplementary Table 2. Detailed information of large language models for proteomic tasks

Application area	Models	Ref	Publication time	Parameters	Architecture	Datasets			Downstream tasks
						Data type	Source	Size	
Protein Large Language Models	MSA Transformer	[1]	Jul 2021	100M parameters model with 12 layers, 768 embedding size, and 12 attention heads	Transformer-based	MSAs	CAMEO [2]	131 domains (129 evaluated)	Unsupervised contact prediction, supervised contact prediction, secondary structure prediction
							CASP13-FM [3]	31 free modeling domains (from 25 targets)	
							trRosetta training set [4]	15,051 MSAs and structures (14,842 used)	
						Protein sequences	CB513 [5]	513 protein sequences	
							Netsurf dataset [6]	12,185 crystal structures obtained from the PDB [7]	
	UniRep	[8]	Dec 2019	18.2M parameters (a 1,900-hidden unit mLSTM with amino-acid character embeddings)	LSTM-based	Protein sequences	UniRef50 [9]	24M	Predicting stability of naturally occurring and de novo designed proteins, Prediction of functional effects of single mutations in diverse proteins
							Mini protein dataset from [10]	1,432 out of 5,570 test set and 1,416 out of 5,571 validation set	
							DMS dataset [11]	~65,420 variants across the 8 proteins	
							avGFP dataset [12]	32,400 variants derived from 27 homologs of avGFP (used)	
	TAPE	[13]	Dec 2019	38M parameters	(ResNet, Transformer, LSTM)-based	Protein sequences	Netsurf dataset [6]	12,185 protein sequences	Secondary structure (SS) prediction (structure prediction task), contact prediction (structure prediction task), remote homology detection (evolutionary understanding task), fluorescence landscape prediction (protein engineering task), stability landscape prediction (protein engineering task)
							ProteinNet dataset [14]	~332,283,871 protein sequences	
							DeepSF dataset [15]	Training set includes 16,712 proteins spanning 1,195 folds [16]; test datasets include 2,533 protein domains across 550 folds from SCOP 2.06 [16], a subset of SCOP	

								1.75 and the CASP dataset [17, 18]	
							avGFP dataset [12]	~51,715 protein sequences	
							Dataset from [10]	~ 46,800 protein sequences	
	ESM-1b	[19]	Dec 2020	A model with ~650M parameters (33 layers)	Transformer-based	Protein sequences	SCOPe [20]	15,297 protein sequences	Remote homology detection, prediction of secondary structure, long-range residue–residue contacts, mutational effect prediction, etc.
							CB513 [5]	513 protein sequences;	
							CASP13 [21]	431 domains	
							Envision (DMS dataset) [11] and DeepSequence [22]	Over 700,000 variant effect measurements from over 100 large-scale experimental mutagenesis datasets	
	ProtTrans	[23]	Aug 2021	From millions to billions parameters (224M-11B)	(Transformer-XL, XLNet, BERT, Albert, Electra, T5)-based	Protein sequences	CB513 [5]	513 protein sequences	Per-residue secondary structure prediction，per-protein localization & membrane prediction
							TS115 [5]	115 protein sequences	
							CASP12 [24]	~102 protein sequences	
							NEW364 [23]	364 protein sequences	
							DeepLoc [25]	~19,817 protein sequences	
							SCOPe 2.07 [26]	14,323 protein sequences (non-redundant at PIDE < 40%)	
	SPRoBERTa	[27]	Sep 2022	12 Transformer encoder layers, the embedding size is 768, the feed-forward hidden units are 3072 and the attention heads are 12	BERT-based	Protein sequences	Netsurf dataset [6]	12,185 protein sequences	Secondary structure prediction, contact prediction, remote homology prediction, protein function prediction or Gene Ontology (GO) term prediction
							CB513 [5]	513 protein sequences	
							CASP12 [24]	~102 protein sequences	
							DeepSF dataset [15]	Consistent with the size used TAPE [13]	

							DeepFRI [28]	~284,832 protein sequences with GO annotations	
	PromptProtein	[29]	Sep 2022	650M parameters with 33 layers and 20 attention heads. The embedding size is 1280	Transformer-based	Protein sequences	EC dataset from [28]	19,199 protein sequences	Enzyme commission and Gene Ontology prediction, stability landscape prediction, fluorescence landscape prediction, thermostability landscape prediction, adeno-associated virus (AAV) landscape prediction, GB1 landscape prediction, antibody-antigen affinity prediction
							GO dataset from [28]	36,641 protein sequences	
							Protein engineering dataset from TAPE and FLIP [13, 30]	68,965 protein sequences used in stability prediction, 54,025 used in fluorescence landscape prediction, 28,131 used in thermostability landscape prediction, 82,583 in AAV prediction, 8,733 in GB1 prediction	
	ProGen	[31]	Mar 2020	1.2B parameters. Sequence length is 512. The model has dimension d = 1028, inner dimension f = 512, 36 layers, and 8 heads per layer. Dropout with probability 0.1 follows the residual connections in each layer	Transformer-based	Protein sequences, Conditioning tags	Uniparc [32], UniprotKB [33], SWISS-PROT [34], TrEMBL [35], Pfam [36], and NCBI taxonomic information [37]	281M	Controllable protein generation and two case study: completing VEGFR2 kinase domain, zero-shot fitness selection for protein GB1
	Tranception	[38]	Jun 2022	700M parameters	Transformer-based	Protein sequences	Protein Gym (a DMS dataset) [38]	~1.8M protein sequences	Fitness prediction
	ProtGPT2	[39]	Jul 2022	738M parameters. The model consists of 36 layers with a model dimensionality of 1280. The architecture matches that of the previously released GPT2-large	GPT-based	Protein sequences	UniRef50 [40]	10,000 protein sequences	Sequence dataset generation, homology detection, disorder prediction
							ProtGPT2 dataset [39]	Generated 10,000 protein sequences	
	ProteinBERT	[41]	Feb 2022	16M parameters. The model architecture consists of two almost parallel paths: one for local representations with	BERT-based	Protein sequences, Gene Ontology (GO) annotations	Secondary structure dataset from	8,678 sequences (train) [13, 42]	(Secondary structure, disorder, Remote homology, fold classes, signal peptide, major PTMs, neuropeptide cleavage,
							Disorder dataset	8,678 sequences (train)	

				d=128 and the other for global representations with d=512			from	[42]	fluorescence, stability) prediction
							Remote homology dataset from	12,312 sequences (train) [13, 43, 44]	
							Fold classes dataset from	15,680 sequences (train) [43, 44]	
							Signal peptide dataset from	16,606 sequences (train) [45]	
							Major PTMs dataset from	43,356 sequences (train) [46]	
							Neuropeptide cleavage dataset from	2,727 sequences (train) [47, 48]	
							Fluorescence dataset from	21,446 sequences (train) [12, 13]	
							Stability dataset from	53,679 sequences (train) [10]	
	ProtST	[49]	Jan 2023	Depends on the parameters of the chosen language model	Multi-models-based (Protein Language Models and Biomedical Language Models)	Protein sequences, property descriptions	ProtDescribe [50, 51]	553,052 aligned pairs of protein sequence and property description	Protein localization prediction, fitness landscape prediction, protein function annotation (totally 11 downstream tasks)
	KeAP	[52]	Jan 2023	Depends on the parameters of the chosen language model	Multi-cascade Bert-like network	Triplet in the format of (Protein, Relation, Attribute)	ProteinKG25 [53]	5M with nearly 600k protein, 50k attribute terms, and 31 relation terms included	Amino acid contact prediction, protein homology detection, protein stability prediction, protein-protein interaction identification, protein-protein binding affinity prediction, and semantic similarity inference
	CaLM	[54]	Jan 2024	86M parameters. 12 transformer layers contain 12 attention heads, with dimension 768. Similar to architectures of ESM family	Transformer-based	Protein-coding DNA (cDNA)	cDNA dataset obtained from the European Nucleotide Archive with a timestamp of April 2022	9,858,385 cDNA sequences of seven model organisms	Melting point prediction, solubility prediction, subcellular localization prediction and function prediction

							Melting temperature dataset [30, 55]	-	
							Subcellular localization dataset [30, 56]	-	
							Solubility dataset [57]	-	
							Gene ontology dataset [58]	-	
							Transcriptomics dataset [59]	-	
							Proteomics dataset [60]	-	
	PLMSearch	[61]	Mar 2024	ESM-1b (650M parameters) [19] and ProtT5-XL-UniRef50 (3B parameters) [23]	Transformer-based	Protein sequences	SCOPe40 [20, 62], New protein search test, Swiss-Prot [34], CATHS40 [63]	~489,764 sequences for training	Homologous protein search
	DHR	[64]	Jul 2024	2 ESM-1b (650M parameters per encoder) [19]	Transformer-based	Protein sequences	UniRef [9], SCOPe [20, 62]	~2 M	Protein homolog detection
Antibody Large Language Models	MHCRoBERTa	[65]	Dec 2021	Model with 12multi-heads and 5 self-attention layers.	ROBERTa-based	Protein sequences	UniProtKB [66]	565,254 protein sequences	Predicting the binding of peptide and major histocompatibility complex (MHC)

							Immune Epitope Database (IEDB) [67]	MHCclass I transmembrane proteins containing HLA-A(1,777 sequences)，HLA-B (2,100 sequences) and HLA-C(1,931 sequences)	
	BERTMHC	[68]	Jun 2021	The model has 12 layers with 12 self-attention heads in each layer	BERT-based	Protein sequences	The data from Kamilla Kjærgaard Jensen [69]	2,413additional MHC–peptide pairs covering 47 MHC class II alleles.	Predicting precisely the binding and presentation of peptides to major histocompatibility complex (MHC) alleles
							Immune Epitope Database (IEDB) [67]	95,638 peptides	
	TCR-BERT	[70]	BioRxiv posted Nov 2021	12 stacked transformer blocks with 8 attention heads, utilizing a hidden representation dimensionality of 768 and featuring 12 transformer layers.	BERT-based	Protein sequences	Pan immune repertoire database (PIRD) [71]	47,040 TRB sequences and 4,607 TRA sequences.	Antigen specificity classification
							VDJdb [72]	58,795 human TCRs and 3,353 mouse TCRs.	
							TCRdb [73]	139,00,913 TRB sequences of unknown antigen binding affinity.	
	Antiformer	[74]	July 2024	he transformer encoder involves 12 stacking layers of transformer with the multi-head self-attention and feed forward network.	BERT-based	Protein sequences Gene expression	The OAS database[75]	55 BCR-seq datasets containing 600 million sequences	Binding specificity prediction
	SC-AIR-BERT	[76]	May 2023	Six standard transformer layers and each layer has four attention heads, the hidden representation dimensionality is 512 and the inter mediate representation dimensionality is 2048	BERT-based	Protein sequences	VDJdb [72]	23,358 unique paired TCRs	Binding specificity prediction
							Immune Epitope Database (IEDB) [67]	18,662 paired TCR sand 589 paired BCRs	
							huARdb [77]	612,077 high-confidence paired full-length α/β chains of	

								TCR sequences	
							CoV-AbDab [78]	1,105,906 paired antibody heavy/light chains	
	AbLang	[79]	Jun 2022	Consists of three modules (Each of AbRep's 12 transformer blocks has 12 attenuated heads, an inner hidden size of 3072 and a hidden size of 768. From AbRep, the rescodings (768 values for each residue) are obtained. AbHead follows the design of RoBERTa's[80] head model, with a hidden size of 768)	BERT-based	Human antibody sequences	Observed Antibody Space (OAS) database [81]	Training sets of 14,126 724 heavy and 187,068 light sequences, and two evaluation sets of 100,000 heavy and 50,000 light sequences	Sequence specific predictions, residue specific predictions, amino acid predictions
							Data from [82]	10,000 naive and 10,000 memory B-cell sequences	
	AntiBERTa		Jul 2022	86M parameters. A 12-layer transformer model. Attention heads is 12, embedding dimension is 768, feedforward layer dimension is 3072	BERT-based	Human antibody sequences	SAbDab [83]	Training/validation/test split of 720/90/90	Trace the B cell origin of the antibody, quantify immunogenicity, predict the antibody's binding site
							BCR repertoire dataset [82]	-	
							TheraSAbDab [84]	191 non-redundant therapeutic antibodies	
	EATLM	[85]	Jan 2023	86M parameters (12 layers, 12 heads, and 768 hidden states)	Transformer-based	Human antibody sequences	Dataset from [86]	Training/validation/test split of 15,128/3,242/3,242	Accurate antigen-binding prediction， paratope prediction， B cell analysis， antibody discovery
							Paratope data from [87]	1,662 CDR segments on 277 antibodies	
							Data from [88]	88,094 sequences with 6 maturation stages	
							A subset of the OAS database [81]	Antibody sequences from 133 SARS-CoV-2 patients and 87 health persons	

References

1. Rao, R.M., et al. *MSA transformer*. in *International Conference on Machine Learning*. 2021.
2. Haas, J., et al., *Continuous Automated Model EvaluatiOn (CAMEO) complementing the critical assessment of structure prediction in CASP12.* Proteins: Structure, Function, and Bioinformatics, 2018. **86**: p. 387-398.
3. Shrestha, R., et al., *Assessing the accuracy of contact predictions in CASP13.* Proteins: Structure, Function, and Bioinformatics, 2019. **87**(12): p. 1058-1068.
4. Yang, J., et al., *Improved protein structure prediction using predicted interresidue orientations.* Proceedings of the National Academy of Sciences, 2020. **117**(3): p. 1496-1503.
5. Cuff, J.A. and G.J. Barton, *Evaluation and improvement of multiple sequence methods for protein secondary structure prediction.* Proteins: Structure, Function, and Bioinformatics, 1999. **34**(4): p. 508-519.
6. Klausen, M.S., et al., *NetSurfP-2.0: Improved prediction of protein structural features by integrated deep learning.* Proteins: Structure, Function, and Bioinformatics, 2019. **87**(6): p. 520-527.
7. Berman, H.M., et al., *The protein data bank.* Nucleic acids research, 2000. **28**(1): p. 235-242.
8. Alley, E.C., et al., *Unified rational protein engineering with sequence-based deep representation learning.* Nature methods, 2019. **16**(12): p. 1315-1322.
9. Suzek, B.E., et al., *UniRef clusters: a comprehensive and scalable alternative for improving sequence similarity searches.* Bioinformatics, 2015. **31**(6): p. 926-932.
10. Rocklin, G.J., et al., *Global analysis of protein folding using massively parallel design, synthesis, and testing.* Science, 2017. **357**(6347): p. 168-175.
11. Gray, V.E., et al., *Quantitative missense variant effect prediction using large-scale mutagenesis data.* Cell systems, 2018. **6**(1): p. 116-124.
12. Sarkisyan, K.S., et al., *Local fitness landscape of the green fluorescent protein.* Nature, 2016. **533**(7603): p. 397-401.
13. Rao, R., et al., *Evaluating protein transfer learning with TAPE.* Advances in neural information processing systems, 2019. **32**.
14. AlQuraishi, M., *ProteinNet: a standardized data set for machine learning of protein structure.* BMC bioinformatics, 2019. **20**: p. 1-10.
15. Hou, J., B. Adhikari, and J. Cheng, *DeepSF: deep convolutional neural network for mapping protein sequences to folds.* Bioinformatics, 2018. **34**(8): p. 1295-1303.

16. Murzin, A.G., et al., *SCOP: a structural classification of proteins database for the investigation of sequences and structures.* Journal of molecular biology, 1995. **247**(4): p. 536-540.
17. Kinch, L.N., et al., *CASP9 target classification.* PROTEINS: structure, function, and bioinformatics, 2011. **79**(S10): p. 21-36.
18. Kinch, L.N., et al., *CASP 11 target classification.* Proteins: Structure, Function, and Bioinformatics, 2016. **84**: p. 20-33.
19. Rives, A., et al., *Biological structure and function emerge from scaling unsupervised learning to 250 million protein sequences.* Proceedings of the National Academy of Sciences, 2021. **118**(15): p. e2016239118.
20. Fox, N.K., S.E. Brenner, and J.-M. Chandonia, *SCOPe: Structural Classification of Proteins—extended, integrating SCOP and ASTRAL data and classification of new structures.* Nucleic acids research, 2014. **42**(D1): p. D304-D309.
21. Moult, J., et al., *Critical assessment of methods of protein structure prediction: Progress and new directions in round XI.* Proteins: Structure, Function, and Bioinformatics, 2016. **84**: p. 4-14.
22. Riesselman, A.J., J.B. Ingraham, and D.S. Marks, *Deep generative models of genetic variation capture the effects of mutations.* Nature methods, 2018. **15**(10): p. 816-822.
23. Elnaggar, A., et al., *ProtTrans: Towards Cracking the Language of Lifes Code Through Self-Supervised Deep Learning and High Performance Computing.* IEEE Transactions on Pattern Analysis and Machine Intelligence, 2021: p. 1-1.
24. Abriata, L.A., et al., *Assessment of hard target modeling in CASP12 reveals an emerging role of alignment-based contact prediction methods.* Proteins: Structure, Function, and Bioinformatics, 2018. **86**: p. 97-112.
25. Almagro Armenteros, J.J., et al., *DeepLoc: prediction of protein subcellular localization using deep learning.* Bioinformatics, 2017. **33**(21): p. 3387-3395.
26. Chandonia, J.-M., N.K. Fox, and S.E. Brenner, *SCOPe: classification of large macromolecular structures in the structural classification of proteins—extended database.* Nucleic acids research, 2019. **47**(D1): p. D475-D481.
27. Wu, L., et al., *SPRoBERTa: protein embedding learning with local fragment modeling.* Briefings in Bioinformatics, 2022. **23**(6): p. bbac401.
28. Gligorijevi, V., et al., *Structure-based protein function prediction using graph convolutional networks.* Nature communications, 2021. **12**(1): p. 3168.
29. Wang, Z., et al. *Multi-level Protein Structure Pre-training via Prompt Learning*. in *The Eleventh International Conference on Learning Representations*. 2022.

30. Dallago, C., et al., *FLIP: Benchmark tasks in fitness landscape inference for proteins.* bioRxiv, 2021: p. 2021-11.
31. Madani, A., et al., *Progen: Language modeling for protein generation.* arXiv preprint arXiv:2004.03497, 2020.
32. Leinonen, R., et al., *UniProt archive.* Bioinformatics, 2004. **20**(17): p. 3236-3237.
33. Bairoch, A., et al., *The universal protein resource (UniProt).* Nucleic acids research, 2005. **33**(suppl_1): p. D154-D159.
34. Bairoch, A., et al., *Swiss-Prot: juggling between evolution and stability.* Briefings in bioinformatics, 2004. **5**(1): p. 39-55.
35. Boeckmann, B., et al., *The SWISS-PROT protein knowledgebase and its supplement TrEMBL in 2003.* Nucleic acids research, 2003. **31**(1): p. 365-370.
36. Bateman, A., et al., *The Pfam protein families database.* Nucleic acids research, 2004. **32**(suppl_1): p. D138-D141.
37. Federhen, S., *The NCBI taxonomy database.* Nucleic acids research, 2012. **40**(D1): p. D136-D143.
38. Notin, P., et al. *Tranception: protein fitness prediction with autoregressive transformers and inference-time retrieval.* in *International Conference on Machine Learning*. 2022.
39. Ferruz, N., S. Schmidt, and B. Hcker, *ProtGPT2 is a deep unsupervised language model for protein design.* Nature communications, 2022. **13**(1): p. 4348.
40. *UniProt: the universal protein knowledgebase in 2021.* Nucleic acids research, 2021. **49**(D1): p. D480-D489.
41. Brandes, N., et al., *ProteinBERT: a universal deep-learning model of protein sequence and function.* Bioinformatics, 2022. **38**(8): p. 2102-2110.
42. Moult, J., et al., *Critical assessment of methods of protein structure prediction (CASP)—Round XII.* Proteins: Structure, Function, and Bioinformatics, 2018. **86**: p. 7-15.
43. Andreeva, A., et al., *SCOP2 prototype: a new approach to protein structure mining.* Nucleic acids research, 2014. **42**(D1): p. D310-D314.
44. Andreeva, A., et al., *The SCOP database in 2020: expanded classification of representative family and superfamily domains of known protein structures.* Nucleic acids research, 2020. **48**(D1): p. D376-D382.
45. Armenteros, J.J.A., et al., *SignalP 5.0 improves signal peptide predictions using deep neural networks.* Nature biotechnology, 2019. **37**: p. 420-423.
46. Hornbeck, P.V., et al., *PhosphoSitePlus, 2014: mutations, PTMs and recalibrations.* Nucleic acids research, 2015. **43**(D1): p. D512-D520.
47. Ofer, D. and M. Linial, *ProFET: Feature engineering captures high-level protein functions.* Bioinformatics, 2015. **31**(21): p. 3429-3436.

48. Brandes, N., D. Ofer, and M. Linial, *ASAP: a machine learning framework for local protein properties.* Database, 2016. **2016**: p. baw133.
49. Xu, M., et al. *Protst: Multi-modality learning of protein sequences and biomedical texts*. in *International Conference on Machine Learning*. 2023. PMLR.
50. Bairoch, A. and R. Apweiler, *The SWISS-PROT protein sequence database and its supplement TrEMBL in 2000.* Nucleic acids research, 2000. **28**(1): p. 45-48.
51. Xu, M., et al., *Protst: Multi-modality learning of protein sequences and biomedical texts.* arXiv preprint arXiv:2301.12040, 2023.
52. Zhou, H.-Y., et al., *Protein Representation Learning via Knowledge Enhanced Primary Structure Modeling.* bioRxiv, 2023: p. 2023-01.
53. Zhang, N., et al., *Ontoprotein: Protein pretraining with gene ontology embedding.* arXiv preprint arXiv:2201.11147, 2022.
54. Outeiral, C. and C.M. Deane, *Codon language embeddings provide strong signals for use in protein engineering.* Nature Machine Intelligence, 2024. **6**(2): p. 170-179.
55. Jarzab, A., et al., *Meltome atlas—thermal proteome stability across the tree of life.* Nature methods, 2020. **17**(5): p. 495-503.
56. Thumuluri, V., et al., *DeepLoc 2.0: multi-label subcellular localization prediction using protein language models.* Nucleic acids research, 2022. **50**(W1): p. W228-W234.
57. Sridharan, S., et al., *Proteome-wide solubility and thermal stability profiling reveals distinct regulatory roles for ATP.* Nature communications, 2019. **10**(1): p. 1155.
58. Unsal, S., et al., *Learning functional properties of proteins with language models.* Nature Machine Intelligence, 2022. **4**(3): p. 227-245.
59. Uhln, M., et al., *Tissue-based map of the human proteome.* Science, 2015. **347**(6220): p. 1260419.
60. Wang, M., et al., *PaxDb, a database of protein abundance averages across all three domains of life.* Molecular \& cellular proteomics, 2012. **11**(8): p. 492-500.
61. Liu, W., et al., *PLMSearch: Protein language model powers accurate and fast sequence search for remote homology.* Nature communications, 2024. **15**(1): p. 2775.
62. Chandonia, J.-M., et al., *SCOPe: improvements to the structural classification of proteins--extended database to facilitate variant interpretation and machine learning.* Nucleic acids research, 2022. **50**(D1): p. D553-D559.
63. Sillitoe, I., et al., *CATH: increased structural coverage of functional space.* Nucleic acids research, 2021. **49**(D1): p. D266-D273.
64. Hong, L., et al., *Fast, sensitive detection of protein homologs using deep dense retrieval.* Nature Biotechnology, 2024: p. 1-13.

65. Wang, F., et al., *MHCRoBERTa: pan-specific peptide-MHC class I binding prediction through transfer learning with label-agnostic protein sequences.* Brief Bioinform, 2022. **23**(3).
66. Boutet, E., et al., *UniProtKB/Swiss-Prot: the manually annotated section of the UniProt KnowledgeBase*, in *Plant bioinformatics: methods and protocols*. 2007, Springer. p. 89-112.
67. Vita, R., et al., *The Immune Epitope Database (IEDB): 2018 update.* Nucleic Acids Res, 2019. **47**(D1): p. D339-D343.
68. Cheng, J., et al., *BERTMHC: improved MHC–peptide class II interaction prediction with transformer and multiple instance learning.* Bioinformatics, 2021. **37**(22): p. 4172-4179.
69. *Improved methods for predicting peptide binding affinity to MHCclass II molecules.* 2017.
70. Wu, K., et al., *TCR-BERT: learning the grammar of T-cell receptors for flexible antigenbinding analyses.* 2021.
71. Zhang, W., et al., *PIRD: Pan Immune Repertoire Database.* Bioinformatics, 2020. **36**(3): p. 897-903.
72. Bagaev, D.V., et al., *VDJdb in 2019: database extension, new analysis infrastructure and a T-cell receptor motif compendium.* Nucleic Acids Research, 2020. **48**(D1): p. D1057-D1062.
73. Chen, S.Y., et al., *TCRdb: a comprehensive database for T-cell receptor sequences with powerful search function.* Nucleic Acids Res, 2021. **49**(D1): p. D468-D474.
74. Wang, Q., et al., *AntiFormer: graph enhanced large language model for binding affinity prediction.* Briefings in Bioinformatics, 2024. **25**(5).
75. Olsen, T.H., F. Boyles, and C.M.J.P.S. Deane, *Observed Antibody Space: A diverse database of cleaned, annotated, and translated unpaired and paired antibody sequences.* 2022. **31**(1): p. 141-146.
76. Zhao, Y., et al., *SC-AIR-BERT: a pre-trained single-cell model for predicting the antigen-binding specificity of the adaptive immune receptor.* Brief Bioinform, 2023. **24**(4).
77. Wu, L., et al., *huARdb: human Antigen Receptor database for interactive clonotype-transcriptome analysis at the single-cell level.* Nucleic Acids Res, 2022. **50**(D1): p. D1244-D1254.
78. Raybould, M.I.J., et al., *CoV-AbDab: the coronavirus antibody database.* Bioinformatics, 2021. **37**(5): p. 734-735.
79. Olsen, T.H., I.H. Moal, and C.M. Deane, *AbLang: an antibody language model for completing antibody sequences.* Bioinformatics Advances, 2022. **2**(1): p. vbac046.

80. Liu, Y., et al., *Roberta: A robustly optimized bert pretraining approach.* arXiv preprint arXiv:1907.11692, 2019.
81. Kovaltsuk, A., et al., *Observed antibody space: a resource for data mining next-generation sequencing of antibody repertoires.* The Journal of Immunology, 2018. **201**(8): p. 2502-2509.
82. Ghraichy, M., et al., *Different B cell subpopulations show distinct patterns in their IgH repertoire metrics.* Elife, 2021. **10**: p. e73111.
83. Dunbar, J., et al., *SAbDab: the structural antibody database.* Nucleic acids research, 2014. **42**(D1): p. D1140-D1146.
84. Marks, C., et al., *Humanization of antibodies using a machine learning approach on large-scale repertoire data.* Bioinformatics, 2021. **37**(22): p. 4041-4047.
85. Wang, D., F. Ye, and H. Zhou, *On pre-trained language models for antibody.* bioRxiv, 2023: p. 2023-01.
86. Mason, D.M., et al., *Optimization of therapeutic antibodies by predicting antigen specificity from antibody sequence via deep learning.* Nature Biomedical Engineering, 2021. **5**(6): p. 600-612.
87. Liberis, E., et al., *Parapred: antibody paratope prediction using convolutional and recurrent neural networks.* Bioinformatics, 2018. **34**(17): p. 2944-2950.
88. Mroczek, E.S., et al., *Differences in the composition of the human antibody repertoire by B cell subsets in the blood.* Frontiers in immunology, 2014. **5**: p. 96.

Supplementary Table 3. Detailed information of large language models for drug-discovery tasks

Application area	Models	Ref	Publication time	Parameters	Architecture	Datasets			Downstream tasks
						Data type	Source	Size	
Predictions of Molecular Properties	SMILES-BERT	[1]	Sep 2019	6 Transformer encoder layers, the feed-forward hidden units are 1024 and the attention heads are 4	BERT-based	SMILES	ZINC [2]	18.69 M used, totally more than 35 M	LogP prediction, PM2 prediction, and molecular property prediction
	ChemBERTa	[3]	Oct 2020	Implementation of RoBERTa uses 12 attention heads and 6 layers, resulting in 72 distinct attention mechanisms.	BERT-based	SMILES	PubChem [4]	Curated a dataset of 77 M unique SMILES from PubChem, divided this dataset into subsets of 100 K, 250 K, 1 M, and 10 M.	Binary classification prediction of barrier permeability properties, binary classification of clinical trial toxicity, whether the compound inhibits HIV replication for binary classification between active and inactive
	ChemBERTa-2	[5]	Sep 2022	Implementation of RoBERTa uses 12 attention heads and 6 layers, resulting in 72 distinct attention mechanisms.	BERT-based	SMILES	PubChem	Over a large corpus of 77 million SMILES strings	brain penetrability, toxicity, solubility, and on-target inhibition
	K-BERT	[6]	Apr 2022	The hidden size of the transformer encoder is 768, and the number of the attention heads is 12. Six transformer encoders were used in KBERT.	BERT-based	SMILES	ChEMBL [7]	1.8 M used	Related tasks on 15 drug-discovery-related datasets including carcinogenicity, respiratory toxicity and drug induced liver injury.

	MOLE-BERT	[8]	Apr 2023	A 5-layer Graph Isomorphism Networks (GINs) whose hidden dimension is 300	BERT-style, graph-based	Molecular graphs	ZINC15 [9]	2 million molecules sampled from the dataset	Related tasks on 8 drug-discovery-related datasets including barrier permeability properties and clinical trial toxicity
Generation of Molecules	MolGPT	[10]	Oct 2021	6 M parameters. Each self-attention layer returns a vector of size 256 that is taken as input by the fully connected network. The hidden layer of the neural network outputs a vector of size 1024 and passes it through GELU activation layer. The final layer of the fully connected neural network returns a vector of size 256, that is then used as input for the next decoder block. MolGPT consists of eight such decoder blocks	GPT-based	SMILES	MOSES [11] and GuacaMol [12]	1.9 M, 1.6 M	Generating molecules
Drug-Target Interaction	DTI-BERT	[13]	Jun 2022	The proteins can be represented via 1024-D vectors (dimensionality of the features extracted by the ProtBert model). Drug molecular fingerprints are represented by 128-D vectors through semi decomposition process discrete wavelet transform (DWT). Secondly, the 1152-D vectors (a concatenation of protein sequence feature and drug feature) are fed into the feature extraction model to generate interaction information	BERT-based	Molecular fingerprints and protein sequence pairs	DrugBanks [14], BRENDA, SuperTarget, and KEGG BRITE [15]	4,803 drug-target pairs in positive subsets 9,606 synthesized negative pairs	A pair belongs to an interactive drug-target pair or non-interactive drug-target pair
	TransDTI	[16]	Jan 2022	Consist of SMILES-BERT and fine-tuned large protein models.	BERT-based	Molecular SMILES and protein sequence pairs	KIBA [17] , gold-standard external data sets from DTI-MLCD [18]	30, 474 compounds, 961 targets and 61, 624 interactions	A three-classification based on binding affinity

	C2P2	[19]	Jul 2022	Consist of ChemBERTa and ESM model	BERT-based	Molecular SMILES and protein sequence pairs	STRING [20], STITCH [21], Davis [22], and PDBBind v2019 [23, 24]	Over 67.6 million proteins with over 20 billion protein–protein pairs, over 0.5 million chemicals with over 1.6 billion interactions, 30,056 interactions, 14,011 interactions	Binding affinity prediction
	Hyeunseok Kang et al.	[25]	Aug 2022	Consist of ChemBERTa and ProtBERT model	BERT-based	Molecular SMILES and protein sequence pairs	BIOSNAP [26], DAVIS [22] and BindingDB [27]	27,482 interactions, 11,103 interactions, 32,601 interactions	Binding affinity prediction

References

1. Wang, S., et al. *Smiles-bert: large scale unsupervised pre-training for molecular property prediction*. in *Proceedings of the 10th ACM international conference on bioinformatics, computational biology and health informatics*. 2019.
2. Irwin, J.J., et al., *ZINC: a free tool to discover chemistry for biology.* Journal of chemical information and modeling, 2012. **52**(7): p. 1757-1768.
3. Chithrananda, S., G. Grand, and B. Ramsundar, *ChemBERTa: large-scale self-supervised pretraining for molecular property prediction.* arXiv preprint arXiv:2010.09885, 2020.
4. Kim, S., et al., *PubChem 2019 update: improved access to chemical data.* Nucleic acids research, 2019. **47**(D1): p. D1102-D1109.
5. *ChemBERTa-2: Towards Chemical Foundation Models.*
6. Wu, Z., et al., *Knowledge-based BERT: a method to extract molecular features like computational chemists.* Briefings in Bioinformatics, 2022. **23**(3): p. bbac131.
7. Mendez, D., et al., *ChEMBL: towards direct deposition of bioassay data.* Nucleic acids research, 2019. **47**(D1): p. D930-D940.
8. Xia, J., et al. *Mole-bert: Rethinking pre-training graph neural networks for molecules*. in *The Eleventh International Conference on Learning Representations*. 2022.
9. Sterling, T. and J.J. Irwin, *ZINC 15--ligand discovery for everyone.* Journal of chemical information and modeling, 2015. **55**(11): p. 2324-2337.
10. Bagal, V., et al., *MolGPT: molecular generation using a transformer-decoder model.* Journal of Chemical Information and Modeling, 2021. **62**(9): p. 2064-2076.
11. Polykovskiy, D., et al., *Molecular sets (MOSES): a benchmarking platform for molecular generation models.* Frontiers in pharmacology, 2020. **11**: p. 565644.
12. Brown, N., et al., *GuacaMol: benchmarking models for de novo molecular design.* Journal of chemical information and modeling, 2019. **59**(3): p. 1096-1108.
13. Zheng, J., X. Xiao, and W.-R. Qiu, *DTI-BERT: identifying drug-target interactions in cellular networking based on BERT and deep learning method.* Frontiers in Genetics, 2022. **13**: p. 859188.
14. Wishart, D.S., et al., *DrugBank 5.0: a major update to the DrugBank database for 2018.* Nucleic acids research, 2018. **46**(D1): p. D1074-D1082.
15. Hu, J., et al., *GPCR--drug interactions prediction using random forest with drug-association-matrix-based post-processing procedure.* Computational biology and chemistry, 2016. **60**: p. 59-71.
16. Kalakoti, Y., S. Yadav, and D. Sundar, *TransDTI: transformer-based language models for estimating DTIs and building a drug recommendation workflow.* ACS omega, 2022. **7**(3): p. 2706-2717.
17. Tang, J., et al., *Making sense of large-scale kinase inhibitor bioactivity data sets: a comparative and integrative analysis.* Journal of Chemical Information and Modeling, 2014. **54**(3): p. 735-743.
18. Chu, Y., et al., *DTI-MLCD: predicting drug-target interactions using multi-label learning with community detection method.* Briefings in bioinformatics, 2021. **22**(3):

p. bbaa205.
19. Nguyen, T.M., T. Nguyen, and T. Tran, *Mitigating cold-start problems in drug-target affinity prediction with interaction knowledge transferring.* Briefings in Bioinformatics, 2022. **23**(4): p. bbac269.
20. Szklarczyk, D., et al., *The STRING database in 2021: customizable protein--protein networks, and functional characterization of user-uploaded gene/measurement sets.* Nucleic acids research, 2021. **49**(D1): p. D605-D612.
21. Kuhn, M., et al., *STITCH: interaction networks of chemicals and proteins.* Nucleic acids research, 2007. **36**(suppl_1): p. D684-D688.
22. Davis, M.I., et al., *Comprehensive analysis of kinase inhibitor selectivity.* Nature Biotechnology, 2011. **29**(11): p. 1046-1051.
23. Wang, R., et al., *The PDBbind database: Collection of binding affinities for protein- ligand complexes with known three-dimensional structures.* Journal of medicinal chemistry, 2004. **47**(12): p. 2977-2980.
24. Wang, R., et al., *The PDBbind database: methodologies and updates.* Journal of medicinal chemistry, 2005. **48**(12): p. 4111-4119.
25. Kang, H., et al., *Fine-tuning of bert model to accurately predict drug--target interactions.* Pharmaceutics, 2022. **14**(8): p. 1710.
26. Zitnik, M., R. Sosic, and J. Leskovec, *BioSNAP Datasets: Stanford biomedical network dataset collection.* Note: http://snap. stanford. edu/biodata Cited by, 2018. **5**(1).
27. Liu, T., et al., *BindingDB: a web-accessible database of experimentally determined protein--ligand binding affinities.* Nucleic acids research, 2007. **35**(suppl_1): p. D198-D201.

Supplementary Table 4. Detailed information of large language models for single-cell tasks

Application area	Models	Ref	Publication time	Parameters	Architecture	Fine-tuning datasets			Downstream tasks
						Data type	Source	Size	
Single-cell large language models	scBERT	[1]	Sep 2022	six Performer encoder layers and ten heads for each layer, 200 dimensions of gene embedding using gene2vec	BERT-based	scRNA-seq	The Panglao dataset [2]	209 human single-cell datasets comprising 74 tissues with 1,126,580 cells	Cell type annotation, novel cell type discovery, robustness to batch effects and model interpretability
							Zheng68k dataset [3]	68,450 cells	
							Pancreas datasets [4-7]	-	
							MacParland dataset [8]	8,444 cells	
							Heart datasets [9, 10]	451,513 cells for pretraining and the 287,269 cells for benchmarking	
							Lung dataset [11]	39,778 cells	
							Human Cell Atlas dataset [12]	84,363 cells from 27 cell types among 15 major organs	
	scGPT	[13]	Feb 2024	12 stacked transformer blocks with 8 attention heads, 512 embedding sizes of the pre-trianed foundation model, 512 hidden sizes of the fully connected layer	GPT-based	scRNA-seq	CELLxGENE scRNA-seq human PBMC Collection [14]	33 million human PBMC scRNA-seq samples	Gene network inference
							PBMC 10K dataset [15]	Two scRNA-seq data of 7,982 cells and 4,008 cells.	Multi-batch integration
							Immune Human dataset [16]	33,506 cells	
							hPancreas dataset [4-7, 17, 18]	10,600 cells in the reference set and 4,218 cells in the query set	Cell type annotation
							Adamson dataset [19]	87 unique one-gene perturbations, each replicated in around 100 cells	Genetic perturbation prediction
							Norman dataset [20]	131 two-gene perturbations and 105 one-gene perturbations. Each perturbation is replicated in around 300-700 cells.	
						Single-cell multi-omics	10X Multiome PBMC [21]	9,631 cells	Multi-omic integration
							ASAP PBMC [22]	Four datasets each contain 5,023, 3,666, 3,517, and 4,849 cells respectively	
	CIForm	[23]	July 2023	64 attention heads	BERT-based	scRNA-seq	Pancreas datasets [4-7]	-	Cell type annotation
							Immune datasets [24-26]	-	
							Brain datasets [27-29]	-	
							Tabula Muris dataset [30]	Nearly 100000 cells from 20 organs and tissues	

							Zheng68k dataset [3]	68,450 cells	
							ZhangTdataset [31]	8,530 cells from 20 subtypes	
							Allen mouse brain dataset [32]	12832cells	
	TOSICA	[17]	Jan 2023	-	BERT-based	scRNA-seq	human pancreas (hPancreas) [4-7]	10,600 cells for training and 4,218 for testing	Cell type annotation, new cell type discovery and batch correction
							human bone (hBone, GSE152805) [33]	14,615 cells for training and 11,525 for testing	
							human artery (hArtery, GSE159677) [34]	10,960 cells for training and 35,399 for testing	
							mouse brain (mBrain) [28-30, 35]	48,801 cells for training and 7,394 for testing	
							mouse pancreas (mPancreas, GSE132188) [36]	25,465 cells for training and 10,886 for testing	
							mouse atlas (mAtlas, GSE132042) [37]	78,672 cells for training and 277,541 for testing	
	scTransSort	[38]	Mar 2023	12 layers of transformer	BERT-based	scRNA-seq	human cell atlas dataset	295,805 cells from 35 tissues	Cell type annotation
							mouse cell atlas dataset	105,148 cells from 26 tissues and 103,148 cells from 26 tissues	
							data processed by Shao X et al. [39]	-	
	TransCluster	[40]	Oct 2022	5 attention heads	improved Transformer model	scRNA-seq	Human training data [41]	-	Cell type Identification
							The Shao dataset [39]	-	
							The Baron dataset [4]	-	
	Geneformer	[42]	May 2023	six transformer encoder units, input size of 2,048, 256 embedding dimensions, four attention heads per layer and feed forward size of 512	BERT-based	scRNA-seq	iPSC differentiation data	Assayed in parallel on the Drop-seq (single cell) or DroNc-seq (single nucleus) platform	Batch effect removal, cell type annotation
							Huggingface Dataset [43]	a largescale pretraining corpus, Genecorpus-30M, comprising 29.9 million human single-cell transcriptomes	discover key network regulators and candidate therapeutic targets
	tGPT	[44]	May 2023	8 transformer decoder blocks with 1024 hidden units and 16 attention heads	GPT-based	scRNA-seq	Human Cell Atlas Census of Immune Cells (HCA) [45]	282,588 Bone marrow cells from 64 healthy donors in Human Cell Atlas (HCA) project	single-cell clustering, inference of developmental lineage
							Human cell Landscape (HCL) [41]	586,135 human cells	
							Tabula Mursi dataset [30]	54,865 cells	
							Macaque Retina dataset [46]	124,965 cells	

						Bulk RNA-seq	The Cancer Genome Atlas (TCGA) [47]	9,318 bulk samples	interrogation of feature representation of bulk tissues in relation to genomic alterations, prognosis and treatment response of immunotherapy
							Genotype-Tissue Expression Project (GTEx) [48]	11,688 bulk samples	
	DeepMAPS	[49]	Feb 2023	-	BERT-based, graph transformer	scRNA-seq	Multiple scRNA-seq data [4, 16]	Three scRNA-seq datasets with 20,125 cells, 14,878 cells and 16,382 cells	Cell clustering, infer cell-type-specific biological networks from scMulti-omics data
						Single-cell multi-omics	CITE-seq data [50]	Three CITE-seq datasets with 25,171 cells, 32,029 cells and 16,750 cells	
							scRNA-ATAC-seq data	Four scRNA-ATAC-seq datasets with 3,009 cells, 11,898 cells, 3,233 cells and 10,970 cells	
	scMVP	[51]	Jan 2022	8 self-attention heads and each head takes 16-dimension feature	Transformer-based	Paired scRNA-seq and scATAC-seq data	sci-CAR cell line dataset [52]	293T cell line, 3T3 cell line, 293T/3T3 cell mixture, and A549 cell line treated with dexamethasone (DEX) for 0 h, 1 h, and 3 h	dimensionality reduction, cell clustering, and developmental trajectory inference and generate separate imputations for differential analysis and cis-regulatory element identification
							Paired-seq cell line dataset [53]	derived from HEK293, HepG2, and their cell line mixture	
							SNARE-seq cell line dataset [54]	5,081 cells	
							SHARE-seq [55]	67,418 cells	
	scTranslator	[56]	BioRxiv posted July 2023	8-headed attention mechanism in each sub-layer, 117 million parameters.	Transformer-based	Bulk datasets	The Cancer Genome Atlas (TCGA) data [57-60]	31 cancer types and 18,227 samples in total	translate single-cell transcriptome to proteome, predict protein abundance
							The data from Clinical Proteomic Tumor Analysis Consortium (CPTAC) [61-67]		
							The dataset from Broad Institute [68, 69]		
							The dataset from Memorial Sloan Kettering Cancer Center (MSKCC) [70]		
						Single-cell	The Seurat v4 PBMCs dataset	161,764 human peripheral blood	

						datasets	[71]	mononuclear cells	
							The REAP-seq PBMCs dataset [72]	4,330 PBMCs with simultaneous measurements of 44 proteins and 21,005 transcriptome genes	
							The CITE-seq CBMCs dataset [73]	simultaneous measurements of 13 cellular proteins and 16,508 transcriptome genes. This dataset includes 8,005 cord blood mononuclear cells (CBMCs)	
							The single-cell pan-cancer dataset [74]	65,698 myeloid cells with only single-cell transcriptome data, involving 15,844 genes	
	scFoundation	[75]	June 2024	100 million parameters	BERT-based	Single-cell datasets	Baron dataset [4]	-	gene expression enhancement, tissue drug response prediction, cell clustering, single-cell drug response classification, and single-cell perturbation prediction
							Zheng68K dataset [3]	68,450 cells	
							Cancer drug response dataset [76]	-	
							Single cell drug response classification dataset	-	
							Perturbation dataset [77]	-	
	scMoFormer	[78]	Oct 2023	Graph transformer	Transformer-based	Single-cell multi-omics	Joint measurements of gene expression and surface protein levels datasets from the NeurIPS multimodal single-cell integration competition of the year 2021 [25] and 2022	-	use gene expression (RNA) to predict surface protein level, protein levels to gene expression, gene expression to chromatin accessibility and chromatin accessibility to gene expression
	GeneCompass	[79]	Sep 2024	12-layer transformer framework, 100 million parameters	BERT-based	single-cell transcriptomes	CHIP-Atlas related to PBMC cells on GSE43036 [80]	-	Gene embedding analysis, Gene expression profiling prediction
							human multiple sclerosis	-	cell type annotation

							(hMS), lung (hLung) and liver (hLiver) datasets, and mouse brain (mBrain), lung (mLung) and pancreas (mPancreas) datasets.		
							Immune Human [16]	33,506 cells	GRN inference
							dataset provided by Srivatsan et al. [81]	-	Drug dose response prediction
							predefined dosage-sensitive and nonsensitive gene datasets [42]	-	Gene dosage sensitivity predictions.
							Norman dataset [20]	131 two-gene perturbations and 105 one-gene perturbations. Each perturbation is replicated in around 300-700 cells.	In silico perturbation
							mouse embryonic stem cells (ESCs) [82]	-	In silico quantitative perturbation
	scMulan	[83]	BioRxiv posted Jan 2024	24-layer transformer, 368 million parameters	GPT-based	single-cell transcriptomic data	AHCA_BoneMarrow [12]	3,000 bone marrow cells from a single adult donor	zero-shot cell type annotation
							Simonson2023 [84]	60,345 cells from 8 human left ventricle samples	
							Suo2022 [85]	140,000 liver cells from 14 fetal donors at various developmental stages	
							Intestine_HCL_55k dataset [41]	55,214 intestinal cells across 24 cell types	
							Immune cell dataset [86]	274,346 cells spanning 18 batches	batch integration
							Lung dataset [16]	32,472 cells from 16 donors	
							six organs within the hECA-10M dataset [83]	3,000 conditions	conditional cell generation
	UCE	[87]	BioRxiv posted Nov 2023	a 33-layer model consisting of over 650 million parameters	BERT-based	single-cell gene expression dataset	Tabula Sapiens v2 dataset	human data from 581,430 cells, 27 tissues, batches and 162 unique cell types	zero-shot embedding of new datasets
							a dataset of green monkey lymph node and lung cells [88]	17 cell types	Cell type embedding for new species
							naked mole rat spleen and circulating immune cells [89]	24 cell types	
							two distinct chicken datasets (chick retina [90] and	15 cell types in chicken heart dataset	

							developing chick heart [91])		
							mouse renal cells [92]		decode the function of newly discovered cell types
	CellLM	[93]	arXiv posted June 2023	Performer model, 10 layers, 16 attention heads, over 50 million parameters	BERT-based	scRNA-seq data	Zheng68k dataset [3]	68,450 human peripheral blood mononuclear cells (PBMCs) with 11 highly related cell types	Cell type annotation
							The pancreas Baron dataset [4]	8,562 cells categorized into 13 different cell types	
							Human lung cancer cells (GSE149383) [94]	2,739 cells	Single-cell drug sensitivity prediction
							human oral squamous cancer cells (GSE117872) [95-97]	1,302 cells	
						Single-omics cell line data	cell lines integrating from CCLE [98] and GDSC [99]	555 cell lines and 223 drugs	Single-omics cell line drug sensitivity prediction
	scCLIP	[100]	Oct 2023	Vanilla transformers	transformer-based encoders	Paired scRNA and scATAC-seq data	Fetal Atlas [101, 102]	377, 134 cells	integration of multi-modal single-cell sequencing data
							Brain [103]	-	
	iSEEEK	[104]	Jan 2022	8 transformer layers each with 576 hidden units and 8 attention heads	BERT-based	single-cell expression data	PBMCs [105]	43,073 cells	Cell clustering
							Human Cell Atlas Census of Immune Cells (HCA) [45]	282,588 Bone marrow cells from 64 healthy donors in Human Cell Atlas (HCA) project	
							Tabula Muris dataset [30]	Nearly 100000 cells from 20 organs and tissues	
							Zheng68k dataset [3]	68,579 cells	
							the dataset of FACS-sorted CD4/8+ T cells [31, 106, 107]	12,670 CD4+ and 9,012 CD8+ T cells	Identify gene–gene interaction networks
	CellPLM	[108]	BioRxiv posted Oct 2023	over 80 million parameters	BERT-based	scRNA-seq and spatially-resolved transcriptomic (SRT) data	dataset from Li et al. [109]	48, 082 cells	zero-shot clustering
							PBMC 5K and Jurkat from 10x Genomics	33,538 cells in PBMC 5K and 32,738 cells in Jurkat	scRNA-seq denoising
							two spatial transcriptomic datasets at single-cell resolution, i.e., Lung2 and Liver2	836,739 cells in Lung2 and 598,141 cells in Liver2	spatial transcriptomic imputation
							hPancreas [17] and Multiple Sclerosis (MS) [110]	-	cell type annotation
							the Adamson Perturb-Seq	87 one-gene perturbations in the	perturbation

							dataset [19] and the Norman Perturb-Seq dataset [20]	Adamson Perturb-Seq dataset, and 131 two-gene perturbations and 105 one-gene perturbations in the Norman Perturb-Seq dataset	prediction
	scGREAT	[111]	Feb 2024	-	Transformer-based	single-cell transcriptomics	human embryonic stem cells (hESC) (GSE75748) [112]	-	gene regulatory network inference
							human mature hepatocytes (hHEP) (GSE81252) [113, 114]	-	
							mouse dendritic cells (mDC) (GSE48968) [115]	-	
							mouse embryonic stem cells (mESC) (GSE98664) [116]	-	
							mouse hematopoietic stem cells with erythroid-lineage (mHSC-E) mouse hematopoietic stem cells with granulocyte-monocyte-lineage (mHSC-GM) mouse hematopoietic stem cells with lymphoid-lineage (mHSC-L) (GSE81682) [117]	-	
	BioFormers	[118]	bioRxiv posted Dec 2023	An 8-layer transformer encoder model with 8 self-attention heads per layer and a hidden state dimension of 512	BERT-based	scRNA-seq	PBMC [15]	7,982 cells and 3,346 genes	Cell clustering and identification
							PBMC 4k and 8k [119]	11,990 cells and 2,000 HVGs	Gene expression prediction
							Perturb-seq dataset [19]	87 single-gene perturbations, with ~100 cells per perturbation and a control set of at least 7,000 unperturbed cells	genetic perturbation prediction and gene network inference
	scPRINT	[120]	bioRxiv posted July 2024	2M to 100M parameters, 4-layer transformer encoder model with 2 self-attention heads per layer	BERT-based	scRNA-seq	three test datasets of kidney, retina, and colon tissues [121-123]	comprising 26 cell types	gene network inference, denoising, batch effect correction, and cell label prediction
							perturb-seq [124] and ChIP-seq [125]	-	
							3 test datasets of ciliary body, colon, and retina [122, 126, 127]	-	
							premalignant neoplasms from	-	

							human prostate tissues [128]		
	ScRAT	[129]	Feb 2024	one-layer transformer encoder model with 8 self-attention heads	Transformer-based	scRNA-seq	COMBAT [130] and Haniffa datasets [131]	835,937 and 528,438 cells	disease diagnosis
							SC4 with COVID samples [132]	501,943 cells for severity prediction<br>1,289,496 cells for stage prediction	predict severity and stage
	CancerFoundation	[133]	bioRxiv posted Nov 2024	Six transformer layers, 10.8 million parameters	BERT-based	scRNA-seq	glioblastoma dataset [134]	four distinct malignant cell states	batch integration
							CCLE [98] and GDSC [99]	-	Drug response prediction
						bulk RNA-seq data	TCGA data [135, 136]	21 cancer types	Survival prediction
	mcBERT	[137]	bioRxiv posted Nov 2024	12 blocks, each with 12 attention heads	BERT-based	scRNA-seq	Heart [9, 84, 138-141]	Refer to Table 1 in [137]	patient-level representation, disease clustering, phenotypical interpretation/batch effect removal
							Kidney [142-146]		
							PBMC [130, 147, 148]		
							Lung [149]		
	Nicheformer	[150]	bioRxiv posted Oct 2024	The transformer block leverages 12 transformer encoder units19,27 with 16 attention heads per layer and a feed-forward network size of 1,024 to generate a 512-dimensional embedding of the pretraining dataset, resulting in altogether 49.3M parameters.	BERT-based	single-cell and spatial transcriptomics data	MERFISH mouse brain [151]	4.3 million cells across 59 tissue sections	Label prediction (cell type, niche and region labels)
							CosMx human liver [152]	332,877 healthy cells and 460,441 cancer cells	niche label prediction (healthy data only), niche composition prediction
							CosMx human lung [152]	five different donors (301,611, 89,975, 227,110, 71,304 and 81,236 cells, respectively)	niche composition prediction
							Xenium human lung from 10X Genomics	295,883 healthy cells and 531,165 cancer cells	neighborhood density prediction
							Xenium human colon from 10X Genomics	275,822 healthy cells and 587,115 cancer cells	
	SpaFormer	[153]	arXiv posted Feb 2023	2 layers and 8 heads	Transformer-based	Spatial transcriptomics data	Lung 5 data generated by the CosMX platform [152]	99,656 cells	spatial transcriptomic imputation
							Kidney 1139 data generated by the CosMX platform [152]	61,283 cells	
							Liver normal generated by the CosMX platform [152]	305,730 cells	

References

1. Yang, F., et al., *scBERT as a large-scale pretrained deep language model for cell type annotation of single-cell RNA-seq data.* Nature Machine Intelligence, 2022. **4**(10): p. 852-866.
2. Franzen, O., L.M. Gan, and J.L.M. Bjorkegren, *PanglaoDB: a web server for exploration of mouse and human single-cell RNA sequencing data.* Database (Oxford), 2019. **2019**.
3. Zheng, G.X., et al., *Massively parallel digital transcriptional profiling of single cells.* Nat Commun, 2017. **8**: p. 14049.
4. Baron, M., et al., *A Single-Cell Transcriptomic Map of the Human and Mouse Pancreas Reveals Inter- and Intra-cell Population Structure.* Cell Syst, 2016. **3**(4): p. 346-360 e4.
5. Muraro, M.J., et al., *A Single-Cell Transcriptome Atlas of the Human Pancreas.* Cell Syst, 2016. **3**(4): p. 385-394 e3.
6. Segerstolpe, A., et al., *Single-Cell Transcriptome Profiling of Human Pancreatic Islets in Health and Type 2 Diabetes.* Cell Metab, 2016. **24**(4): p. 593-607.
7. Xin, Y., et al., *RNA Sequencing of Single Human Islet Cells Reveals Type 2 Diabetes Genes.* Cell Metab, 2016. **24**(4): p. 608-615.
8. MacParland, S.A., et al., *Single cell RNA sequencing of human liver reveals distinct intrahepatic macrophage populations.* Nat Commun, 2018. **9**(1): p. 4383.
9. Litvinukova, M., et al., *Cells of the adult human heart.* Nature, 2020. **588**(7838): p. 466-472.
10. Tucker, N.R., et al., *Transcriptional and Cellular Diversity of the Human Heart.* Circulation, 2020. **142**(5): p. 466-482.
11. Lukassen, S., et al., *SARS-CoV-2 receptor ACE2 and TMPRSS2 are primarily expressed in bronchial transient secretory cells.* EMBO J, 2020. **39**(10): p. e105114.
12. He, S., et al., *Single-cell transcriptome profiling of an adult human cell atlas of 15 major organs.* Genome Biol, 2020. **21**(1): p. 294.
13. Cui, H., et al., *scGPT: toward building a foundation model for single-cell multi-omics using generative AI.* Nat Methods, 2024. **21**(8): p. 1470-1480.
14. (n.d.)., C.Z.I., *CZ CELLxGENE Discover*. 2022, https://cellxgene.cziscience.com/.
15. Gayoso, A., et al., *A Python library for probabilistic analysis of single-cell omics data.* Nat Biotechnol, 2022. **40**(2): p. 163-166.
16. Luecken, M.D., et al., *Benchmarking atlas-level data integration in single-cell genomics.* Nat Methods, 2022. **19**(1): p. 41-50.
17. Chen, J., et al., *Transformer for one stop interpretable cell type annotation.* Nat Commun, 2023. **14**(1): p. 223.

18. Lawlor, N., et al., *Single-cell transcriptomes identify human islet cell signatures and reveal cell-type-specific expression changes in type 2 diabetes.* Genome Res, 2017. **27**(2): p. 208-222.
19. Adamson, B., et al., *A Multiplexed Single-Cell CRISPR Screening Platform Enables Systematic Dissection of the Unfolded Protein Response.* Cell, 2016. **167**(7): p. 1867-1882 e21.
20. Norman, T.M., et al., *Exploring genetic interaction manifolds constructed from rich single-cell phenotypes.* Science, 2019. **365**(6455): p. 786-793.
21. Cusanovich, D.A., et al., *Multiplex single cell profiling of chromatin accessibility by combinatorial cellular indexing.* Science, 2015. **348**(6237): p. 910-4.
22. Mimitou, E.P., et al., *Scalable, multimodal profiling of chromatin accessibility, gene expression and protein levels in single cells.* Nat Biotechnol, 2021. **39**(10): p. 1246-1258.
23. Xu, J., et al., *CIForm as a Transformer-based model for cell-type annotation of large-scale single-cell RNA-seq data.* Brief Bioinform, 2023. **24**(4).
24. Sun, Z., et al., *A Bayesian mixture model for clustering droplet-based single-cell transcriptomic data from population studies.* Nat Commun, 2019. **10**(1): p. 1649.
25. Oetjen, K.A., et al., *Human bone marrow assessment by single-cell RNA sequencing, mass cytometry, and flow cytometry.* JCI Insight, 2018. **3**(23).
26. Dahlin, J.S., et al., *A single-cell hematopoietic landscape resolves 8 lineage trajectories and defects in Kit mutant mice.* Blood, 2018. **131**(21): p. e1-e11.
27. Zeisel, A., et al., *Molecular Architecture of the Mouse Nervous System.* Cell, 2018. **174**(4): p. 999-1014 e22.
28. Saunders, A., et al., *Molecular Diversity and Specializations among the Cells of the Adult Mouse Brain.* Cell, 2018. **174**(4): p. 1015-1030 e16.
29. Rosenberg, A.B., et al., *Single-cell profiling of the developing mouse brain and spinal cord with split-pool barcoding.* Science, 2018. **360**(6385): p. 176-182.
30. Tabula Muris, C., et al., *Single-cell transcriptomics of 20 mouse organs creates a Tabula Muris.* Nature, 2018. **562**(7727): p. 367-372.
31. Zhang, L., et al., *Lineage tracking reveals dynamic relationships of T cells in colorectal cancer.* Nature, 2018. **564**(7735): p. 268-272.

32. Tasic, B., et al., *Shared and distinct transcriptomic cell types across neocortical areas.* Nature, 2018. **563**(7729): p. 72-78.
33. Chou, C.H., et al., *Synovial cell cross-talk with cartilage plays a major role in the pathogenesis of osteoarthritis.* Sci Rep, 2020. **10**(1): p. 10868.
34. Alsaigh, T., et al., *Decoding the transcriptome of calcified atherosclerotic plaque at single-cell resolution.* Commun Biol, 2022. **5**(1): p. 1084.
35. Zeisel, A., et al., *Brain structure. Cell types in the mouse cortex and hippocampus revealed by single-cell RNA-seq.* Science, 2015. **347**(6226): p. 1138-42.
36. Bastidas-Ponce, A., et al., *Comprehensive single cell mRNA profiling reveals a detailed roadmap for pancreatic endocrinogenesis.* Development, 2019. **146**(12).
37. Tabula Muris, C., *A single-cell transcriptomic atlas characterizes ageing tissues in the mouse.* Nature, 2020. **583**(7817): p. 590-595.
38. Jiao, L., et al., *scTransSort: Transformers for Intelligent Annotation of Cell Types by Gene Embeddings.* Biomolecules, 2023. **13**(4).
39. Shao, X., et al., *scDeepSort: a pre-trained cell-type annotation method for single-cell transcriptomics using deep learning with a weighted graph neural network.* Nucleic Acids Res, 2021. **49**(21): p. e122.
40. Song, T., et al., *TransCluster: A Cell-Type Identification Method for single-cell RNA-Seq data using deep learning based on transformer.* Front Genet, 2022. **13**: p. 1038919.
41. Han, X., et al., *Construction of a human cell landscape at single-cell level.* Nature, 2020. **581**(7808): p. 303-309.
42. Theodoris, C.V., et al., *Transfer learning enables predictions in network biology.* Nature, 2023. **618**(7965): p. 616-624.
43. Lhoest, Q., et al., *Datasets: A community library for natural language processing.* arXiv preprint arXiv:2109.02846, 2021.
44. Shen, H., et al., *Generative pretraining from large-scale transcriptomes for single-cell deciphering.* iScience, 2023. **26**(5): p. 106536.
45. Regev, A., et al., *The human cell atlas white paper.* arXiv preprint arXiv:1810.05192, 2018.
46. Peng, Y.R., et al., *Molecular Classification and Comparative Taxonomics of Foveal and Peripheral Cells in Primate Retina.* Cell, 2019. **176**(5): p. 1222-1237 e22.
47. Johnson, W.E., C. Li, and A. Rabinovic, *Adjusting batch effects in microarray expression data using empirical Bayes methods.* Biostatistics, 2007. **8**(1): p. 118-27.
48. Huang, T.X. and L. Fu, *The immune landscape of esophageal cancer.* Cancer Commun (Lond), 2019. **39**(1): p. 79.

49. Ma, A., et al., *Single-cell biological network inference using a heterogeneous graph transformer.* Nat Commun, 2023. **14**(1): p. 964.
50. Luecken, M.D., et al. *A sandbox for prediction and integration of DNA, RNA, and proteins in single cells*. in *Thirty-fifth conference on neural information processing systems datasets and benchmarks track (Round 2)*. 2021.
51. Li, G., et al., *A deep generative model for multi-view profiling of single-cell RNA-seq and ATAC-seq data.* Genome Biol, 2022. **23**(1): p. 20.
52. Cao, J., et al., *Joint profiling of chromatin accessibility and gene expression in thousands of single cells.* Science, 2018. **361**(6409): p. 1380-1385.
53. Zhu, C., et al., *An ultra high-throughput method for single-cell joint analysis of open chromatin and transcriptome.* Nat Struct Mol Biol, 2019. **26**(11): p. 1063-1070.
54. Chen, S., B.B. Lake, and K. Zhang, *High-throughput sequencing of the transcriptome and chromatin accessibility in the same cell.* Nat Biotechnol, 2019. **37**(12): p. 1452-1457.
55. Ma, S., et al., *Chromatin Potential Identified by Shared Single-Cell Profiling of RNA and Chromatin.* Cell, 2020. **183**(4): p. 1103-1116 e20.
56. Linjing, L., et al., *A pre-trained large language model for translating single-cell transcriptome to proteome.* bioRxiv, 2023: p. 2023.07.04.547619.
57. Cancer Genome Atlas Research, N., *Comprehensive molecular characterization of clear cell renal cell carcinoma.* Nature, 2013. **499**(7456): p. 43-9.
58. Ciriello, G., et al., *Comprehensive Molecular Portraits of Invasive Lobular Breast Cancer.* Cell, 2015. **163**(2): p. 506-19.
59. Fishbein, L., et al., *Comprehensive Molecular Characterization of Pheochromocytoma and Paraganglioma.* Cancer Cell, 2017. **31**(2): p. 181-193.
60. Kahles, A., et al., *Comprehensive Analysis of Alternative Splicing Across Tumors from 8,705 Patients.* Cancer Cell, 2018. **34**(2): p. 211-224 e6.
61. Cao, L., et al., *Proteogenomic characterization of pancreatic ductal adenocarcinoma.* Cell, 2021. **184**(19): p. 5031-5052 e26.
62. Dou, Y., et al., *Proteogenomic Characterization of Endometrial Carcinoma.* Cell, 2020. **180**(4): p. 729-748 e26.
63. Gillette, M.A., et al., *Proteogenomic Characterization Reveals Therapeutic Vulnerabilities in Lung Adenocarcinoma.* Cell, 2020. **182**(1):

p. 200-225 e35.
64. Krug, K., et al., *Proteogenomic Landscape of Breast Cancer Tumorigenesis and Targeted Therapy.* Cell, 2020. **183**(5): p. 1436-1456 e31.
65. Petralia, F., et al., *Integrated Proteogenomic Characterization across Major Histological Types of Pediatric Brain Cancer.* Cell, 2020. **183**(7): p. 1962-1985 e31.
66. Satpathy, S., et al., *A proteogenomic portrait of lung squamous cell carcinoma.* Cell, 2021. **184**(16): p. 4348-4371 e40.
67. Wang, L.B., et al., *Proteogenomic and metabolomic characterization of human glioblastoma.* Cancer Cell, 2021. **39**(4): p. 509-528 e20.
68. Encyclopedia, T.C.C.L., et al., *Consistency of drug profiles and predictors in large-scale cancer cell line data.* Nature, 2015. **528**(7580): p. 84.
69. Nusinow, D.P., et al., *Quantitative Proteomics of the Cancer Cell Line Encyclopedia.* Cell, 2020. **180**(2): p. 387-402 e16.
70. Pietzak, E.J., et al., *Genomic Differences Between "Primary" and "Secondary" Muscle-invasive Bladder Cancer as a Basis for Disparate Outcomes to Cisplatin-based Neoadjuvant Chemotherapy.* Eur Urol, 2019. **75**(2): p. 231-239.
71. Hao, Y., et al., *Integrated analysis of multimodal single-cell data.* Cell, 2021. **184**(13): p. 3573-3587 e29.
72. Peterson, V.M., et al., *Multiplexed quantification of proteins and transcripts in single cells.* Nat Biotechnol, 2017. **35**(10): p. 936-939.
73. Stoeckius, M., et al., *Simultaneous epitope and transcriptome measurement in single cells.* Nat Methods, 2017. **14**(9): p. 865-868.
74. Cheng, S., et al., *A pan-cancer single-cell transcriptional atlas of tumor infiltrating myeloid cells.* Cell, 2021. **184**(3): p. 792-809 e23.
75. Hao, M., et al., *Large-scale foundation model on single-cell transcriptomics.* Nat Methods, 2024. **21**(8): p. 1481-1491.
76. Liu, Q., et al., *DeepCDR: a hybrid graph convolutional network for predicting cancer drug response.* Bioinformatics, 2020. **36**(Suppl_2): p. i911-i918.
77. Roohani, Y., K. Huang, and J. Leskovec, *Predicting transcriptional outcomes of novel multigene perturbations with GEARS.* Nat Biotechnol, 2023.
78. Tang, W., et al. *Single-cell multimodal prediction via transformers*. in *Proceedings of the 32nd ACM International Conference on Information and Knowledge Management*. 2023.
79. Yang, X., et al., *GeneCompass: deciphering universal gene regulatory mechanisms with a knowledge-informed cross-species foundation model.* Cell Res, 2024. **34**(12): p. 830-845.
80. Qiao, Y., et al., *Synergistic activation of inflammatory cytokine genes by interferon-gamma-induced chromatin remodeling and toll-like*

receptor signaling. Immunity, 2013. **39**(3): p. 454-69.

81. Srivatsan, S.R., et al., *Massively multiplex chemical transcriptomics at single-cell resolution.* Science, 2020. **367**(6473): p. 45-51.
82. Garipler, G., et al., *The BTB transcription factors ZBTB11 and ZFP131 maintain pluripotency by repressing pro-differentiation genes.* Cell Rep, 2022. **38**(11): p. 110524.
83. Bian, H., et al. *scMulan: a multitask generative pre-trained language model for single-cell analysis*. in *International Conference on Research in Computational Molecular Biology*. 2024. Springer.
84. Simonson, B., et al., *Single-nucleus RNA sequencing in ischemic cardiomyopathy reveals common transcriptional profile underlying end-stage heart failure.* Cell Rep, 2023. **42**(2): p. 112086.
85. Suo, C., et al., *Mapping the developing human immune system across organs.* Science, 2022. **376**(6597): p. eabo0510.
86. Lotfollahi, M., et al., *Mapping single-cell data to reference atlases by transfer learning.* Nat Biotechnol, 2022. **40**(1): p. 121-130.
87. Rosen, Y., et al., *Universal cell embeddings: A foundation model for cell biology.* bioRxiv, 2023: p. 2023.11. 28.568918.
88. Dominguez Conde, C., et al., *Cross-tissue immune cell analysis reveals tissue-specific features in humans.* Science, 2022. **376**(6594): p. eabl5197.
89. Speranza, E., et al., *Single-cell RNA sequencing reveals SARS-CoV-2 infection dynamics in lungs of African green monkeys.* Sci Transl Med, 2021. **13**(578).
90. Hilton, H.G., et al., *Single-cell transcriptomics of the naked mole-rat reveals unexpected features of mammalian immunity.* PLoS Biol, 2019. **17**(11): p. e3000528.
91. Yamagata, M., W. Yan, and J.R. Sanes, *A cell atlas of the chick retina based on single-cell transcriptomics.* Elife, 2021. **10**.
92. Orozco, L.D., et al., *Integration of eQTL and a Single-Cell Atlas in the Human Eye Identifies Causal Genes for Age-Related Macular Degeneration.* Cell Rep, 2020. **30**(4): p. 1246-1259 e6.
93. Zhao, S., J. Zhang, and Z. Nie, *Large-scale cell representation learning via divide-and-conquer contrastive learning.* arXiv preprint arXiv:2306.04371, 2023.
94. Aissa, A.F., et al., *Single-cell transcriptional changes associated with drug tolerance and response to combination therapies in cancer.* Nat Commun, 2021. **12**(1): p. 1628.
95. Sharma, A., et al., *Longitudinal single-cell RNA sequencing of patient-derived primary cells reveals drug-induced infidelity in stem cell*

hierarchy. Nat Commun, 2018. **9**(1): p. 4931.
96. Ravasio, A., et al., *Single-cell analysis of EphA clustering phenotypes to probe cancer cell heterogeneity.* Commun Biol, 2020. **3**(1): p. 429.
97. Suphavilai, C., et al., *Predicting heterogeneity in clone-specific therapeutic vulnerabilities using single-cell transcriptomic signatures.* Genome Med, 2021. **13**(1): p. 189.
98. Barretina, J., et al., *The Cancer Cell Line Encyclopedia enables predictive modelling of anticancer drug sensitivity.* Nature, 2012. **483**(7391): p. 603-7.
99. Iorio, F., et al., *A Landscape of Pharmacogenomic Interactions in Cancer.* Cell, 2016. **166**(3): p. 740-754.
100. Xiong, L., T. Chen, and M. Kellis. *scCLIP: Multi-modal Single-cell Contrastive Learning Integration Pre-training*. in *NeurIPS 2023 AI for Science Workshop*.
101. Cao, J., et al., *A human cell atlas of fetal gene expression.* Science, 2020. **370**(6518).
102. Domcke, S., et al., *A human cell atlas of fetal chromatin accessibility.* Science, 2020. **370**(6518).
103. Anderson, A.G., et al., *Single nucleus multiomics identifies ZEB1 and MAFB as candidate regulators of Alzheimer's disease-specific cis-regulatory elements.* Cell Genom, 2023. **3**(3): p. 100263.
104. Shen, H., et al., *A universal approach for integrating super large-scale single-cell transcriptomes by exploring gene rankings.* Brief Bioinform, 2022. **23**(2).
105. Kang, H.M., et al., *Multiplexed droplet single-cell RNA-sequencing using natural genetic variation.* Nat Biotechnol, 2018. **36**(1): p. 89-94.
106. Guo, X., et al., *Global characterization of T cells in non-small-cell lung cancer by single-cell sequencing.* Nat Med, 2018. **24**(7): p. 978-985.
107. Zheng, C., et al., *Landscape of Infiltrating T Cells in Liver Cancer Revealed by Single-Cell Sequencing.* Cell, 2017. **169**(7): p. 1342-1356 e16.
108. Wen, H., et al., *CellPLM: pre-training of cell language model beyond single cells.* bioRxiv, 2023: p. 2023.10. 03.560734.
109. Li, Y., et al., *Single-Cell Transcriptome Analysis Reveals Dynamic Cell Populations and Differential Gene Expression Patterns in Control and Aneurysmal Human Aortic Tissue.* Circulation, 2020. **142**(14): p. 1374-1388.

110. Schirmer, L., et al., *Neuronal vulnerability and multilineage diversity in multiple sclerosis.* Nature, 2019. **573**(7772): p. 75-82.
111. Wang, Y., et al., *scGREAT: Transformer-based deep-language model for gene regulatory network inference from single-cell transcriptomics.* iScience, 2024. **27**(4): p. 109352.
112. Chu, L.F., et al., *Single-cell RNA-seq reveals novel regulators of human embryonic stem cell differentiation to definitive endoderm.* Genome Biol, 2016. **17**(1): p. 173.
113. Mora-Bermudez, F., et al., *Differences and similarities between human and chimpanzee neural progenitors during cerebral cortex development.* Elife, 2016. **5**.
114. Camp, J.G., et al., *Multilineage communication regulates human liver bud development from pluripotency.* Nature, 2017. **546**(7659): p. 533-538.
115. Shalek, A.K., et al., *Single-cell RNA-seq reveals dynamic paracrine control of cellular variation.* Nature, 2014. **510**(7505): p. 363-9.
116. Hayashi, T., et al., *Single-cell full-length total RNA sequencing uncovers dynamics of recursive splicing and enhancer RNAs.* Nat Commun, 2018. **9**(1): p. 619.
117. Nestorowa, S., et al., *A single-cell resolution map of mouse hematopoietic stem and progenitor cell differentiation.* Blood, 2016. **128**(8): p. e20-31.
118. Amara-Belgadi, S., et al., *BIOFORMERS: A SCALABLE FRAMEWORK FOR EXPLORING BIOSTATES USING TRANSFORMERS.* bioRxiv, 2023: p. 2023.11. 29.569320.
119. Zheng, G.X., et al., *Massively parallel digital transcriptional profiling of single cells.* Nature communications, 2017. **8**(1): p. 14049.
120. Kalfon, J., et al., *scPRINT: pre-training on 50 million cells allows robust gene network predictions.* bioRxiv, 2024: p. 2024.07. 29.605556.
121. Kong, L., et al., *The landscape of immune dysregulation in Crohn's disease revealed through single-cell transcriptomic profiling in the ileum and colon.* Immunity, 2023. **56**(2): p. 444-458 e5.
122. Wang, S.K., et al., *Single-cell multiome of the human retina and deep learning nominate causal variants in complex eye diseases.* Cell Genom, 2022. **2**(8).
123. Marshall, J.L., et al., *High-resolution Slide-seqV2 spatial transcriptomics enables discovery of disease-specific cell neighborhoods and pathways.* iScience, 2022. **25**(4): p. 104097.

124. Dixit, A., et al., *Perturb-Seq: Dissecting Molecular Circuits with Scalable Single-Cell RNA Profiling of Pooled Genetic Screens.* Cell, 2016. **167**(7): p. 1853-1866 e17.
125. Park, P.J., *ChIP-seq: advantages and challenges of a maturing technology.* Nat Rev Genet, 2009. **10**(10): p. 669-80.
126. Burclaff, J., et al., *A Proximal-to-Distal Survey of Healthy Adult Human Small Intestine and Colon Epithelium by Single-Cell Transcriptomics.* Cell Mol Gastroenterol Hepatol, 2022. **13**(5): p. 1554-1589.
127. van Zyl, T., et al., *Cell atlas of the human ocular anterior segment: Tissue-specific and shared cell types.* Proc Natl Acad Sci U S A, 2022. **119**(29): p. e2200914119.
128. Joseph, D.B., et al., *Single-cell analysis of mouse and human prostate reveals novel fibroblasts with specialized distribution and microenvironment interactions.* J Pathol, 2021. **255**(2): p. 141-154.
129. Mao, Y., et al., *Phenotype prediction from single-cell RNA-seq data using attention-based neural networks.* Bioinformatics, 2024. **40**(2).
130. julian.knight@well.ox.ac.uk, C.O.-M.-o.B.A.C.E.a. and C.O.-M.-o.B.A. Consortium, *A blood atlas of COVID-19 defines hallmarks of disease severity and specificity.* Cell, 2022. **185**(5): p. 916-938 e58.
131. Stephenson, E., et al., *Single-cell multi-omics analysis of the immune response in COVID-19.* Nat Med, 2021. **27**(5): p. 904-916.
132. Ren, X., et al., *COVID-19 immune features revealed by a large-scale single-cell transcriptome atlas.* Cell, 2021. **184**(7): p. 1895-1913 e19.
133. Theus, A., et al., *CancerFoundation: A single-cell RNA sequencing foundation model to decipher drug resistance in cancer.* bioRxiv, 2024: p. 2024.11. 01.621087.
134. Neftel, C., et al., *An Integrative Model of Cellular States, Plasticity, and Genetics for Glioblastoma.* Cell, 2019. **178**(4): p. 835-849 e21.
135. Wissel, D., et al., *Survboard: standardised benchmarking for multi-omics cancer survival models.* bioRxiv, 2022: p. 2022.11. 18.517043.
136. Cancer Genome Atlas Research, N., et al., *The Cancer Genome Atlas Pan-Cancer analysis project.* Nat Genet, 2013. **45**(10): p. 1113-20.
137. Querfurth, B.v., et al., *mcBERT: Patient-Level Single-cell Transcriptomics Data Representation.* bioRxiv, 2024: p. 2024.11. 04.621897.
138. Chaffin, M., et al., *Single-nucleus profiling of human dilated and hypertrophic cardiomyopathy.* Nature, 2022. **608**(7921): p. 174-180.
139. Koenig, A.L., et al., *Single-cell transcriptomics reveals cell-type-specific diversification in human heart failure.* Nat Cardiovasc Res, 2022. **1**(3): p. 263-280.
140. Reichart, D., et al., *Pathogenic variants damage cell composition and single cell transcription in cardiomyopathies.* Science, 2022.

377(6606): p. eabo1984.
141. Kuppe, C., et al., *Spatial multi-omic map of human myocardial infarction.* Nature, 2022. **608**(7924): p. 766-777.
142. Lake, B.B., et al., *An atlas of healthy and injured cell states and niches in the human kidney.* Nature, 2023. **619**(7970): p. 585-594.
143. Kuppe, C., et al., *Decoding myofibroblast origins in human kidney fibrosis.* Nature, 2021. **589**(7841): p. 281-286.
144. Muto, Y., et al., *Defining cellular complexity in human autosomal dominant polycystic kidney disease by multimodal single cell analysis.* Nat Commun, 2022. **13**(1): p. 6497.
145. Wilson, P.C., et al., *Multimodal single cell sequencing implicates chromatin accessibility and genetic background in diabetic kidney disease progression.* Nat Commun, 2022. **13**(1): p. 5253.
146. Muto, Y., et al., *Single cell transcriptional and chromatin accessibility profiling redefine cellular heterogeneity in the adult human kidney.* Nat Commun, 2021. **12**(1): p. 2190.
147. Perez, R.K., et al., *Single-cell RNA-seq reveals cell type-specific molecular and genetic associations to lupus.* Science, 2022. **376**(6589): p. eabf1970.
148. Yoshida, M., et al., *Local and systemic responses to SARS-CoV-2 infection in children and adults.* Nature, 2022. **602**(7896): p. 321-327.
149. Sikkema, L., et al., *An integrated cell atlas of the lung in health and disease.* Nat Med, 2023. **29**(6): p. 1563-1577.
150. Schaar, A., et al., *Nicheformer: a foundation model for single-cell and spatial omics. 2024.* Preprint at bioRxiv, 2024. **4**: p. 589472.
151. Yao, Z., et al., *A high-resolution transcriptomic and spatial atlas of cell types in the whole mouse brain.* Nature, 2023. **624**(7991): p. 317-332.
152. He, S., et al., *High-plex multiomic analysis in FFPE at subcellular level by spatial molecular imaging. bioRxiv 467020.* 2021.
153. Wen, H., et al., *Single cells are spatial tokens: Transformers for spatial transcriptomic data imputation.* arXiv preprint arXiv:2302.03038, 2023.
